# Pyridone alkaloids from an Antarctic endolichenic *Tolypocladium* sp.

**DOI:** 10.1007/s13659-026-00607-1

**Published:** 2026-04-01

**Authors:** Shasha Li, Ting Yu, Jianju Feng, Yue Shang, Tao Zhang, Shuzhen Chen, Liyan Yu, Maoluo Gan

**Affiliations:** 1 Beijing Key Laboratory of Technology and Application for Anti-Infective New Drugs Research and Development, Institute of Medicinal Biotechnology, Chinese Academy of Medical Sciences and Peking Union Medical College, Beijing, 100050 China; 2 China Pharmaceutical Culture Collection, Institute of Medicinal Biotechnology, Chinese Academy of Medical Sciences and Peking Union Medical College, Beijing, 100050 China

**Keywords:** Pyridone alkaloids, Tetramic acid, Antarctic microbes, Endolichenic fungus, *Tolypocladium* sp., Antimicrobial activities

## Abstract

Three new pyridone alkaloids, tolypyrone A (**1**), tolypyrosides A (**2**) and B (**3**), and a new tetramic acid 2*′*-epitolypoalbin (**4**), along with ten known compounds, were obtained from *Tolypocladium* sp. CPCC 401485 isolated from an unidentified Antarctic lichen specimen. Structural elucidation of new compounds was achieved by analyses of HRESIMS and NMR spectroscopic data combined with X-ray crystallography, advanced Marfey’s method, and ECD calculation. Tolypyrone A (**1**) was the first 4-pyridone alkaloid with an unusual octahydro-1*H*-isochromeno[3,4-*b*]pyridin-4-one triheterocyclic ring system. Compounds **1**, **4** and **6** exhibited antimicrobial activities with the minimum inhibition concentration of 16−64 μg/mL.

**Graphical Abstract:**

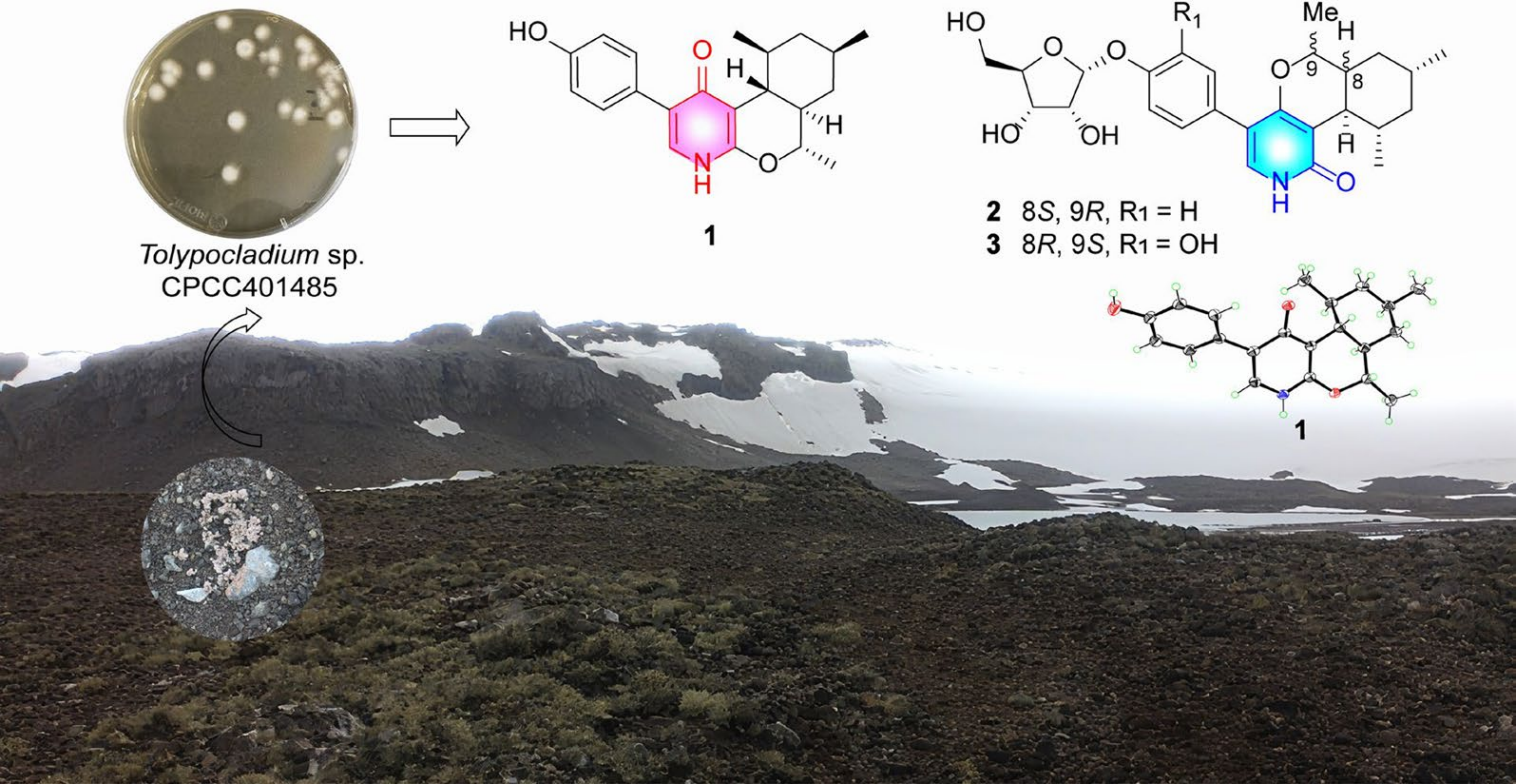

**Supplementary Information:**

The online version contains supplementary material available at 10.1007/s13659-026-00607-1.

## Introduction

Extremophile organisms represent a valuable source of bioactive compounds for medicinal application [1]. The continent of Antarctica is widely recognized by the scientific community as the most extreme region on Earth, distinguished by its unparalleled meteorological conditions. Microorganisms surviving in Antarctica are anticipated to have evolved a variety of strategies to maintain activity and metabolic function against the unfavorable environmental conditions, such as very low temperature, intense UV-radiation, elevated salinity, and nutritional deficiency [2]. Among the strategies Antarctic microorganisms used to interact with the environment, specialized metabolites are particularly important for their survival under the harsh conditions [3]. The genomes of Antarctic microbes have been shaped by environmental pressures and long-term isolation, indicating that they may biosynthesize unique metabolites with novel structures and specific bioactivity. A growing number of diverse natural products exhibiting various bioactivities have been identified from Antarctic microbes [1, 4–6].

2-Pyridone alkaloids are a family of compounds featuring a 2-pyridone core, most of which are produced by filamentous fungi [7–9]. They show a variety of bioactivities, such as antibacterial (leporin A) [10], antifungal (ilicicolin H) [11], antimalarial (torrubiellone A and cordypyridone A) [12, 13], and antitumor (TMC-69 and maximiscin) [14] activities. This family of compounds exhibit diverse structures resulted from modification of the pyridone core, including 4-hydroxy-3-acyl/alkyl/alkoxy, 4-hydroxy-5-alkyl/aryl, 4-hydroxy-6-alkyl, and 4-oxy-3-alkoxy/alkyl [7, 8]. Due to their diverse bioactivities, 2-pyridones have attracted much attention as fascinating lead compounds for drug development and agrochemical industries [7, 8].

**Fig. 1:**
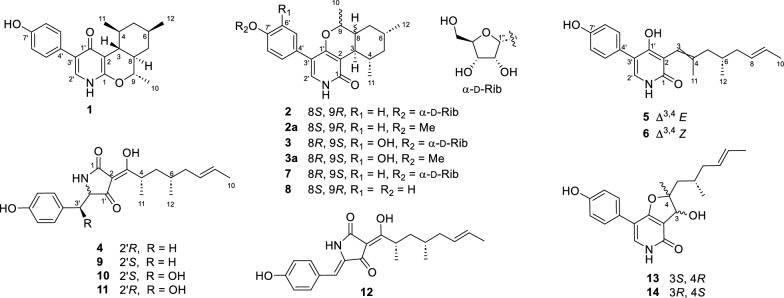
Chemical structures of compounds **1**–**14**

During our ongoing efforts to discover novel antibiotics from fungi [15–17], the cultures of *Tolypocladium sp.* CPCC 401485 obtained from an unidentified lichen, were found to show significant antimicrobial activities. Bioassay-guided isolation and chemical characterization yielded three new pyridones (**1**−**3**) and a new tetramic acid (**4**), as well as ten related analogues (**5**−**14)** (Fig. 1). Compound **1** represented the first case of 4-pyridone natural products with a novel octahydro-1*H*-isochromeno[3,4-*b*]pyridin-4-one triheterocyclic ring skeleton. The obtained compounds were assessed for antimicrobial activities against bacterial and fungal pathogens and cytotoxicities against cancerous cells.

## Results and discussion

Compound **1** was isolated and characterized as colorless, needle-like crystals. Analysis of HRESIMS data allowed to assign the molecular formula as C_21_H_25_NO_3_. Its IR spectrum exhibited an intense absorption peak at 1693 cm^−1^ attributed to an unsaturated carbonyl group. The ^1^H NMR spectrum of **1** revealed an A_2_B_2_ pattern of a *para*-disubstituted aromatic ring (*δ*_H_ 7.33 and 6.70, d, each 2H) and an isolated olefinic methine at *δ*_H_ 7.19 (s) (Table 1). Two exchangeable protons signals, observed in DMSO-*d*_6_, were assigned to one phenolic hydroxy proton (*δ*_H_ 9.35) and one amine proton (*δ*_H_ 11.36). The ^13^C NMR and HSQC spectra indicated one carbonyl at *δ*_C_ 178.1, additional five quaternary *sp*^2^ carbons, five *sp*^2^ methines, two *sp*^3^ methylenes, five aliphatic methines, and three methyls. These data indicated that **1** was an analogue of the pyridone alkaloid tolypyridone A [18].

**Table 1:** ^1^H (600 MHz) and ^13^C NMR (150 MHz) Data for Compounds** 1**−**3**^a^

No.	**1**^b^		**2**^c^		**3**^b^	
	*δ*_C,_ type	*δ*_H_, mult. (*J* in Hz)	*δ*_C,_ type	*δ*_H_, mult. (*J* in Hz)	*δ*_C,_ type	*δ*_H_, mult. (*J* in Hz)
1	155.2, C		165.4, C		162.8, C	
2	107.3, C		112.3, C		111.1, C	
3	43.0. CH	2.26, t (10.2)	38.6, CH	2.60, dd (10.8, 4.2)	43.5, CH	2.11, t (10.2)
4	41.1, CH	1.44, m	37.7, CH	1.66, m	39.3, CH	1.66, m
5α	45.8, CH_2_	0.96, overlap	45.7, CH_2_	0.91, overlap	45.6, CH_2_	0.93, ddd (12.0, 10.0, 10.0)
5β		1.66, brd (13.2)		1.73, brd (13.8)		1.68, ddd (11.5, 3.0, 3.0)
6	32.6, CH	1.62, m	28.0, CH	1.67, m	32.1, CH	1.61, m
7α	36.6, CH_2_	0.83, dt (12.0, 10.2)	37.0, CH_2_	1.36, ddd (13.8, 13.2, 4.2)	36.9, CH_2_	0.80, ddd (12.0, 10.0, 10.0)
7β		1.76, brd (10.2)		1.88, brd (13.8)		1.77, ddd (12.0, 2.5, 2.5)
8	49.4, CH	1.42, qd (10.2, 2.4)	40.1, CH	1.70, m	48.7, CH	1.42, qd (10.2, 2.4)
9	78.4, CH	3.78, m	74.6, CH	4.68, dq (12.0, 6.0)	77.6, CH	3.68, dq (10.2, 6.0)
10	18.7, CH_3_	1.29, d (6.0)	20.1, CH_3_	1.33, d (6.0)	18.9, CH_3_	1.22, d (6.6)
11	23.1, CH_3_	0.97, d (6.6)	21.0, CH_3_	0.96, d (6.6)	22.9, CH_3_	1.03, d (6.6)
12	22.5, CH_3_	0.92, d (6.0)	23.2, CH_3_	0.92, d (6.6)	22.4, CH_3_	0.92, d (6.6)
1′	178.1, C		162.3, C		162.3, C	
2′	129.0, CH	7.19, s	132.2, CH	7.16, s	130.7, CH	7.05, brs
3′	124.3, C		116.8, C		112.9, C	
4′	127.1, C		129.5, C		128.7, C	
5′	129.5, CH	7.33, d (7.8)	131.2, CH	7.33, d (8.4)	116.2, CH	6.84, d (1.8)
6′	114.7, CH	6.70, d (7.8)	117.8, CH	7.14, d (8.4)	146.6, C	
7′	156.1, C		158.1, C		143.3, C	
8′	114.7, CH	6.70, d (7.8)	117.8, CH	7.14, d (8.4)	115.7, CH	7.05, d (8.4)
9′	129.5, CH	7.33, d (7.8)	131.2, CH	7.33, d (8.4)	119.5, CH	6.75, dd (8.4, 2.4)
1′′			102.4, CH	5.65, d (4.2)	101.1, CH	5.51, d (4.2)
2′′			73.4, CH	4.20, dd (6.6, 4.2)	72.2, CH	4.05, brs
3′′			71.2, CH	4.10, dd (6.6, 3.0)	69.4, CH	3.92, brd (6.0)
4′′			87.5, CH	4.15, dt (4.2, 3.0)	86.6, CH	3.98, q (3.6)
5′′			63.2, CH_2_	3.71, dd (12.0, 4.2);	61.6, CH_2_	3.46, t (3.6)
				3.66, dd (12.0, 4.2)		
NH		11.36, brs				11.01, s
OH-6′						8.61, brs
OH-7′		9.35, brs				

^a^ The assignments were made by 2D NMR (COSY, HSQC, HMBC and ROESY) data

^b^ Recorded in DMSO-*d*_6_

^c^ Recorded in CD_3_OD

**Fig. 2:**
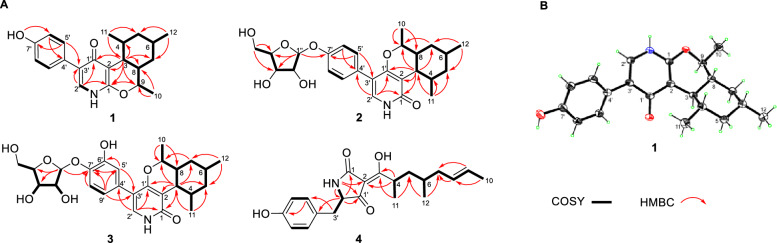
**A** The key 2D correlations of compounds **1−4**. **B** X-ray crystallographic structure of 1

**Fig. 3:**
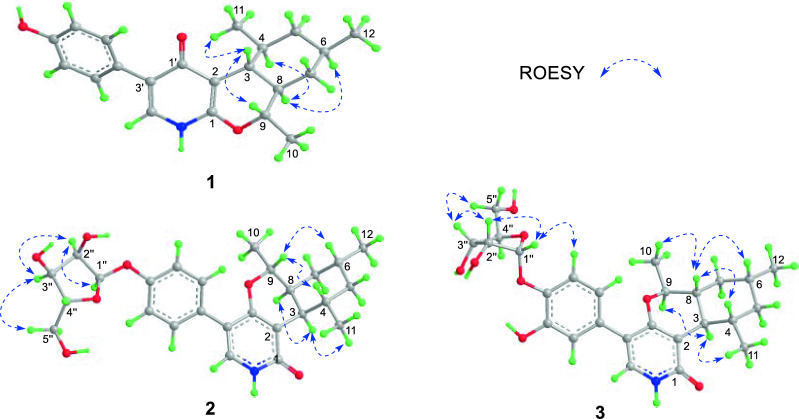
The ROESY correlations (blue dashed line) of **1−3**

The COSY correlations revealed three spin systems (Fig. 2A), including one aromatic ring; one tetra-substituted cyclohexane ring involving the aliphatic protons from C-3 to C-9 with three methyl substituents at C-4, C-6, and C-9; and one enamine fragment containing H-2′ and NH. In the HMBC experiment, H-3 showed correlations with C-1 (*δ*_C_ 155.2), C-2 (*δ*_C_ 107.3), C-8, and C-9 while H-9 was correlated to C-1. These data revealed the presence of a dihydropyran ring fused with the cyclohexane ring to form an isochromene moiety. Further, the dihydropyran ring was deduced to be fused with a 4-pyridone ring according to the cross-peaks of H-3/C-1′ (*δ*_C_ 178.1) and H-2′/C-1′, C-3′, and C-1. A 7′-hydroxybenzene moiety was assigned at C-3′ of the pyridone moiety based on the cross-peaks of OH with C-6′, C-7′, and C-8′ as well as H_2_-5′, 9′ with C-3′, completing the establishment of the whole structure. The relative configuration was elucidated with help of the coupling constants of relevant protons and ROESY experiment (Fig. 3). The large value for ^3^*J*_H-3,4_ and ^3^*J*_H-3,8_ (10.2 Hz) indicated their *trans* 1,3-diaxial relationships. In addition, H-3 showed ROESY cross-peaks with H-9 and H_3_-11, revealing that they have the same orientation on the isochromene skeleton. On the contrary, the protons H-4, H-6, and H-8 were assigned on the opposite side of the cyclohexane moiety according to cross-peaks of H-8/H-4 and H-6.

**Fig. 4:**
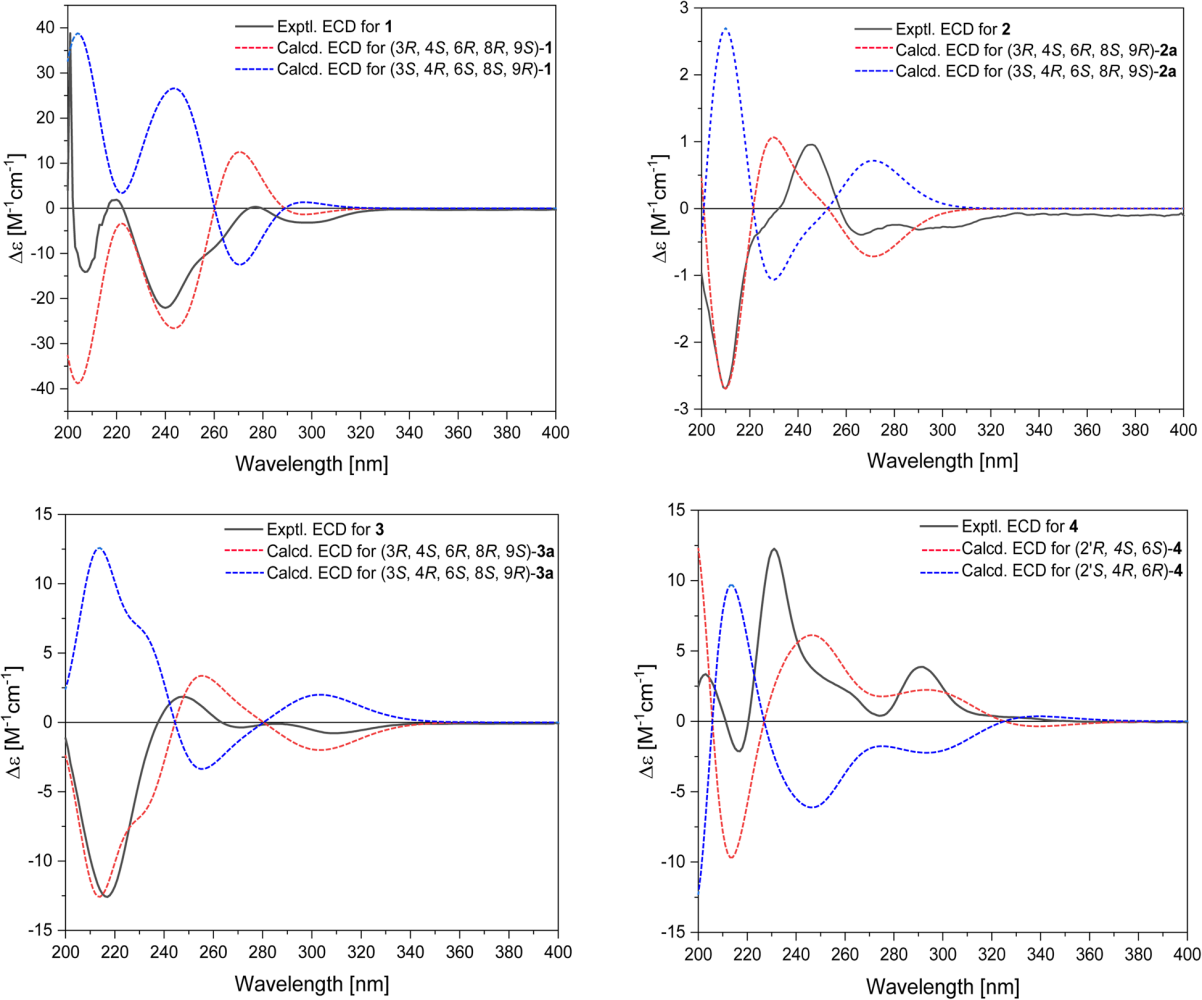
Comparison of the experimental and calculated ECD spectra for compounds **1−4**

Very recently, Shim group reported the identification of tolypyridinol A [19], wherein the core structure was determined as 4-hydroxypyridine. Compared to those of tolypyridinol A, the NMR data of **1** recorded in the same solvent CD_3_OD (Table S2) showed significant differences for H-2′, C-1′, and C-2′, indicative of a distinct core structure for **1**. Furthermore, the COSY cross-peaks between NH (*δ*_H_ 11.36) and H-2′ (*δ*_H_ 7.19) recorded in DMSO-*d*_6_ for **1** unequivocally precluded the possibility of a pyridine ring in **1**. The structure was finally consolidated by X-ray crystallographic analysis (Fig. **2**B), which indicated the absolute configuration as 3*R*, 4*S*, 6*R*, 8*R*, 9*S* by a small value of the refined Flack parameter − 0.06 (9). This was further verified by the ECD calculation (Fig. 4) [20]. Thus, **1** was elucidated as a novel 4-pyridone alkaloid and named tolypyrone A.

Compound **2** was obtained as a white noncrystalline powder and analyzed for C₂₆H₃₃NO₇ by HRESIMS and NMR data. The ^1^H and ^13^C NMR spectra of **2** displayed signals closely similar to those of the co-isolated trichodin B (**7**) [21], including a ribofuranosyl moiety, a *para*-disubstituted aromatic ring, and a substituted cyclohexane ring with three methyls. However, the resonance for H-3 appeared as a double doublet at *δ*_H_ 2.60 (*J* = 10.8, 4.2 Hz in CD_3_OD) in **2** while it showed a triplet at *δ*_H_ 2.26 (*J* = 10.1 Hz) in **7**. A combination of 2D NMR data led to assignment of the identical planar structure of **2** with that of **7**. The large coupling *J*_H-8,9_ (12.0 Hz) and *J*_H-3,4_ (10.8 Hz) revealed their 1,3-diaxial positions whereas the small value of *J*_H-3,8_ (4.2 Hz) suggested a *cis* fusion between the dihydropyran and cyclohexane rings. ROESY correlations of H-3/H-8 and H_3_-11 revealed that they were *cis* oriented on the cyclohexane moiety. On the contrary, H-9 exhibited ROESY cross-peaks with H-4 and H-6, suggesting that they positioned on the other side of the ring skeleton. Therefore, the relative configuration of the aglycone moiety in **2** was determined as 3R^*^, 4S^*^, 6R^*^, 8S^*^, 9R^*^, consistent with that of tolypyridone L [22] (**8**, also named as tolypyridone I by Jung et al. [23]). The resonance for C-1′′ (*δ*_C_ 102.4 in CD_3_OD; *δ*_C_ 100.4 in DMSO-*d*_6_) and the relatively large value for *J*_H-1′′,2′′_ (4.2 Hz) were suggestive of an α- anomeric configuration for the ribofuranosyl [24–26], which was consistent with the cross-peaks between H-1′′/H-2′′, H-2′′/H-3′′, and H-3′′/H-5′′ observed in the ROESY experiment. Of note, the ribofuranosyl in trichodin B (**7**) was previously assigned to be of *β*- anomeric configuration [21]. However, the typical values of *J*_H-1′′,2′′_ (4.2 Hz) and *δ*_C-1′′_ (102.4 ppm in CD_3_OD) reported for **7** unambiguously indicated that the ribofuranosyl in trichodin B (**7**) should be of *α*-configuration according to those of the synthetic *O*-aryl and *O*-alky ribofuranosides (α-: *J*_H-1′′,2′′_ 4.0 Hz, *δ*_C-1′′_ 102 ± 1.0; *β*-: *J*_H-1′′,2′′_ 0 Hz, *δ*_C-1′′_ 107 ± 1.0) [24, 25].

Compound **2** displayed an ECD curve similar to tolypyridone L [22, 23], indicating that the aglycone of **2** had the same absolute configuration as tolypyridone L. This deduction was further verified by ECD calculation, wherein the calculated spectrum for the truncated model compound 3*R*, 4*S*, 6*R*, 8*S*, 9*R-***2a** matched well with the experimental for **2**, leading to assignment of the absolute stereochemistry for **2***.* The ribosyl residue was assigned as d-configuration by using the naphthimidazole derivatization method followed by chiral LC–MS analysis (Figure S3) [27]. Thus, **2** was elucidated as the 7′-*O*-α-d-ribofuranosyl-tolypyridone L and given the trivial name tolypyroside A.

Compound **3** possessed the molecular formula C_26_H_33_NO_8_ from HRESIMS, containing one oxygen atom more than **2**. In contrast to **2**, the NMR data revealed an ABX system of a trisubstituted aromatic ring and an exchangeable proton at *δ*_H_ 8.61 corresponding to a phenol group in **3**. The heteronuclear correlations from H-5′ to C-3′, C-6′ (*δ*_C_ 146.6), and C-7′ (*δ*_C_ 143.3), and H-9′ to C-3′, C-5′, and C-7′ revealed the presence of a 6′,7′-dioxygenated aromatic ring in **3** in place of the 7′-oxygenated aromatic ring in **2**. Specifically, C-7′ was deduced to be attached with an *O*-ribofuranosyl residue by the cross-peaks from H-1′′ to C-7′ whereas the chemical shift (*δ*_C_ 146.6) of C-6′ suggested that it was substituted by a hydroxy group. The large coupling constant of ^3^*J*_H-3/8_ and ^3^*J*_H-3/4_ (both 10.2 Hz) revealed their *trans* relationships. Analysis of ROESY data revealed that the protons H-4, H-6, and H-8 and the methyl group at C-9 positioned on the same orientation of the ring skeleton whereas H-3, H-9, and the methyl group at C-4 were on the opposite orientation. The ribofuranosyl moiety in **3** was assigned as α-D-configuration according to the resonances for H-1′′ and C-1′′, ROESY data, and chiral HPLC analysis of the hydrolysate. The aglycone was determined to have the 3*R*, 4*S*, 6*R*, 8*R*, 9*S* configuration by ECD calculation (Fig. 4)*.*

Compound **4** was analyzed for the molecular formula C₂₁H₂₇NO₄ by HRESIMS and NMR data. The NMR spectra of **4** measured in CDCl_3_ (Tables 2 and S4) were almost identical with those of the co-isolated tolypoalbin (**9**) [28], showing the resonances characteristic for a tetramic acid (*δ*_C_ 175.5, 100.6, 193.7, 194.1). However, the optical rotation ($[\alpha]^{25}_{\text D}$ + 156.2 in MeOH) and the ECD pattern (Figure S9) of **4** were totally opposite to those of **9** ($[\alpha]^{25}_{\text D}$ − 180.8 in MeOH). These data indicated that **4** was a stereoisomer of **9**. Of note, the NMR spectra of **4** (Figures S42-43) recorded in CDCl_3_, like those of 3-acyltetramic acids, showed two set of resonances corresponding to two equilibrating Δ^2,3^ geometric isomers (4:1) [27, 29]. The resonances of C-1 (*δ*_C_ 175.5) and C-3 (*δ*_C_ 193.7) suggested the major isomer with a Δ^2,3^* Z* geometry. The large value of ^3^*J*_H-8,9_ (15.0 Hz) suggested an *E* configuration for Δ^8,9^. To elucidate the absolute configuration of the pyrrolidine-2,4-dione ring, **4** was firstly subjected to alcoholysis with 1 M HCl/EtOH and then alkaline hydrolysis with 0.5 M KOH to avoid the epimerization of C-2′ [28]. The resultant hydrolysate was then applied to Marfey’s analysis [30]. Comparative LC–MS analysis of the derivatives of **4** with those of l-tyrosine (Tyr) and d-Tyr standards unambiguously determined the *R* configuration for C-2′ in **4** (Figure S2). Since previous studies revealed that the tetramic acids and 4-hydroxy pyridones share a common PKS-NRPS origin [31–33], compound **4** was deduced to possess the same configuration 4*S*, 6*S* as in** 9**. This was further confirmed by ECD calculation for **4**. Therefore, **4** was characterized as a 2′-epimer of **9** and named 2′-epitolypoalbin.

**Table 2:** ^1^H (600 MHz) and ^13^C NMR (150 MHz) Data for Compounds **4−6**^a^

No.	**4**^b^		**5**^c^		**6**^c^	
	*δ*_C,_ type	*δ*_H_, mult. (*J* in Hz)	*δ*_C,_ type	*δ*_H_, mult. (*J* in Hz)	*δ*_C,_ type	*δ*_H_, mult. (*J* in Hz)
1	175.5, C		160.2, C^*d*^		159.8, C^*d*^	
2	100.6, C		109.5, C		109.3, C	
3	193.7, C		117.1, CH	5.70, s	117.4, CH	5.69, s
4	34.1, CH	3.78, m	139.8, C		140.7, C	
5α	40.5, CH_2_	1.79, m	46.8, CH_2_	2.12, dd (13.2, 6.0)	40.2, CH_2_	1.89, dd (13.2, 6.0)
5β		1.18, m		1.88, dd (13.2, 7.8)		1.68, dd (13.8, 7.2)
6	31.4, CH	1.34, m	30.9, CH	1.68, m	30.7, CH	1.61, m
7α	40.5, CH_2_	1.97, m	39.5, CH_2_	2.05, m	39.2, CH_2_	1.84, m
7β		1.82, m		1.81, m		1.61, m
8	129.4, CH	5.36, dt (15.0, 7.2)	130.0, CH	5.41, m	129.7, CH	5.27, m
9	126.6, CH	5.42, dq (15.0, 6.0)	125.8, CH	5.43, m	125.7, CH	5.29, m
10	18.2, CH_3_	1.66, d (6.0)	17.9, CH_3_	1.63, d (4.2)	17.9, CH_3_	1.54, d (5.4)
11	18.5, CH_3_	1.19, d (6.6)	18.5, CH_3_	1.47, s	23.4, CH_3_	1.79, s
12	19.7, CH_3_	0.87, d (6.6)	19.4, CH_3_	0.85, d (6.6)	19.4, CH_3_	0.67, d (6.0)
1′	194.1, C		161.9, C^*d*^		162.1, C^*d*^	
2′	63.6, CH	3.98, dd (9.0, 3.6)	131.5, CH	7.04, s	131.4, CH	7.06, s
3′	37.3, CH_2_	3.16, dd (13.8, 3.6)	113.4, C		113.0, C	
		2.68, dd (13.8, 9.6)				
4′	127.9, C		125.6, C		125.7, C	
5′/9′	130.5, CH	7.03, d (7.8)	130.1, CH	7.19, d (8.4)	130.0, CH	7.18, d (8.4)
6′/8′	115.9, CH	6.74, d (7.8)	115.0, CH	6.74, d (8.4)	114.9, CH	6.75, d (8.4)
7′	155.1, C		156.4, C		156.3, C	
OH-7′				8.41, brs		9.48, brs
NH		5.99, brs		11.05, brs		11.03, brs

^a^ The assignments were made by 2D NMR (COSY, HSQC, HMBC and ROESY) data

^b^ Recorded in CDCl_3_

^c^ Recorded in DMSO-*d*_6_

^d^ Assignment in the same column may be exchangeable

Compounds **5** and **6** were determined as a pair of geometric isomers with two double bonds Δ^3,4^ and Δ^8,9^ in the side chain. The geometry of the double bond Δ^3,4^ was determined as *E* in **5** and *Z* in **6** by ROESY data (Figure S6). The *E* geometry of Δ^8,9^ was indicated by comparing resonances of the allylic carbons C-7 (*δ*_C_ 39.5 and 39.2) and C-10 (*δ*_C_ 17.9 and 17.9) with those reported for the 2-pyridone and tetramic acid derivatives [29, 33]. The *S* configuration was assigned for C-6, like those in tolypyridone C and **1**−**3**, according to the proposed biosynthesis of 4-hydroxy-2-pyridones [31–33].

During the preparation of this manuscript, Shim group reported two compounds tolypyridones K and L [19], whose structures were assigned to be identical with those of **5** and **6**, respectively. However, NMR data for several ^1^H and ^13^C atoms reported for tolypyridones K and L were inconsistent with those of **5** and **6** (Table S6). Elaborative examination of NMR spectra supplemented in the Shim’s paper indicated that these data for tolypyridones K and L had been incorrectly assigned. Of note, the names tolypyridones K and L had been designated for other two compounds discovered earlier by Wang group [22]. In addition, a search of 2-pyridones named beginning with the term “tolypyridone” isolated from the genus *Tolypocladium* reported in the literatures showed that each of tolypyridones I, J [22, 23] K, and L [19, 22] represented two compounds with different structures whereas tolypyridones I [23] and L [22] had the identical structures (Table S1 and Figure S1). To avoid the homonymous issues, compounds **5** and **6** were re-named as tolypyridones K2 and L2 in this study, respectively, wherein the suffix number 2 indicated a compound discovered later.

Compounds **7**−**14** were identified as trichodin B (**7**) [21], tolypyridone L (**8**) [22], tolypoalbin (**9**) [28], F-14329 (**10**) [29], chaunolidines A (**11**) and C (**12**) [29], tolypyridones E (**13**) and G (**14**) [33], respectively.

In in vitro antimicrobial assay for the obtained compounds, **1** showed inhibitory activities against *Bacillus subtilis, Staphylococcus epidermidis*, *S. aureus*, and *Candida albicans* with a minimum inhibitory concentration (MIC) at 16 μg/mL (Table 3). In contrast, the 2-pyridones **2**, **3**, **7**, and **8** were inactive (MIC > 128 μg/mL), indicating that the fusing pattern of the isochromene and pyridone rings are crucial to antimicrobial effect. Compound **6** exhibited weak activities towards *C. albicans* and other Gram-positive bacteria (MIC: 32−64 μg/mL). Interestingly, the Δ^3,4^ geometric isomer **5** was completely inactive (MIC > 128 μg/mL). All the tetramic acid analogues **4** and **9−12** showed modest antimicrobial activities against the tested microorganisms except for Gram negative bacteria. Among the isolated tetramic acids, compound **12** was the most potent with the MIC value down to 2 μg/mL, suggesting that the double bond Δ^2′,3′^ could increase the antimicrobial activity. All the compounds displayed no inhibitory activities toward *P. aeruginosa* and *E. coli.* In the cytotoxic assay, only compound **6** exhibited weak inhibitory effects against the proliferation of HCT116 and H460 cell lines with IC_50_ values of 11.8 and 14.9 μM, respectively.

**Table 3:** Antimicrobial Activities of Compounds **1**−**14** (MIC, μg/mL)

Strain	Strain no	**1**	**2**	**3**	**4**	**5**	**6**	**7**	**8**	**9**	**10**	**11**	**12**	**13**	**14**	Rif^a^	AmB^b^
*Staphylococcus aureus*	ATCC29213	16	> 128	> 128	32	> 128	32	128	> 128	32	64	32	2	128	> 128	< 0.01	nt^*c*^
*Staphylococcus epidermidis*	ATCC12228	16	> 128	> 128	32	> 128	32	> 128	> 128	32	64	32	4	128	> 128	< 0.01	nt
*Bacillus subtilis*	ATCC6633	16	> 128	> 128	64	> 128	64	128	> 128	64	128	64	8	128	> 128	< 0.01	nt
*Pseudomonas aeruginosa*	ATCC27853	> 128	> 128	> 128	> 128	> 128	> 128	> 128	> 128	> 128	> 128	> 128	> 128	> 128	> 128	16	nt
*Escherichia coli*	ATCC25922	> 128	> 128	> 128	> 128	> 128	> 128	> 128	> 128	> 128	> 128	> 128	> 128	> 128	> 128	4	nt
*Edwardsiella tarda*	QDIO-2	64	> 128	> 128	64	128	64	> 128	> 128	64	64	64	32	128	> 128	4	nt
*Edwardsiella ictaluri*	ATCC33202	32	> 128	> 128	16	> 128	64	> 128	> 128	64	32	32	16	> 128	> 128	8	nt
*Candida albicans*	ATCC10231	16	> 128	> 128	32	> 128	32	128	> 128	32	128	64	4	128	> 128	< 0.01	0.5
*Mucor racemosus*	CICC3112	> 128	> 128	> 128	64	> 128	> 128	> 128	> 128	64	128	128	8	> 128	> 128	nt	4
*Magnaporthe oryzae*	131	128	> 128	> 128	64	> 128	> 128	> 128	> 128	16	64	64	16	128	128	nt	8

^a^ Rif: Rifampicin

^b^ AmB: Amphotericin B

^c^ nt: not tested

Although hundreds of 2-pyridone natural products have been characterized from fungi [7, 9], to the best of our knowledge, 4-pyridone alkaloids have never been discovered in nature. The biosynthesis of fungus-derived 2-pyridone alkaloids has been well investigated [31–33]. The pyridone core was proved to be generated from the acyl tetramic acid precursor by oxidative ring expansion catalyzed by a P450 oxidase [31]. Compound **1** might be derived from tolypyridone C via the reduction and subsequent dehydration of the 3-keto group and the following intramolecular hetero Diels–Alder cyclization (Figure S5) [33]. Although the versatile pericyclase LepI was discovered to catalyze the hetero D-A reaction in the biosynthesis of leporin C [34], a fungal 2-pyridone congener, we did not find its homologous enzyme in the genome of strain CPCC 401485 by genomic analysis, suggesting that the formation of **1** might be achieved by a nonenzymatic process [33].

## Experimental section

### General experimental procedures

The instrumentations were described in the Supplementary Information.

### Biological material

The fungus CPCC401485 was isolated from an unidentified lichen specimen collected at Fildes Peninsula (62° 12′ 04" S, 58° 57′ 44" W), Antarctica. Its ITS gene sequence (577 nucleotides, GenBank no. PX482356) showed a similarity of 96.62% with *Tolypocladium ovalisporum* CBS 700.92 (GenBank no. NR_155019.1). In combination with morphological features, the strain was identified as belonging to the genus *Tolypocladium*.

### Fermentation and isolation

The fungus was grown on PDA Petri dishes at 18 °C for five days and then transferred into Erlenmeyer flasks (500 mL) filled with 100 mL of the liquid medium (potato starch 3.0 g/L, glucose 20 g/L). After cultivation at 18 °C with shaking at 200 rpm for 3 days, the primary cultures were transferred into 45 flasks of 500 mL capacity each containing the production medium composed of 0.3 g of peptone, 100 g of rice, and 100 mL of water. Still cultures were maintained at 18 °C for 30 days. Extraction of the cultures with EtOAc/MeOH (9:1, v/v, 3 × 13.5 L) furnished a crude extract. The crude extract (93 g) was fractioned by C_18_ RP-MPLC (59 × 7.2 cm, 1500 g), successively eluting with 10%, 30%, 50%, 70%, 90% MeOH in water and 100% MeOH, and separated into nine fractions (F_1_-F_9_). Fraction F_6_ (2.9 g, eluted from MPLC by 70% MeOH) was submitted to gel chromatography on a Sephadex LH-20 column, using CH_2_Cl_2_/MeOH (1: 1, v/v) as the mobile phase, to obtain eight mixtures (F_6-1_-F_6-8_). Purification of the mixture F_6-5_ (247 mg) by RP-HPLC on a Capcell PAK C_18_ column (MGII 5 μm, 10 × 250 mm) with 37% MeCN/H_2_O containing 0.1% formic acid (FA) led to isolation of **1** (3.4 mg) and **5** (1.2 mg). The other fraction F_5_ (5.1 g) eluted by 70% MeOH was re-chromatographed by silica gel with a gradient mixture of CH_2_Cl_2_/MeOH from 100: 1 to 0: 1 to give five mixtures (F_5-1_-F_5-5_). The mixtures F_5-2_ (544 mg) and F_5-4_ (440 mg) were decolored by Sephadex LH-20 chromatography (CH_2_Cl_2_/MeOH 1: 1, v/v) and further purified on the same RP-HPLC column mentioned above. This led to isolation of **2** (2.7 mg) and **3** (5.1 mg) from F_5-4_ eluting by 33% MeCN/H_2_O and **6** (6.0 mg) from F_5-2_ by 35% MeCN/H_2_O. Fraction F_8_ (7.1 g, 90% MeOH-eluting) was firstly decolored by Sephadex LH-20 chromatography (CH_2_Cl_2_/MeOH 1: 1) and then fractioned on a silica gel column with a gradient mixture of CH_2_Cl_2_/MeOH (100: 1 ~ 0: 1), resulting in the separation of 13 mixtures (F_8-5–1_-F_8-5–13_). Finally, compound **4** (12.9 mg) was purified from the mixture F_8-5–9_ (362 mg) by semi-preparative RP-HPLC (Cosmosil MS-II C_18_, 10 × 250 mm) with 47% MeCN/H_2_O containing 0.1% FA.

*Tolypyrone A* (**1**): colorless needle crystal; mp 305.8−306.5 °C; $[\alpha]^{25}_{\text D}$ − 254.3 (*c* 0.28, MeOH); UV (MeOH) λ_max_ (log *ε*) 241 (4.62), 279 (4.22) nm; ECD (*c* 2.95 × 10^−4^ M, MeOH) λ_max_ (Δ*ε*) 207 (−14.11), 240 (− 22.05), 298 (− 3.16) nm; IR ν_max_ 3280, 2952, 2926, 1693, 1614, 1454, 1377, 1210, and 1140 cm^−1^; ^1^H NMR (DMSO-*d*_6_ and CD_3_OD, 600 MHz), Tables 1 and S2 in the Supplementary Information; ^13^C NMR (DMSO-*d*_6_ and CD_3_OD, 150 MHz), Tables 1 and S2; HRESIMS *m/z* 340.1925 [M + H]^+^ (calcd 340.1913 for C_21_H_26_NO_3_).

*Tolypyroside A* (**2**): white, amorphous powder; $[\alpha]^{25}_{\text D}$ + 10.0 (*c* 0.12, MeOH); UV (MeOH) λ_max_ (log *ε*) 210 (4.03), 247 (3.86) nm; ECD (*c* 1.06 × 10^−3^ M, MeOH) λ_max_ (Δ*ε*) 210 (− 2.70), 246 (+ 0.96), 266 (− 0.39) nm; IR ν_max_ 3299, 2946, 2925, 1641, 1511, 1437, 1237, and 1046 cm^−1^; ^1^H NMR (CD_3_OD and DMSO-*d*_6_, 600 MHz), Tables 1 and S3; ^13^C NMR (CD_3_OD and DMSO-*d*_6_, 150 MHz), Tables 1 and S3; HRESIMS *m/z* 472.2319 [M + H]^+^ (calcd 472.2335 for C_26_H_34_NO_7_).

*Tolypyroside B* (**3**): white, amorphous powder; $[\alpha]^{25}_{\text D}$ − 19.2 (*c* 0.25, MeOH); UV (MeOH) λ_max_ (log *ε*) 214 (4.29), 250 (3.98), 287 (3.75) nm; ECD (*c* 1.03 × 10^−3^ M, MeOH) λ_max_ (Δ*ε*) 217 (− 12.59), 248 (+ 1.85), 308 (− 0.78) nm; IR ν_max_ 3272, 2949, 2923, 1641, 1616, 1511, 1440, 1237, and 1048 cm^−1^; ^1^H NMR (DMSO-*d*_6_, 600 MHz), Table 1; ^13^C NMR (DMSO-*d*_6_, 150 MHz), Table 1; HRESIMS *m/z* 488.2291 [M + H]^+^ (calcd 488.2284 for C_26_H_34_NO_8_).

*2′-Epitolypoalbin* (**4**): white, amorphous powder; $[\alpha]^{25}_{\text D}$ + 156.2 (*c* 0.85, MeOH); UV (MeOH) λ_max_ (log *ε*) 223 (4.12), 280 (4.16) nm; ECD (*c* 5.61 × 10^−4^ M, MeOH) λ_max_ (Δε) 217 (− 2.13), 231 (+ 12.27), 291 (+ 3.86) nm; IR ν_max_ 3293, 2965, 2923, 1657, 1608, 1516, 1451, 1336, 1243 and 968 cm^−1^; ^1^H NMR (CDCl_3_ and DMSO-*d*_6_, 600 MHz), Tables 2, S4 and S5; ^13^C NMR (CDCl_3_ and DMSO-*d*_6_, 150 MHz), Tables 2, S4 and S5; HRESIMS *m/z* 358.2009 [M + H]^+^ (calcd 358.2018 for C_21_H_28_NO_4_).

*Tolypyridone K2* (**5**): white, amorphous powder; $[\alpha]^{20}_{\text D}$ − 6.0 (*c* 0.80, MeOH); UV (MeOH) λ_max_ (log *ε*) 210 (4.01), 252 (3.76) nm; ECD (*c* 5.90 × 10^−4^ M, MeOH) λ_max_ (Δε) 200 (− 0.56), 263 (− 0.21) nm; IR ν_max_ 3242, 2954, 2926, 1611, 1516, 1444, 1378, 1212 cm^−1^; ^1^H NMR (DMSO-*d*_6_ and CD_3_OD, 600 MHz), Tables 2 and S6; ^13^C NMR (DMSO-*d*_6_ and CD_3_OD, 150 MHz), see Tables 2 and S6; HRESIMS *m/z* 340.1925 [M + H]^+^ (calcd 340.1913 for C_21_H_26_NO_3_).

*Tolypyridone L2* (**6**): white, amorphous powder; $[\alpha]^{20}_{\text D}$ + 9.5 (*c* 0.80, MeOH); UV (MeOH) λ_max_ (log *ε*) 210 (4.72), 254 (4.49) nm; ECD (*c* 5.90 × 10^−4^ M, MeOH) λ_max_ (Δ*ε*) 215 (− 0.94), 228 (+ 0.54), 266 (− 0.20) nm; IR ν_max_ 3212, 2956, 1641, 1614, 1516, 1448, 1270, 1223, and 836 cm^−1^; ^1^H NMR (DMSO-*d*_6_ and CD_3_OD, 600 MHz), Tables 2 and S6; ^13^C NMR (DMSO-*d*_6_ and CD_3_OD, 150 MHz), see Tables 2 and S6; HRESIMS *m/z* 340.1917 [M + H]^+^ (calcd 340.1913 for C_21_H_26_NO_3_).

### Crystallographic analysis of 1

The crystals of **1** were yielded by re-crystallization in MeOH. A single crystal (0.25 × 0.03 × 0.02) was selected for crystallographic analysis on a Rigaku XtaLAB Synergy diffractometer with Cu Kα radiation (λ = 1.54184 Å). Crystal data of **1**: colorless needle crystal, C_21_H_25_NO_3_, *M* = 339.42, orthorhombic, space group P2_1_2_1_2_1_ (no. 19); a = 8.6674(14) Å, b = 10.2984(16) Å, c = 20.3542(3) Å, *α* = 90°, *β* = 90°, *γ* = 90°, *V* = 1816.82(5) Å^3^, *Z* = 4, *Dx* = 1.241 g/cm^3^, *F (000)* = 728.0, *μ* (Cu *K*α) = 0.658 mm^−1^. 17,480 measured reflections (8.688° ≤ 2θ ≤ 154.328°), 3726 independent unique reflections (R_int_ = 0.0507, R_sigma_ = 0.0325), *R*_*1*_ = 0.0361, *wR*_2_ = 0.0934, *S* = 1.055, Flack parameter =  − 0.06(9). The crystallographic data is accessible through the Cambridge Crystallographic Data Centre (CCDC 2499034, https://www.ccdc.cam.ac.uk).

### Advanced Marfey’s analysis

A sample of **4** or **9** (1 mg) was dissolved in 1 M HCl/EtOH (500 μL) and refluxed at 110 °C for 15 min. After evaporation to dryness, the residue was re-dissolved in 0.5 M KOH (500 μL) and stirred at rt for 2 h. The resultant hydrolysate was applied to advanced Marfey’s analysis using a protocol described previously [15]. The details were given in the Supplementary Information.

### Determination of absolute configuration of the ribose moiety in 2 and 3.

Compound **2** or **3** (0.5 mg) was treated with 2 M HCl (1 mL) and heated at 110 ℃ for 4 h. The hydrolyzed products were concentrated *in vacuo* to dryness and then re-suspended in acetic acid (2 mL) and added 2,3-naphthalenediamine (2 mg) and molecular iodine (0.5 mg). The mixtures was agitated at rt for 6 h to afford the fluorescent naphthimidazole derivative [27]. The derivatives of l- and d-ribose authentic standards were similarly prepared. The derivatized materials were applied to LC–MS analysis on a chiral column (Capcell CD-ph 5 μm, 10 × 250 mm; 18% MeCN with 0.1% FA, 2 mL/min; column temperature: 35 °C). The standard l- and d-ribose-naphthimidazole derivatives gave retention times at 10.91 and 17.13 min, respectively, while the derivatives of** 2** and** 3** were eluted at 17.19 and 17.13 min (Figure S3). Thus, the ribose moiety in **2** and** 3** was determined to be of d-configuration.

### ECD calculation

See the Supplementary Information.

### Antimicrobial assay

Antimicrobial assay was performed against *Staphylococcus aureus* R6101, *S. aureus* ATCC 29213, *S. epidermidis* ATCC 12228, *Bacillus subtilis* ATCC 6633, *Escherichia coli* ATCC 25922, *Pseudomonas aeruginosa* ATCC 27853*, Edwardsiella ictaluri* ATCC 33202*, E. tarda* QDIO-2, *Candida albicans* ATCC 10231, *Mucor racemosus* CICC 3112, and *Magnaporthe oryzae* 131 according to the procedure described previously [35].

### Cytotoxicity assay

Cytotoxicity assay was conducted in accordance with the protocol described previously [36].

## Supplementary Information

Additional file1 (PDF 7538 kb)

## Data Availability

The data generated in this study were included in the article and its supplementary materials.
