# Supplementary material for: Pyridone alkaloids from an Antarctic endolichenic Tolypocladium sp
Source: Nat Prod Bioprospect. 2026 Apr 1;16(1):46. doi: 10.1007/s13659-026-00607-1 (PMC13043937; doi:10.1007/s13659-026-00607-1)
Supplement: Supplementary file 1 — Additional file1 (PDF 7538 kb) [file 13659_2026_607_MOESM1_ESM.pdf]

Supporting Information

Pyridone Alkaloids from an Antarctic Endolichenic *Tolypocladium* sp.

*Shasha Li,[1] Ting Yu,[1] Jianju Feng,[2] Yue Shang,[1] Tao Zhang,[2] Shuzhen Chen[1] Liyan Yu,[2] Maoluo Gan[1,]**

[1]Beijing Key Laboratory of Technology and Application for Anti-Infective New Drugs Research and Development, Institute of Medicinal Biotechnology, Chinese Academy of Medical Sciences and Peking Union Medical College, Beijing 100050, China

[2]China Pharmaceutical Culture Collection, Institute of Medicinal Biotechnology, Chinese Academy of Medical Sciences and Peking Union Medical College, Beijing 100050, China

*Correspondence, e-mail addresses: ganml@imb.pumc.edu.cn

Table of Conents

Experimental Section 4
Table S1. 4-Hydroxy-2-Pyridones Isolated from *Tolypocladium* sp. 7
Figure S1. Structures of 4-hydroxy-2-pyridones isolated from *Tolypocladium* sp. 8
Table S2. Comparison of ^{1}H (600 MHz) and ^{13}C NMR (150 MHz) Data for Compound **1** and Reported for Tolypyridinol A 9
Table S3. Comparison of ^{1}H (600 MHz) and ^{13}C NMR (150 MHz) Data for Compounds **2** and **7** 10
Table S4. Comparison of ^{1}H and ^{13}C NMR Data for Compounds **4** and Reported for Tolypoalbin (**9**) . 11
Table S5. Comparison of ^{1}H (500 MHz) and ^{13}C NMR (125 MHz) Data in DMSO-d_6 for Compounds **4** and **9** 12
Table S6. Comparison of ^{1}H and ^{13}C NMR Data for Compounds **5**, **6** and Reported for Tolypyridones K and L in CD_3OD 13
Figure S2. The Marfey's analysis of the hydrolysates of compounds **4** and **9**. Extracted ion chromatogram at *m/z* 476.18 for the FDLA derivative of Tyr. 14
Figure S3. Chromatographic determination of the absolute configuration of the ribose residue in **2**, **3** and **7** by naphthimidazole derivatization.. 15
Figure S4. Graphical comparison of the tolypyridone C biosynthetic gene clusters from *Tolypocladium* sp. CPCC401485 and *Tolypocladium* sp. 49Y 16
Figure S5. Proposed biosynthetic pathway of the compounds **1–14** 17
Figure S6. The key COSY, HMBC and ROESY correlations of compounds **5** and **6** 17
Figure S7. The phylogenetic tree for. *Tolypocladium* sp. CPCC401485 ITS sequence. 18
Figure S8. The experimental ECD and UV spectrum for compounds **1–3** in MeOH 19
Figure S9. The experimental ECD and UV spectrum for compounds **4–6** in MeOH 20
Figure S10. The (+)-HRESIMS spectrum of compound **1** 21
Figure S11. The IR spectrum of compound **1**. 22
Figure S12. The ^{1}H NMR spectrum of compound **1** in DMSO-d_6 (600 MHz) 23
Figure S13. The ^{13}C NMR spectrum of compound **1** in DMSO-d_6 (150 MHz) 24
Figure S14. The ^{1}H-^{1}H COSY spectrum of compound **1** in DMSO-d_6 (600 MHz) 25
Figure S15. The HSQC spectrum of compound **1** in DMSO-d_6 (600 MHz). 26
Figure S16. The HMBC spectrum of compound **1** in DMSO-d_6 (600 MHz). 27
Figure S17. The ROESY spectrum of compound **1** in DMSO-d_6 (600 MHz) 28
Figure S18. The ^{1}H NMR spectrum of compound **1** in CD_3OD (600 MHz). 29
Figure S19. The ^{13}C NMR spectrum of compound **1** in CD_3OD (150 MHz). 30
Figure S20. The HSQC spectrum of compound **1** in CD_3OD (600 MHz) 31
Figure S21. The HMBC spectrum of compound **1** in CD_3OD (600 MHz) 32
Figure S22. The (+)-HRESIMS spectrum of compound **2** 33
Figure S23. The IR spectrum of compound **2**. 34
Figure S24. The ^{1}H NMR spectrum of compound **2** in CD_3OD (600 MHz). 35
Figure S25. The ^{13}C NMR spectrum of compound **2** in CD_3OD (150 MHz). 36
Figure S26. The DEPT spectrum of compound **2** in CD_3OD (150 MHz) 37
Figure S27. The ^{1}H-^{1}H COSY spectrum of compound **2** in CD_3OD (600 MHz). 38
Figure S28. The HSQC spectrum of compound **2** in CD_3OD (600 MHz) 39
Figure S29. The HMBC spectrum of compound **2** in CD_3OD (600 MHz) 40
Figure S30. The ROESY spectrum of compound **2** in CD_3OD (600 MHz). 41
Figure S31. The (+)-HRESIMS spectrum of compound **3** 42
Figure S32. The IR spectrum of compound **3**. 43
Figure S33. The ^{1}H NMR spectrum of compound **3** in DMSO-d_6 (600 MHz) 44
Figure S34. The ^{13}C NMR spectrum of compound **3** in DMSO-d_6 (150 MHz) 45
Figure S35. The ^{1}H-^{1}H COSY spectrum of compound **3** in DMSO-d_6 (600 MHz) 46
Figure S36. The TOCSY spectrum of compound **3** in DMSO-d_6 (600 MHz) 47
Figure S37. The HSQC spectrum of compound **3** in DMSO-d_6 (600 MHz). 48
Figure S38. The HMBC spectrum of compound **3** in DMSO-d_6 (600 MHz). 49
Figure S39. The ROESY spectrum of compound **3** in DMSO-d_6 (600 MHz) 50
Figure S40. The (+)-HRESIMS spectrum of compound **4** 51
Figure S41. The IR spectrum of compound **4**. 52
Figure S42. The ^{1}H NMR spectrum of compound **4** in $CDCl_3$ (600 MHz). 53
Figure S43. The ^{13}C NMR spectrum of compound **4** in $CDCl_3$ (150 MHz). 54
Figure S44. The ^{1}H-^{1}H COSY spectrum of compound **4** in $CDCl_3$ (600 MHz). 55
Figure S45. The HSQC spectrum of compound **4** in $CDCl_3$ (600 MHz). 56

Figure S46. The HMBC spectrum of compound **4** in $CDCl_3$ (600 MHz). 57
Figure S47. The (+)-HRESIMS spectrum of compound **5**. 58
Figure S48. The IR spectrum of compound **5**. 59
Figure S49. The ^1H NMR spectrum of compound **5** in DMSO-d_6 (600 MHz). 60
Figure S50. The ^{13}C NMR spectrum of compound **5** in DMSO-d_6 (150 MHz). 61
Figure S51. The HSQC spectrum of compound **5** in DMSO-d_6 (600 MHz). 62
Figure S52. The HMBC spectrum of compound **5** in DMSO-d_6 (600 MHz). 63
Figure S53. The ROESY spectrum of compound **5** in DMSO-d_6 (600 MHz). 64
Figure S54. The ^1H NMR spectrum of compound **5** in CD_3OD (600 MHz). 65
Figure S55. The ^{13}C NMR spectrum of compound **5** in CD_3OD (150 MHz). 66
Figure S56. The (+)-HRESIMS spectrum of compound **6**. 67
Figure S57. The IR spectrum of compound **6**. 68
Figure S58. The ^1H NMR spectrum of compound **6** in DMSO-d_6 (600 MHz). 69
Figure S59. The ^{13}C NMR spectrum of compound **6** in DMSO-d_6 (150 MHz). 70
Figure S60. The DEPT spectrum of compound **6** in DMSO-d_6 (150 MHz). 71
Figure S61. The ^1H-^1H COSY spectrum of compound **6** in DMSO-d_6 (600 MHz). 72
Figure S62. The HSQC spectrum of compound **6** in DMSO-d_6 (600 MHz). 73
Figure S63. The HMBC spectrum of compound **6** in DMSO-d_6 (600 MHz). 74
Figure S64. The ROESY spectrum of compound **6** in DMSO-d_6 (600 MHz). 75
Figure S65. The ^1H NMR spectrum of compound **6** in CD_3OD (600 MHz). 76
Figure S66. The ^{13}C NMR spectrum of compound **6** in CD_3OD (150 MHz) 77
Figure S67. The LC-MS analysis of compounds **4** and **9** 78
References 79

Experimental Section

General experimental procedures

Melting point was measured on a Mettler Toledo MP90 melting point system. The specific rotations were measured on a Perkin-Elmer model 343 polarimeter. ECD spectra were measured by an Applied Photophysics Chirascan spectrometer. IR spectra were obtained on a Nicolet 5700 FT-IR microscope spectrometer using the FT-IR microscope transmission method. 1D- and 2D-NMR spectra were acquired at 600 MHz for ^{1}H and 150 MHz for ^{13}C, respectively, on a Bruker AVANCE III HD 600 MHz spectrometers using the solvent peak (DMSO-d_6: δ_H 2.50 and δ_C 39.52; $CDCl_3$: δ_H 7.26 and δ_C 77.16; CD_3OD: δ_H 3.31 and δ_C 49.00) as a reference. The crystal data were obtained at 293 K using Cu Kα radiation (1.54184 Å) on a Rigaku Oxford Diffraction XtaLAB Synergy four-circle diffractometer. LC-HRESIMS analysis were carried on Waters LC H-class/Xevo G2-XS Q-TOF mass spectrometers. MPLC was performed on a Buchi C-850 Pure Chromatography System. Column chromatography was carried out using Sephadex LH-20 (GE Healthcare Bio-Science AB), Silica gel (Qingdao Marine Chemical Factory, China). Preparative HPLC separation was performed with a Shimadzu LC-20AP binary pump equipped with an SPD-M20A diode array detector.

Advanced Marfey's Analysis

A solution of **4** or **9** (1 mg) in 1 M HCl/EtOH (500 μL) was heated at 110 °C for 15 min and then evaporated to dryness. The mixture was dissolved in 0.5 M KOH (500 μL) and stirred for 2 h at room temperature, which was then neutralized with 125 μL of HCl. The hydrolysate was concentrated to dryness *in vacuo* and redissolved in 120 μL H_2O. The resulting solution was divided into two equal portions. Each portion (60 μL) was added to 1M $NaHCO_3$ (20 μL) and L- or D-FDLA (1% in acetone, 100 μL), respectively, and incubated at 40 °C for 1 h. After cooling to room temperature, the mixtures were quenched by addition of 10 μL of 2 M HCl and dried *in vacuo*. The residue was diluted with MeCN (100

μL) and filtered prior to LC-MS analysis. The L- and D-FDLA derivatives of the standard amino acids (L-Tyr) were prepared as above. Aliquots of (1 μL) of analytes were subjected to LC-MS analysis (Waters Acquity BEH C_{18} 1.7 μm, 2.1 × 100 mm, flow rate, 0.3 mL/min, a linear gradient of 20% to 50% MeCN containing 0.1% formic acid over 15 min, 15-17 min, 50-100% MeCN, column temperature 40 ℃, UV detection at 340 nm).

ECD Calculation

The conformational distributions for (3*R*, 4*S*, 6*R*, 8*R*, 9*S*)-**1**, (2′*R*, 4*S*, 6*S*)-**4**, and the truncated model compounds (3*R*, 4*S*, 6*R*, 8*S*, 9*R*)-**2a**, (3*R*, 4*S*, 6*R*, 8*R*, 9*S*)-**3a**, were searched by MOE2019 software [1] using the LowModeMD method. Conformers within 5.0 kcal/mol were subjected to optimization at the B3LYP/6-31+G (d, p) level in gas using Gaussian 16 [2]. The optimized conformers within the 2.0 kcal/mol energy were used for ECD calculation using the TDDFT methodology (NStates = 60) at the CAM-B3LYP/6-311+G (d, p) level for **1** and **2** and B3LYP/6-311+G (d, p) level for **3** and **4** with the PCM model for MeOH. Final ECD spectra were generated using SpecDis software [3] (σ = 0.30 eV) by computing the average of the conformers data based on their Boltzmann distribution.

Antimicrobial Assay

Antimicrobial activities were evaluated against Gram-positive bacteria *Staphylococcus aureus* ATCC 29213, methicillin-resistant *S. aureus* (MRSA) R6101, *S. epidermidis* ATCC 12228, and *Bacillus subtilis* ATCC 6633, Gram-negative *Escherichia coli* ATCC 25922 and *Pseudomonas aeruginosa* ATCC 27853*,* aquatic pathogenic bacteria *Edwardsiella tarda* QDIO-2 and *E. ictaluri* ATCC33202, as well as the human pathogenic fungus *Candida albicans* ATCC 10231 and plant-pathogenic fungi *Mucor racemosus* CICC 3112, *Magnaporthe oryzae* 131. The minimum inhibitory concentrations (MICs) were determined in 96-well plates by using the micro-dilution method in Mueller-Hinton medium (0.6% beef extract, 1.75% acid hydrolysate of casein, 0.15% starch) for bacteria and *C. albicans* and PDB medium

for plant-pathogenic fungi as described previously [4]. Rifampicin and amphotericin B were used as positive control. MICs values were determined as the lowest concentration without the visible growth of the microbes.

Cytotoxicity assay

Cytotoxicity was evaluated by using the Cell Counting Kit-8 (CCK-8, Meilunbio, China) staining method as described previously [5]. H460, and HCT116 cells from ATCC were seeded at 2.5×10^3 and 4.5×10^3 cells/well into the 96-well plate, respectively. Overnight, the cells were exposed to different concentration of compounds for another 48 h. Then, the CCK-8 reagent (10 μL/well) was added to the cells for 2 h. Absorbance at 450 nm was recorded with a microplate reader (ThermoFisher Scientific, USA). The cell survival was analyzed according to the absorbance of untreated cells. The experiments were performed in triplicate.

Table S1. 4-Hydroxy-2-Pyridones Isolated from *Tolypocladium* sp.

Compound	Source of isolation	Suggested name[d]	Year	References
Tolypyridone A	*Tolypocladium cylindrosporum*		2015	[6]
Trichodin A	*Trichoderma* sp. Strain MF106		2014	[7]
Tolypyridone B	*Tolypocladium cylindrosporum*		2015	[6, 8]
Pyridoxatin	*Tolypocladium cylindrosporum*		2015	[6]
Tolypyridone C	*Tolypocladium* sp. 49Y		2020	[9]
Tolypyridone D	*Tolypocladium* sp. 49Y		2020	[9]
Tolypyridone E	*Tolypocladium* sp. 49Y		2020	[9]
Tolypyridone F	*Tolypocladium* sp. 49Y		2020	[9]
Tolypyridone G	*Tolypocladium* sp. 49Y		2020	[9]
Tolypyridone H	*Tolypocladium* sp. 49Y		2020	[9]
Tolypyridone I [a]	*Tolypocladium album* dws120	Tolypyridone I1	2023	[10]
Tolypyridone I [b,c]	*Tolypocladium cylindrosporum* FB06	Tolypyridone L1	2024	[11]
Tolypyridone J [a]	*Tolypocladium album* dws120	Tolypyridone J1	2023	[10]
Tolypyridone J [b]	*Tolypocladium cylindrosporum* FB06	Tolypyridone J2	2024	[11]
Tolypyridone K [a]	*Tolypocladium album* dws120	Tolypyridone K1	2023	[10]
Tolypyridone K [b]	*Tolypocladium* sp. strain CNC14	Tolypyridone K2	2024	[12]
Tolypyridone L [a,c]	*Tolypocladium album* dws120	Tolypyridone L1	2023	[10]
Tolypyridone L [b]	*Tolypocladium* sp. strain CNC14	Tolypyridone L2	2024	[12]
Tolypyridone M	*Tolypocladium album* dws120		2023	[10]
Tolypyridinol A	*Tolypocladium* sp. CNC14		2024	[12]
Tolypyridinol B	*Tolypocladium* sp. CNC14		2024	[12]

[a] The trivial name was firstly reported in the literature. [b] The duplicate name was reported later in the literature.[c] The structures of these compounds are identical. [d] The compounds were suggested to be renamed with a suffix number according to the discovery time sequence, wherein the numbers 1 and 2 indicated a compound discovered earlier and later, respectively.

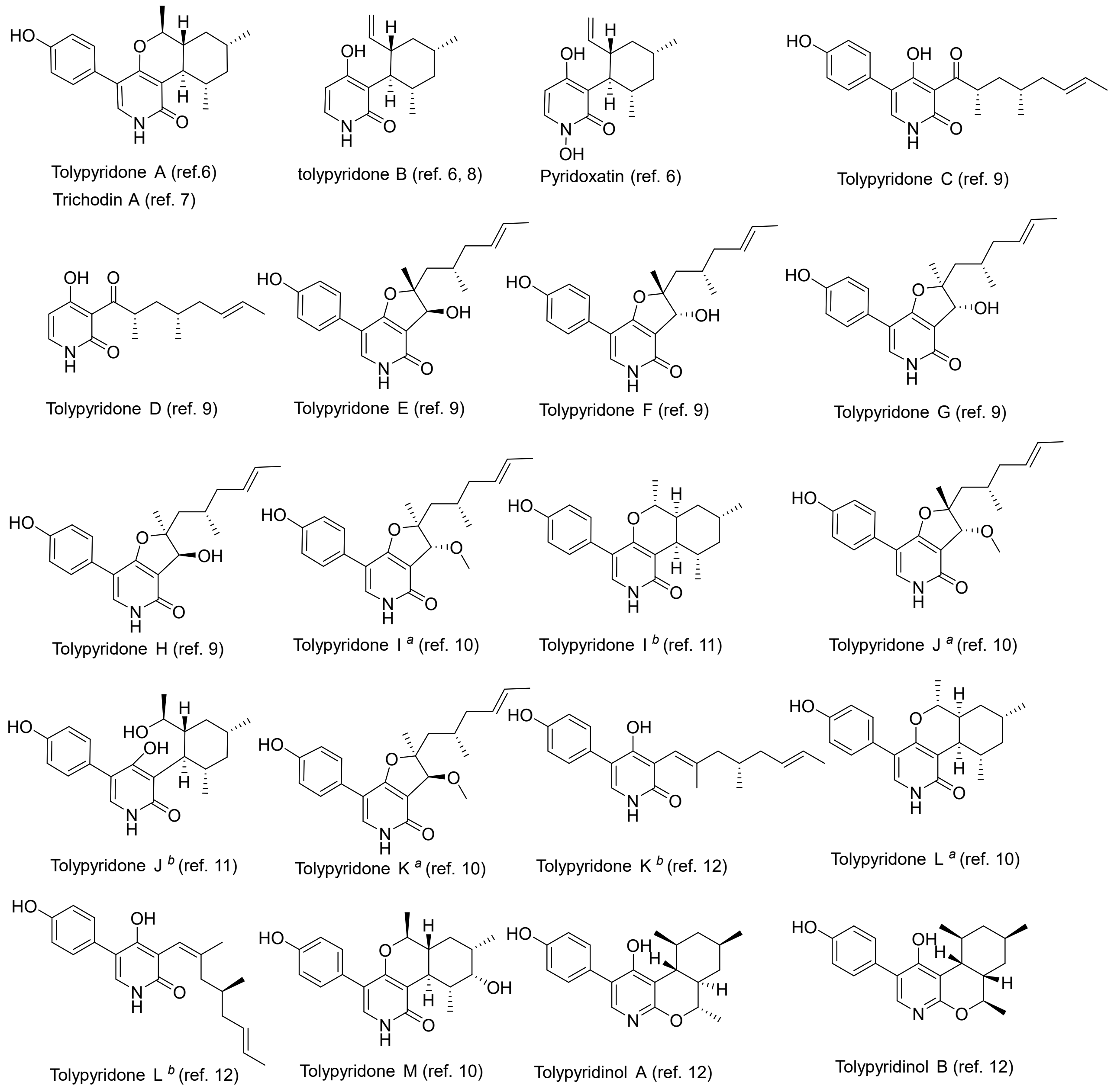

Figure S1. Structures of 4-hydroxy-2-pyridones isolated from *Tolypocladium* sp.

Table S2. Comparison of ^{1}H (600 MHz) and ^{13}C NMR (150 MHz) Data for Compound **1** and Reported for Tolypyridinol A

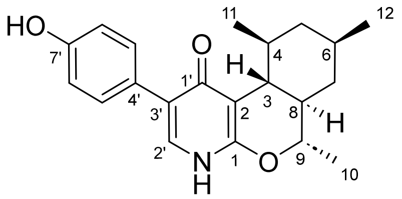

1

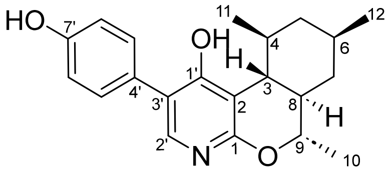

Tolypyridinol A

no.	**1** (DMSO-d_6) ^{a}		**1** (CD_3OD) ^{a}		Tolypyridinol A (CD_3OD) ^{b}	
	δ_C, type	δ_H, mult. (J in Hz)	δ_C, type	δ_H, mult. (J in Hz)	δ_C, type	δ_H, mult. (J in Hz)
1	155.2, C		158.3, C		159.0, C	
2	107.3, C		109.9, C		111.1, C	
3	43.0, CH	2.26, t (10.2)	44.8, CH	2.40, t (10.2)	44.9, CH	2.47, t (9.9)
4	41.1, CH	1.44, m	42.9, CH	1.58, m	42.0, CH	1.71, m
5α	45.8, CH_2	0.96, m	47.0, CH_2	1.08, ddd (13.2, 12.0, 12.0)	46.9, CH_2	1.08, dt (13.1, 11.8)
5β		1.66, brd (13.2)		1.76, brd (13.2)		1.81, brd (13.1)
6	32.6, CH	1.62, m	34.4, CH		34.2, CH	
7α	36.6, CH_2	0.83, dt (12.0, 10.2)	38.1, CH_2	0.90, dt (12.0, 10.2)	38.1, CH_2	0.92, dt (12.3, 11.9)
7β		1.76, brd (10.2)		1.87, brd (13.2)		1.90, brd (12.3)
8	49.4, CH	1.42, qd (10.2, 2.4)	51.3, CH	1.53, qd (10.2, 2.4)	50.6, CH	1.63, m
9	78.4, CH	3.78, m	**80.5, CH**	3.83, dq (10.2, 6.0)	**82.2, CH**	4.01, m
10	18.7, CH_3	1.29, d (6.0)	19.0, CH_3	1.38, d (6.0)	19.2, CH_3	1.43, d (4.7)
11	23.1, CH_3	0.97, d (6.6)	23.4, CH_3	1.09, d (7.2)	23.4, CH_3	1.16, d (6.8)
12	22.5, CH_3	0.92, d (6.0)	22.8, CH_3	0.99, d (6.6)	22.8, CH_3	0.85, d (6.5)
1′	178.1, C		**179.9, C**		**175.2, C**	
2′	129.0, CH	7.19, s	**131.9, CH**	**7.27, s**	**134.1, CH**	**7.55, s**
3′	124.3, C		127.1, C		126.2, C	
4′	127.1, C		126.6, C		125.9, C	
5′	129.5, CH	7.33, d (7.8)	131.2, CH	7.28, d (8.4)	131.8, CH	7.42, brd (8.4)
6′	114.7, CH	6.70, d (7.8)	116.0, CH	6.79, d (8.4)	116.8, CH	6.84, brd (8.4)
7′	156.1, C		**157.8, C**		**159.7, C**	
8′	114.7, CH	6.70, d (7.8)	116.0, CH	6.79, d (8.4)	116.8, CH	6.84, brd (8.4)
9′	129.5, CH	7.33, d (7.8)	131.2, CH	7.28, d (8.4)	131.8, CH	7.42, brd (8.4)
NH		11.36, brs				
OH		9.35, brs				

^{a} The assignments were made by 2D NMR (COSY, HSQC, HMBC and ROESY) data. ^{b} Reported data in CD_3OD from ref. [12]

Table S3. Comparison of ^{1}H (600 MHz) and ^{13}C NMR (150 MHz) Data for Compounds **2** and **7**

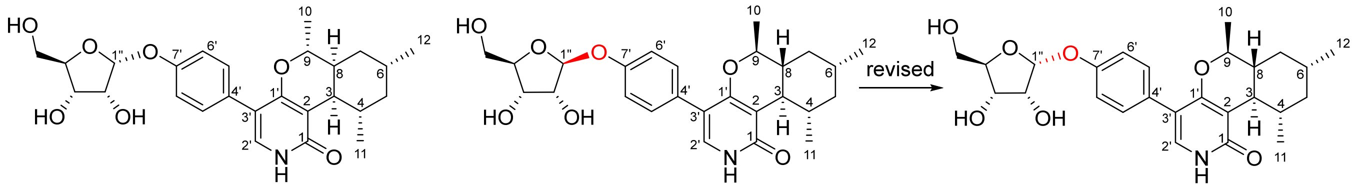

2 Trichodin (**7**) revised structure of **7**

no.	**2** (DMSO-d_6) [a]		**2** (CD_3OD) [a]		**7** (DMSO-d_6) [b]		**7** (CD_3OD) [c]	
	δ_C, type	δ_H, mult. (J in Hz)	δ_C, type	δ_H, mult. (J in Hz)	δ_C, type	δ_H, mult. (J in Hz)	δ_C, type	δ_H, mult. (J in Hz)
1	162.6, C		165.4, C		162.7, C		165.8, C	
2	110.3, C		112.3, C		111.5, C		113.0, C	
3	**36.6, CH**	**2.46, dd (10.8, 3.6)**	**38.6, CH**	**2.60, dd (10.8, 4.2)**	**43.7, CH**	**2.11, t (10.2)**	**45.3, CH**	**2.26, t (10.1)**
4	35.9, CH	1.55, m	37.7, CH	1.66, m	49.0, CH	1.40, m	50.9, CH	1.53, ddd (24.0, 10.2, 2.6)
5α	44.3, CH_2	0.82, q (12.0)	45.7, CH_2	0.91, overlap	37.1, CH_2	0.78, q (12.0)	38.5, CH_2	0.89, dd (24.0, 12.0)
5β		1.63, brd (12.0)		1.73, brd (13.8)		1.74, brd (13.2)		1.87, brd (12.2)
6	26.2, CH	1.56, m	28.0, CH	1.67, m	32.4, CH	1.58, m	34.1, CH	1.68, m
7α	35.4, CH_2	1.26, overlap	37.0, CH_2	1.36, ddd (13.8, 13.2, 4.2)	45.8, CH_2	0.92, q (12.0)	47.1, CH_2	1.06, dd (24.7, 11.8)
7β		1.77, brd (13.8)		1.88, brd (13.8)		1.67, brd (13.2)		1.78, brd (13.0)
8	38.1, CH	1.58, m	40.1, CH	1.70, m	39.8, CH	1.62, m	41.9, CH	1.71, m
9	**72.2, CH**	**4.56, dq (12.0, 6.0)**	**74.6, CH**	**4.68, dq (12.0, 6.0)**	**78.0, CH**	**3.47, m**	**79.7, CH**	**3.68, m**
10	19.7, CH_3	1.26, d (6.0)	20.1, CH_3	1.33, d (6.0)	19.1, CH_3	1.19, d (6.0)	19.1, CH_3	1.28, d (6.2)
11	20.6, CH_3	0.86, d (6.6)	21.0, CH_3	0.96, d (6.6)	23.1, CH_3	1.01, d (6.6)	23.2, CH_3	1.12, d (6.7)
12	22.7, CH_3	0.85, d (6.0)	23.2, CH_3	0.92, d (6.6)	22.6, CH_3	0.90, d (6.6)	22.8, CH_3	0.99, d (6.4)
1′	**158.8, C**		**162.3, C**		**163.3, C**		**166.0, C**	
2′	131.2, CH	7.08, s	132.2, CH	7.16, s	131.1, CH	7.09, s	131.6, CH	7.15, s
3′	112.1, C		116.8, C		113.3, C		117.2, C	
4′	127.9, C		129.5, C		127.9, C		129.3, C	
5′	129.8, CH	7.31, d (9.0)	131.2, CH	7.33, d (8.4)	130.0, CH	7.29, d (8.4)	131.2, CH	7.33, d (8.7)
6′	116.3, CH	7.01, d (9.0)	117.8, CH	7.14, d (8.4)	116.6, CH	7.01, d (8.4)	117.9, CH	7.13, d (8.7)
7′	156.0, C		158.1, C		156.2, C		158.1, C	
8′	116.3, CH	7.01, d (9.0)	117.8, CH	7.14, d (8.4)	116.6, CH	7.01, d (8.4)	117.9, CH	7.13, d (8.7)
9′	129.8, CH	7.31, d (9.0)	131.2, CH	7.33, d (8.4)	130.0, CH	7.29, d (8.4)	131.2, CH	7.33, d (8.7)
1″	100.4, CH	5.57, d (4.2)	102.4, CH	5.65, d (4.2)	100.6, CH	5.57, d (4.8)	102.4, CH	5.64, d (4.4)
2″	71.5, CH	4.06, dd (6.0, 4.2)	73.4, CH	4.20, dd (6.6, 4.2)	71.7, CH	3.96, dd (7.2, 2.4)	73.4, CH	4.19, dd (6.4, 4.5)
3″	69.3, CH	3.92, dd (6.0, 3.6)	71.2, CH	4.10, dd (6.6, 3.0)	69.6, CH	3.92, dd (6.6, 3.6)	71.2, CH	4.10, dd (6.5, 3.2)
4″	86.1, CH	3.96, dt (3.6, 4.2)	87.5, CH	4.15, dt (4.2, 3.0)	86.4, CH	4.06, brs	87.5, CH	5.15, dd (7.0, 3.5)
5″	61.5, CH_2	3.48, dd (12.0, 4.2)	63.2, CH_2	3.71, dd (12.0, 4.2)	61.8, CH_2	3.48, dd (12.0, 4.2)	63.2, CH_2	3.71, dd (12.1, 3.4
		3.45, dd (12.0, 4.2)		3.66, dd (12.0, 4.2)		3.45, dd (12.0, 4.2)		3.65, dd (12.1, 4.0)
NH		11.10, s				11.00, s		
OH								

[a] The assignments were made by 2D NMR (COSY, HSQC, HMBC and ROESY) data. [b] Recorded in DMSO-d_6 in this study. [c] ^{1}H (600 MHz) and ^{13}C NMR (125 MHz) data in CD_3OD reported from ref. [7]

Table S4. Comparison of ^{1}H and ^{13}C NMR Data for Compounds **4** and Reported for Tolypoalbin (**9**)

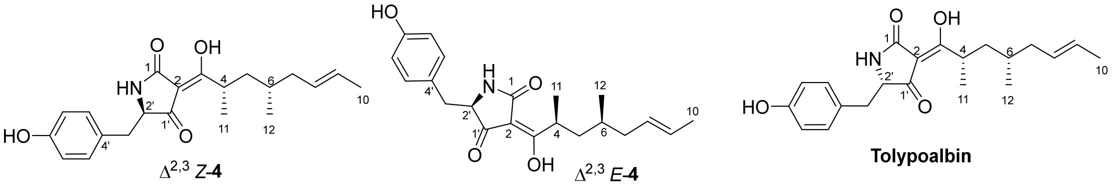

no.	2*Z*-**4** (CDCl$_3$)[a]		2*E*-**4** (minor) (CDCl$_3$)[a]		Tolypoalbin (CDCl$_3$)[b]	
	δ_C, type	δ_H, mult. (*J* in Hz)	δ_C, type	δ_H, mult. (*J* in Hz)	δ_C, type	δ_H, mult. (*J* in Hz)
1	175.5, C		168.9, C		175.3, C	
2	100.6, C		103.8, C		100.5, C	
3	193.7, C		201.5, C		194.0, C	
4	34.1, CH	3.78, m	34.2, CH	3.76, m	34.0, CH	3.80, m
5α	40.5, CH_2	1.79, m	40.5, CH_2	1.78, m	40.3, CH_2	1.81, m
5β		1.18, m		1.16, m		1.18, m
6	31.4, CH	1.34, m	31.5, CH	1.32, m	31.3, CH	1.38, m
7α	40.5, CH_2	1.97, m	40.4, CH_2	1.95, m	40.4, CH_2	1.98, m
7β		1.82, m		1.81, m		1.81, m
8	129.4, CH	5.36, dt (15.0, 7.2)	129.4, CH	5.35, dt (15.0, 7.2)	129.3, CH	5.38, m
9	126.6, CH	5.42, dq (15.0, 6.0)	126.6, CH	5.41, dq (15.0, 6.0)	126.4, CH	5.41, m
10	18.2, CH_3	1.66, d (6.0)	18.1, CH_3	1.64, d (6.0)	18.0, CH_3	1.64, d (5.5)
11	18.5, CH_3	1.19, d (6.6)	18.7, CH_3	1.16, d (6.6)	18.4, CH_3	1.17, d (6.5)
12	19.7, CH_3	0.87, d (6.6)	19.6, CH_3	0.86, d (6.6)	19.5, CH_3	0.87, d (7.0)
1′	194.1, C		196.6, C		193.6, C	
2′	63.6, CH	3.98, dd (9.0, 3.6)	60.7, CH	4.14, dd (9.0, 3.6)	63.5, CH	3.99, brd (8.0)
3′	37.3, CH_2	3.16, dd (13.8, 3.6)	37.5, CH_2	3.19, dd (14.4, 4.2)	37.4, CH_2	3.19, brd (14.0)
		2.68, dd (13.8, 9.6)		2.68, dd (13.8, 9.0)		2.66, dd (14.0, 8.0)
4′	127.9, C		127.4, C		128.0, C	
5′/9′	130.5, CH	7.03, d (7.8)	130.5, CH	7.03, d (7.8)	130.3, CH	7.03, brs
6′/8′	115.9, CH	6.74, d (7.8)	115.9, CH	6.75, d (7.8)	115.8, CH	6.77, brs
7′	155.1, C		155.4, C		155.0, C	
OH-7′						9.17, s
NH		5.99, brs		5.80, brs		

[a] ^{1}H (600 MHz) and ^{13}C NMR (150 MHz) data in CDCl$_3$ in this study, the assignments were made by 2D NMR (COSY, HSQC, HMBC and ROESY) data. [b] ^{1}H (500 MHz) and ^{13}C NMR (100 MHz) data in CDCl$_3$ reported from ref. [13]

Table S5. Comparison of ^{1}H (500 MHz) and ^{13}C NMR (125 MHz) Data in DMSO-d_6 for Compounds **4** and **9** [a]

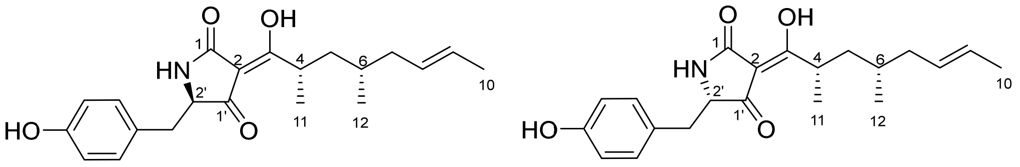

4 **9**

no.	**4** (DMSO-d_6)		**9** (DMSO-d_6)	
	δ_C, type	δ_H, mult. (J in Hz)	δ_C, type	δ_H, mult. (J in Hz)
1	175.2, C		175.4, C	
2	100.9, C		100.6, C	
3	194.9, C		194.9, C	
4	33.4, CH	3.64, brs	33.4, CH	3.60, brs
5α	39.8, CH_2	1.59, m	39.8, CH_2	1.64, m
5β		0.95, m		1.06, m
6	30.6, CH	1.11, m	30.8, CH	1.23, m
7α	40.0, CH_2	1.87, m	40.0, CH_2	1.87, m
7β		1.75, m		1.75, m
8	129.4, CH	5.31, m	129.4, CH	5.32, m
9	125.9, CH	5.37, m	125.9, CH	5.38, m
10	17.9, CH_3	1.62, d (5.5)	17.9, CH_3	1.60, d (5.0)
11	18.1, CH_3	1.01, d (7.0)	18.2, CH_3	0.95, d (7.0)
12	19.4, CH_3	0.80, d (6.5)	19.2, CH_3	0.77, d (6.5)
1′	191.8, C		191.8, C	
2′	62.0, CH	**4.03, brs**	62.3, CH	**4.08, t (4.5)**
3′	35.6, CH_2	**2.86, brd (14.0)**	35.8, CH_2	**2.82, d (4.5), 2H**
		**2.80, dd (14.0, 5.0)**		
4′	125.5, C		125.5, C	
5′/9′	130.7, CH	6.88, d (8.5)	130.7, CH	6.90, d (8.5)
6′/8′	114.8, CH	6.57, d (8.5)	114.8, CH	6.59, d (8.5)
7′	155.9, C		156.0, C	
OH-7′				
NH		9.18, s		9.19, s

[a] ^{1}H (600 MHz) and ^{13}C NMR (150 MHz) data in DMSO-d_6 in this study.

Table S6. Comparison of ^{1}H and ^{13}C NMR Data for Compounds **5**, **6** and Reported for Tolypyridones K and L in CD_3OD

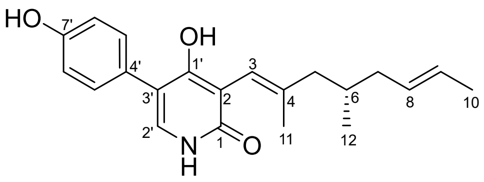

5

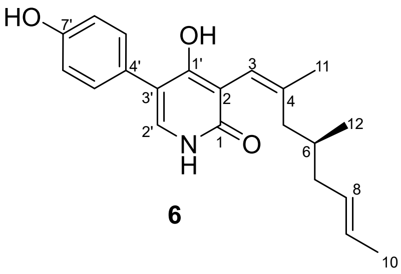

6

no.	**5** (CD_3OD) [a]		Tolypyridone K (CD_3OD) [b]		**6** (CD_3OD) [a]		Tolypyridone L (CD_3OD) [b]	
	δ_C, type	δ_H, mult. (J in Hz)	δ_C, type	δ_H, mult. (J in Hz)	δ_C, type	δ_H, mult. (J in Hz)	δ_C, type	δ_H, mult. (J in Hz)
1	165.0, C		165.2, C		165.0, C		165.0, C	
2	111.5, C		111.6, C		111.2, C		111.3, C	
3	116.8, CH	5.81, s	117.0, CH	5.77 [5.80 [c]], s	116.7, CH	5.82, s	117.2, CH	5.82, s
4	144.2, C		114.4 [144.4 [c]], C		145.5, C		145.1, C	
5α	48.1, CH_2	2.25, d (13.2, 6.0)	48.3, CH_2 [d]	2.25, dd (13.0, 5.9)	41.6, CH_2	2.02, d (13.2, 6.0)	46.9 [41.8 [c]], CH_2	2.02, dd (14.0, 5.6)
5β		2.00, dd (13.2, 8.4)		2.00, dd (13.0, 8.3)		1.77, dd (13.2, 7.2)		1.77, dd (14.0, 7.7)
6	32.6, CH	1.76, m	32.8, CH	1.75, m	32.6, CH	1.66, m	32.8, CH	1.37 [1.67 [c]], m
7α	41.1, CH_2 [c]	2.10, m	41.0 [41.3 [c]], CH_2	2.10, m	40.8, CH_2	1.90, m	41.3 [41.0 [c]], CH_2	1.90, m
7β		1.86, m		1.85, m		1.68, m		1.68, m
8	131.1, CH	5.47, m	131.5 [131.3 [c]], CH	5.46, m	130.8, CH	5.35, m	131.3 [131.0 [c]], CH	5.31, m
9	127.1, CH	5.46, m	127.3, CH	5.46, m	127.2, CH	5.32, m	127.3, CH	5.32, m
10	18.2, CH_3	1.65, d (4.2)	18.3, CH_3	1.64, d (4.1)	18.2, CH_3	1.58, d (4.8)	18.3, CH_3	1.58, d (5.1)
11	18.5, CH_3	1.58, s	23.9 [18.7 [c]], CH_3 [a]	1.59, s	23.7, CH_3	1.90, d (1.8)	18.9 [23.9 [c]], CH_3	1.90, d (1.4)
12	20.0, CH_3	0.93, d (6.6)	20.1, CH_3	0.96, d (6.5)	19.9, CH_3	0.76, d (6.6)	20.3 [20.1 [c]], CH_3	0.76, d (6.3)
1′	163.3, C		164.3 [163.4 [c]], C		163.3, C		164.3, C	
2′	132.7, CH	7.19, s	132.7 [132.8 [c]], CH	7.20, s	132.7, CH	7.20, s	132.7, CH	7.20, s
3′	117.3, C [c]		117.7 [117.5 [c]], C		117.2, C		117.7, C	
4′	126.5, C		127.2 [126.8 [c]], C		126.8, C		126.8 [127.2 [c]], C	
5′/9′	131.5, CH	7.25, d (8.4)	131.5 [131.7 [c]], CH	7.24, brd (8.4)	131.4, CH	7.26, d (8.4)	131.7 [131.6 [c]], CH	7.26, brd (8.7)
6′/8′	116.2, CH	6.82, d (8.4)	116.2 [116.4 [c]], CH	6.81, brd (8.4)	116.1, CH	6.81, d (8.4)	116.4 [116.2 [c]], CH	6.80, brd (8.7)
7′	158.2, C		158.3, C		158.0, C		158.1, C	

[a] ^{1}H (600 MHz) and ^{13}C NMR (150 MHz) data were obtained in CD_3OD in this study. [b] ^{1}H (800 MHz) and ^{13}C NMR (200 MHz) data were reported in CD_3OD in the NMR Table in the literature.[12] [c] The data written in red in the bracket [ ] were tentatively assigned and revised by us according to the presented 1D NMR spectrum in the Supporting Information affiliated with reference [12].

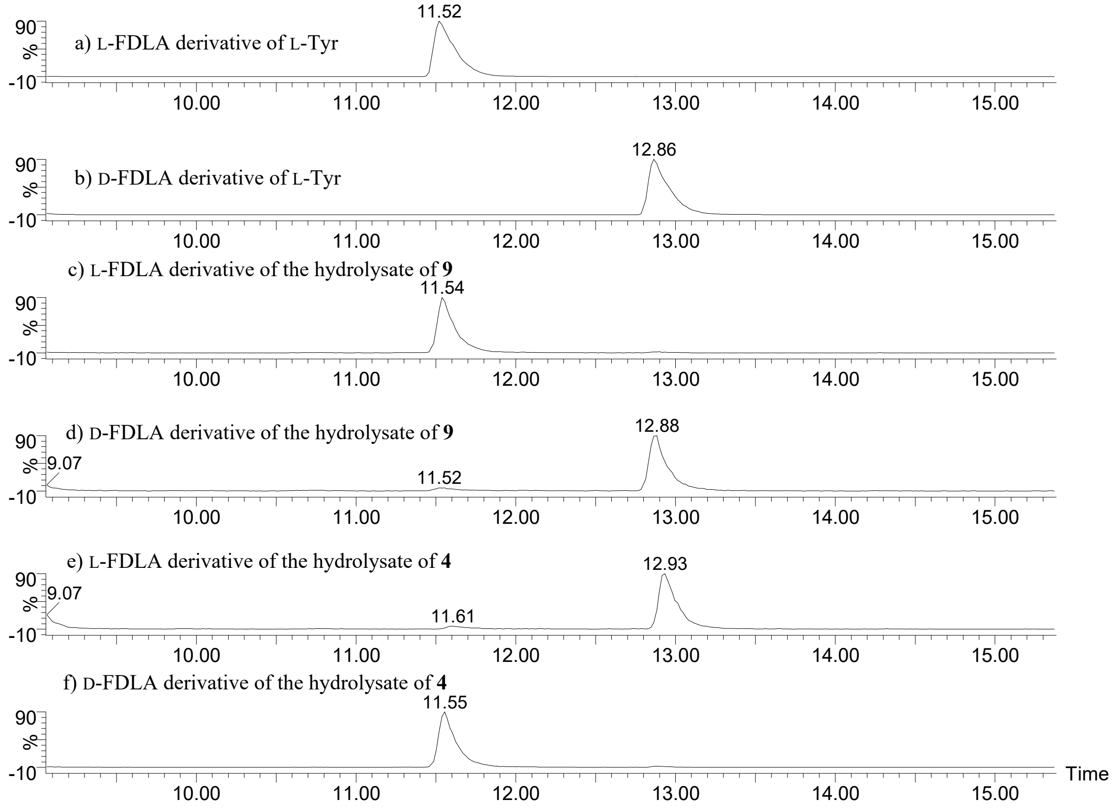

Figure S2. The Marfey's analysis of the hydrolysates of compounds **4** and **9**. Extracted ion chromatogram at *m/z* 476.18 for the FDLA derivative of Tyr.

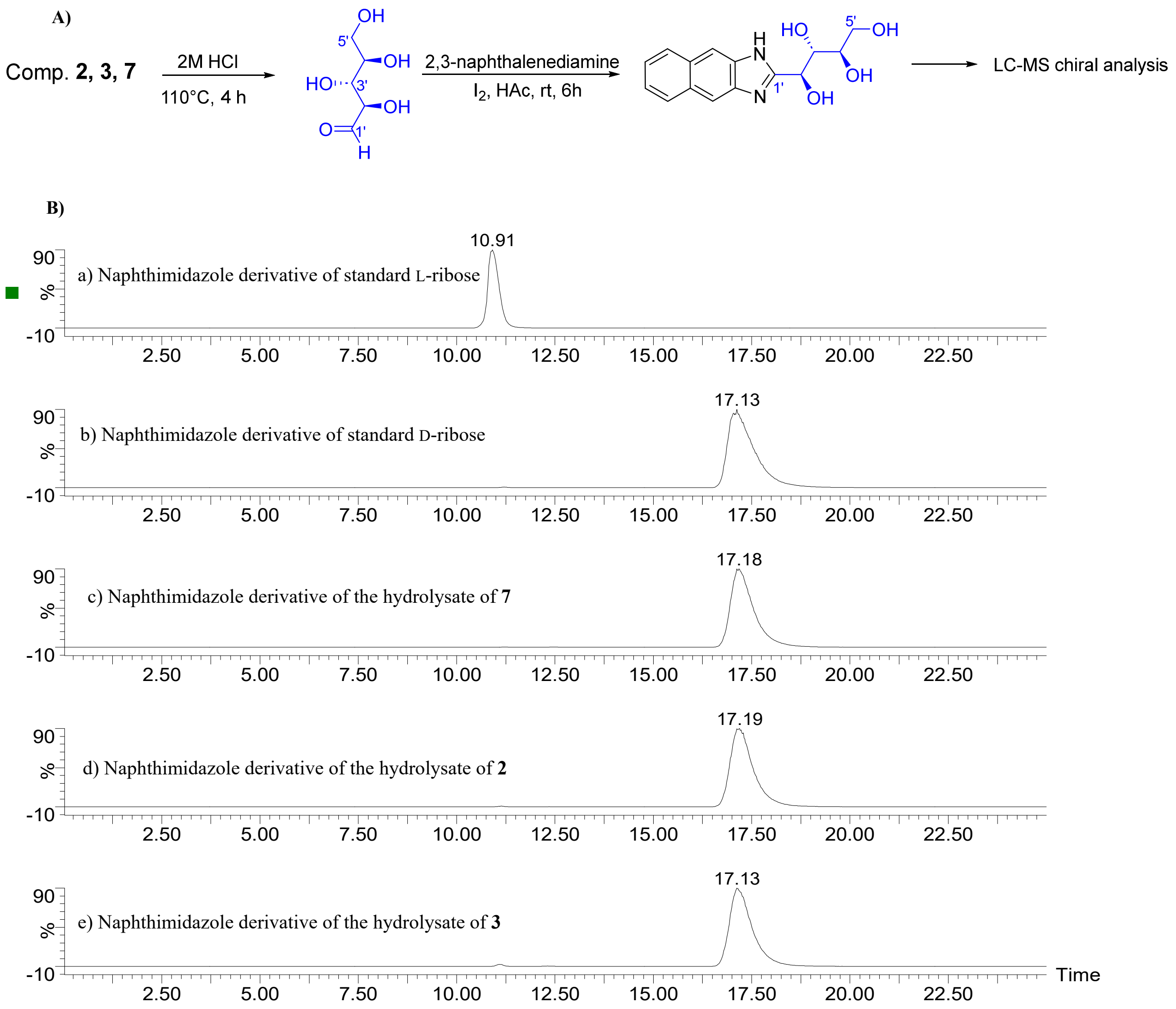

Figure S3. Chromatographic determination of the absolute configuration of the ribose residue in **2**, **3** and **7** by naphthimidazole derivatization. (A) Procedure for preparation of the sugar naphthimidazole derivative. (B) LC-MS analysis of the ribose naphthimidazole derivative (extracted ion chromatogram at *m/z* 289.1).

Table S7. The Deduced Functions of ORFs in Tolypyridone C Biosynthetic Gene Cluster from *Tolypocladium* sp. CPCC401485

Tolypocladium sp. CPCC401485	*Tolypocladium* sp. 49Y	GenBank no.	Identity/Simlarity%	Propose function
Orf1	TolC	QPC57091.1	80/86	enoyl reductase
Orf2	TolA	QPC57090.1	87/93	PKS-NRPS
Orf3	TolD	QPC57089.1	78/84	cytochrome P450
Orf4	TolB	QPC57088.1	94/96	cytochrome P450

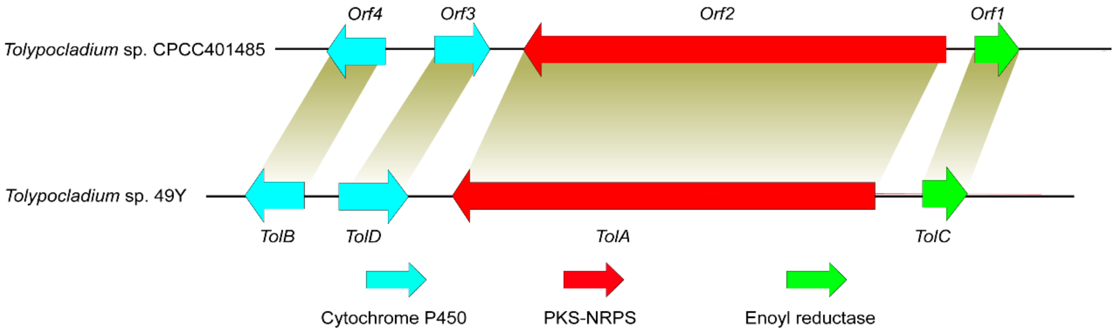

Figure S4. Graphical comparison of the tolypyridone C biosynthetic gene clusters from *Tolypocladium* sp. CPCC401485 and *Tolypocladium* sp. 49Y

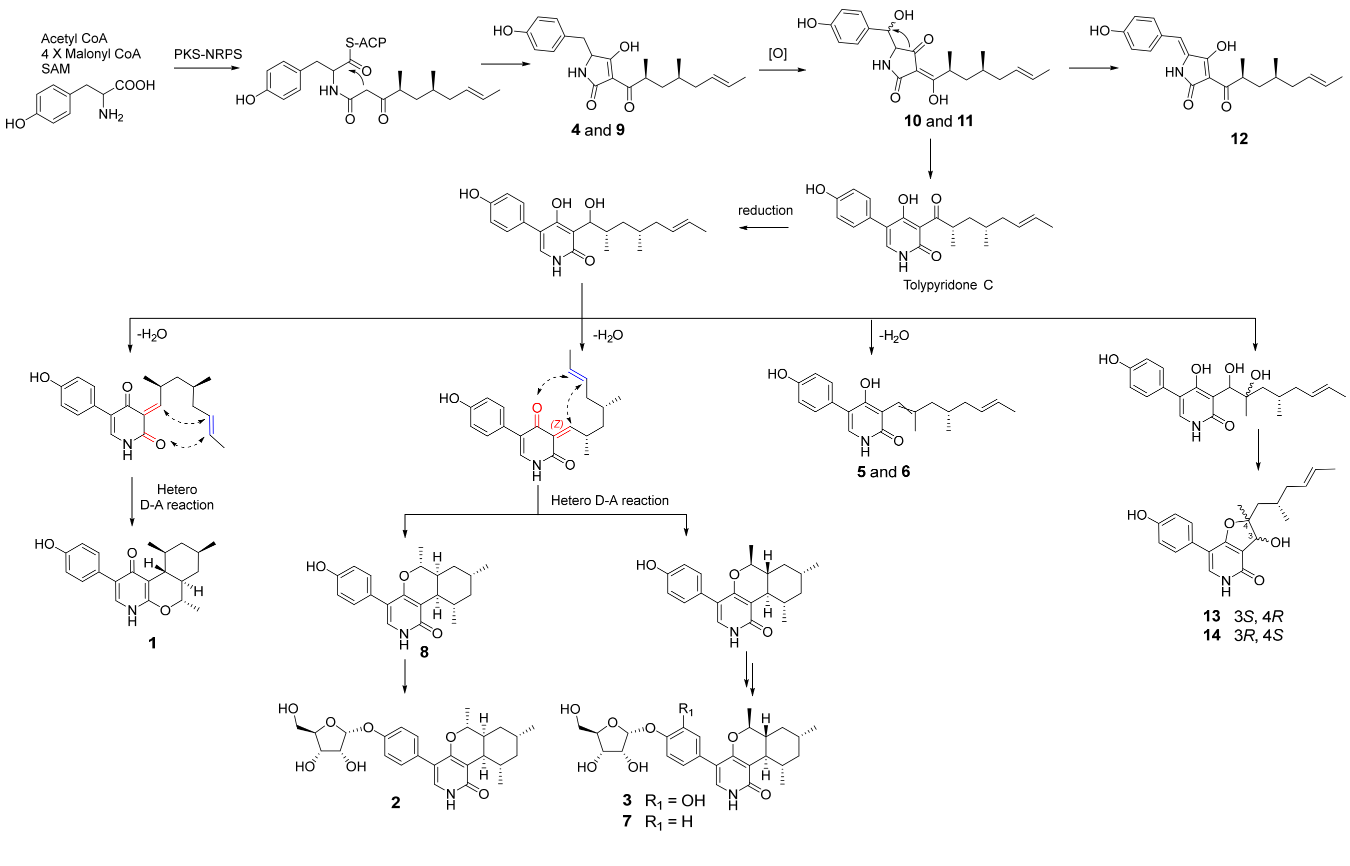

Figure S5. Proposed biosynthetic pathway of the compounds **1–14**

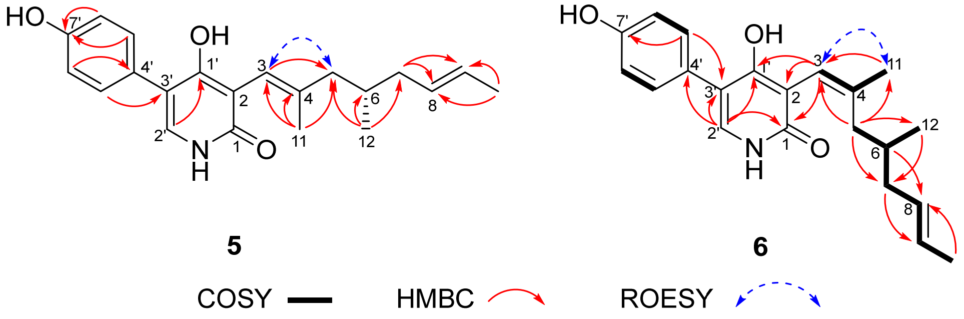

Figure S6. The key COSY, HMBC and ROESY correlations of compounds **5** and **6**.

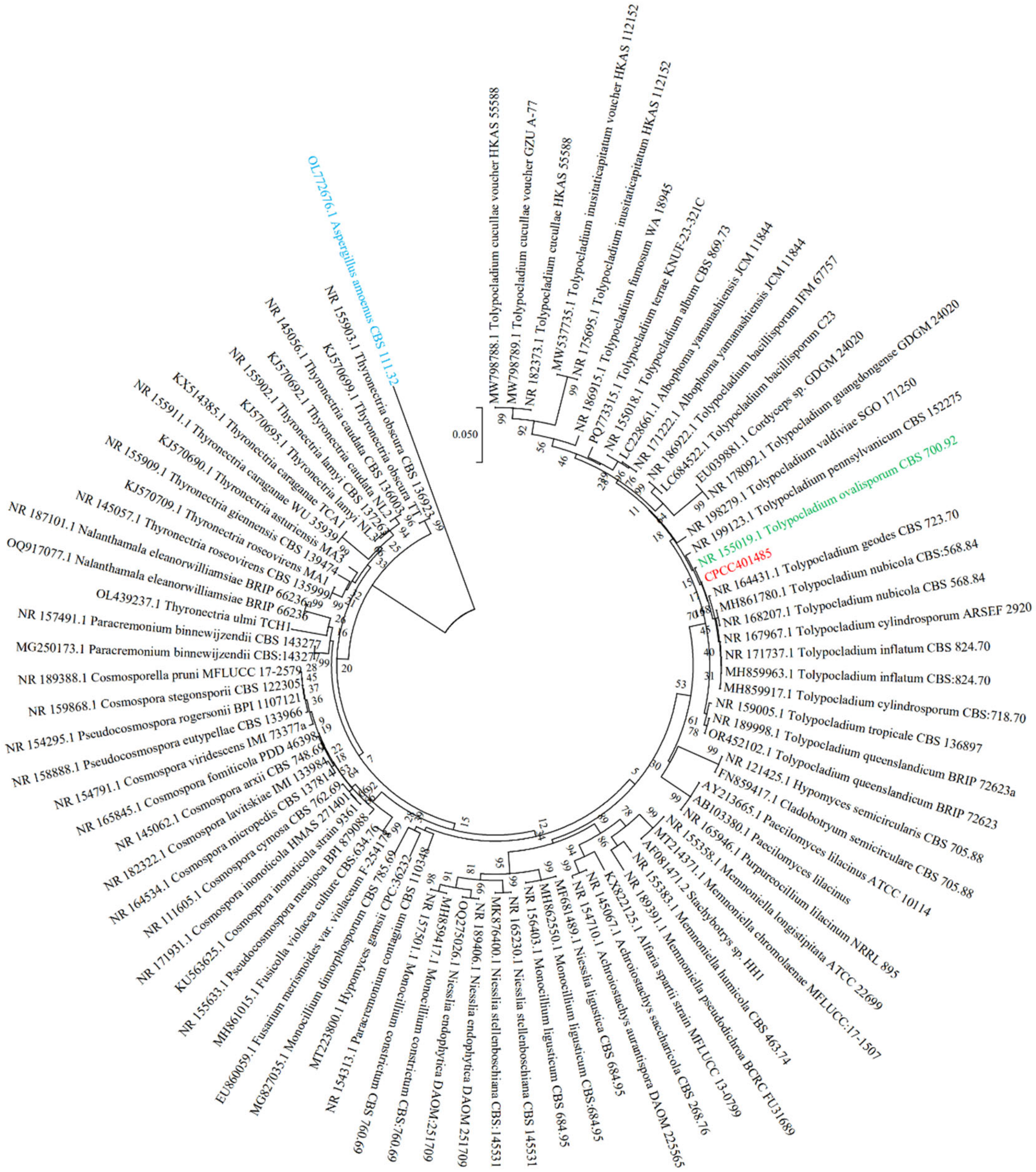

Figure S7. The phylogenetic tree for. *Tolypocladium* sp. CPCC401485 ITS sequence.

The evolutionary history was inferred using the Neighbor-Joining method [14]. The optimal tree with the sum of branch length = 1.16454463 is shown. The numbers on the branches indicate the percentage bootstrap values of 1,000 replicates [15]; The evolutionary distances were computed using the Kimura 2-parameter method [16]. The scale bar represents 0.05 substitutions per nucleotide position. The analysis involved 86 nucleotide sequences. All positions containing gaps and missing data were eliminated. There were a total of 362 positions in the final dataset. The sequence of *Aspergillus amoenus* CBS 111.32 was used as the outgroup species. Evolutionary analyses were conducted in MEGA7 [17].

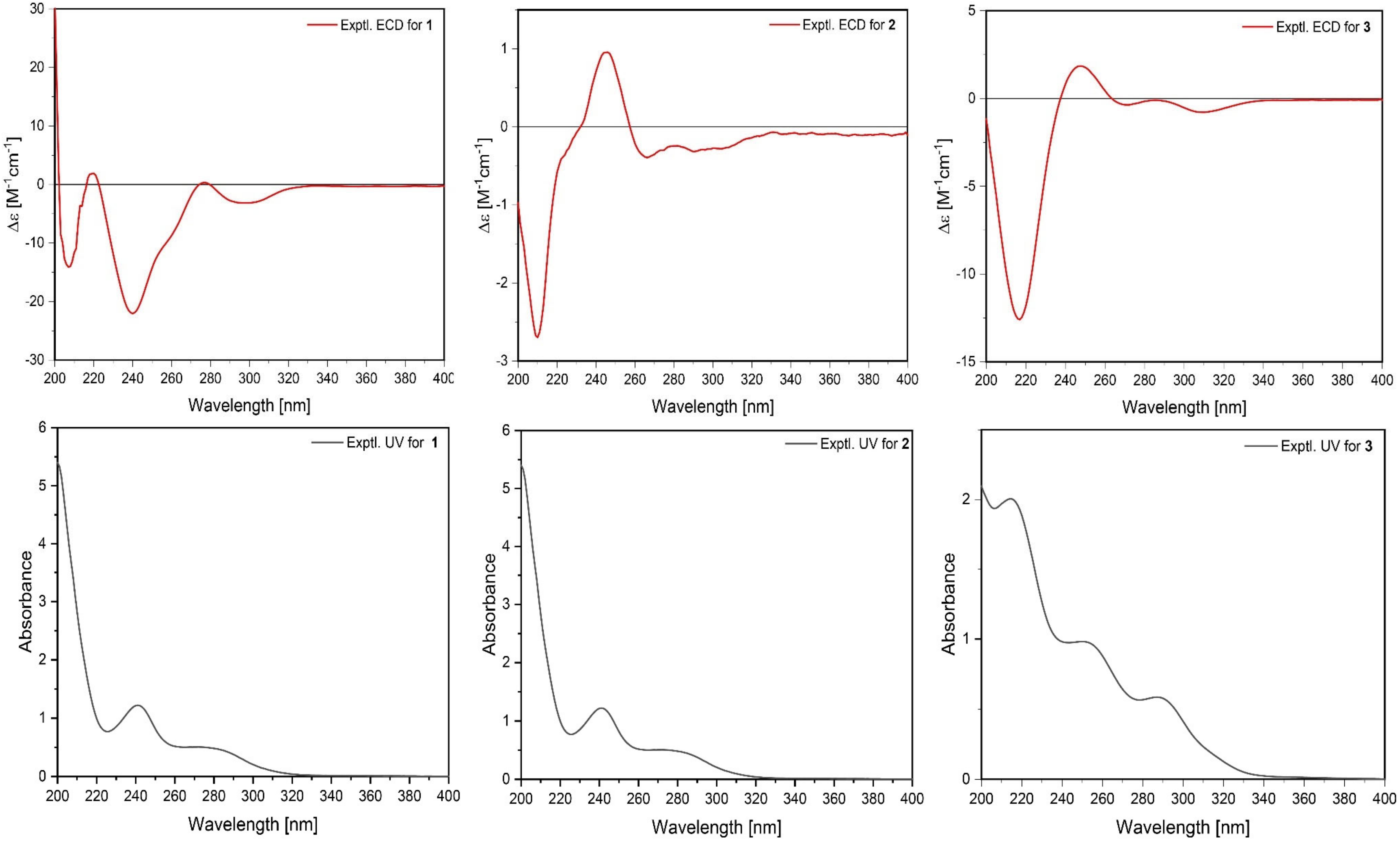

Figure S8. The experimental ECD and UV spectrum for compounds **1–3** in MeOH.

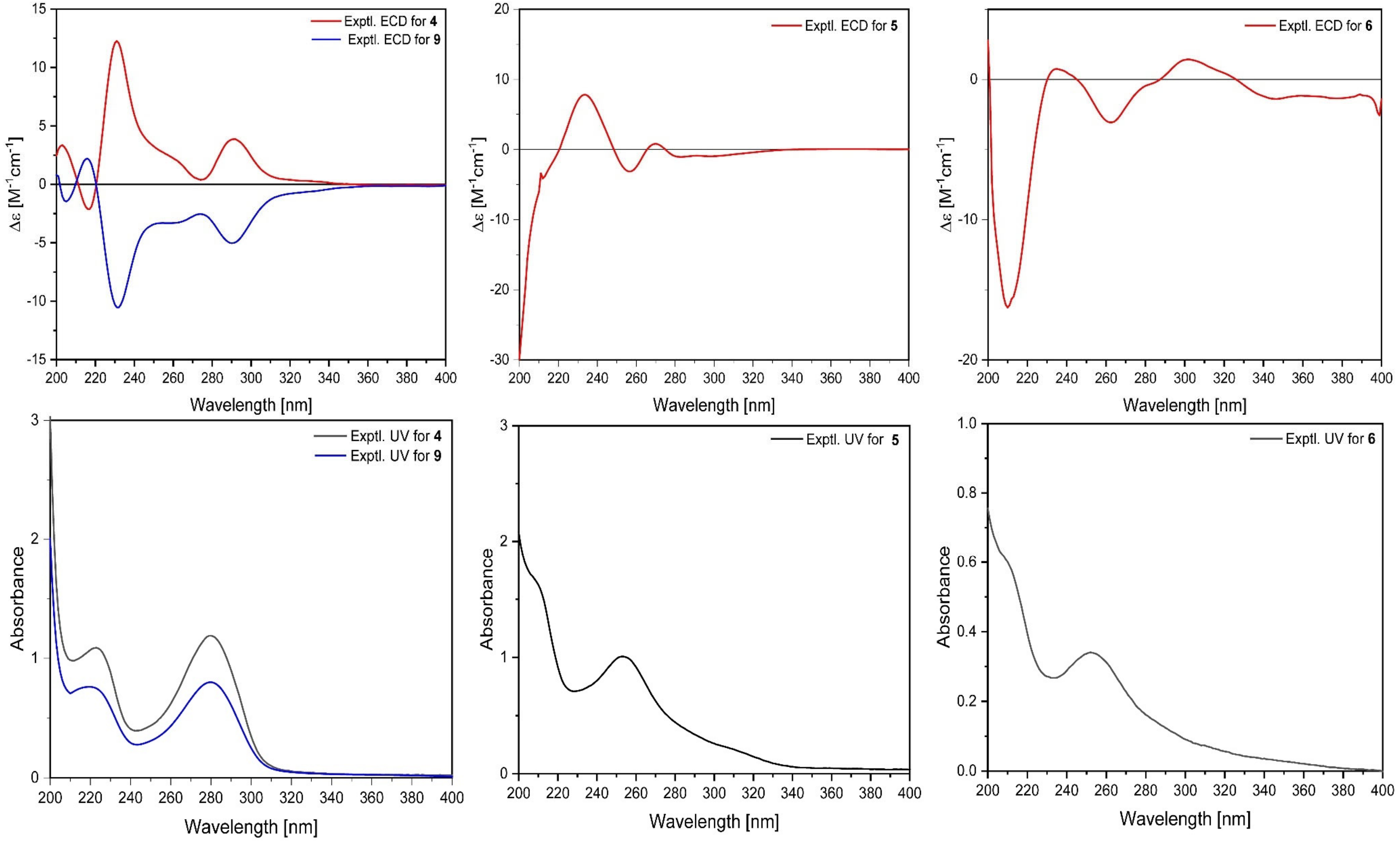

Figure S9. The experimental ECD and UV spectrum for compounds **4–6** in MeOH.

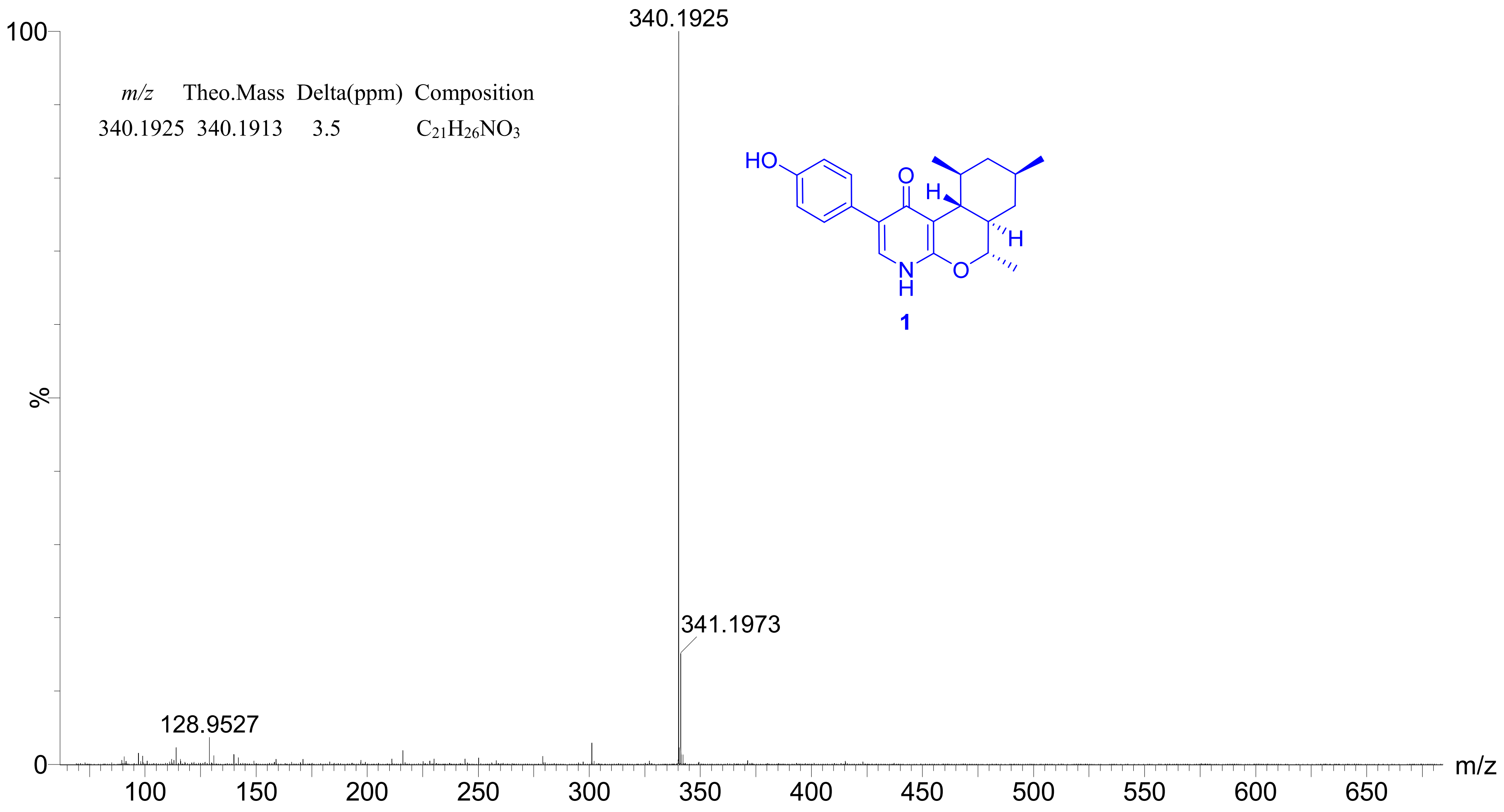

Figure S10. The (+)-HRESIMS spectrum of compound **1**.

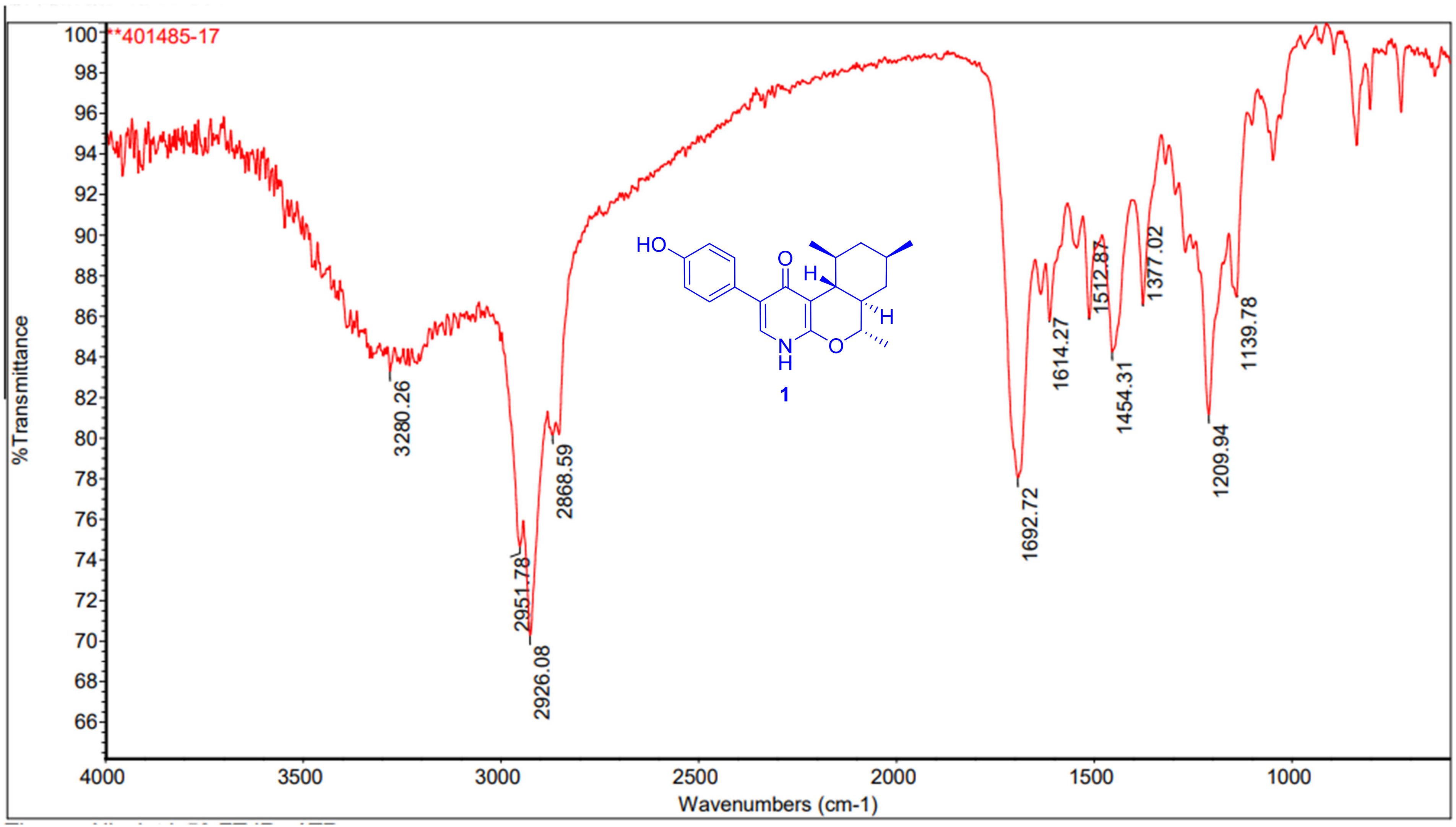

Figure S11. The IR spectrum of compound **1**.

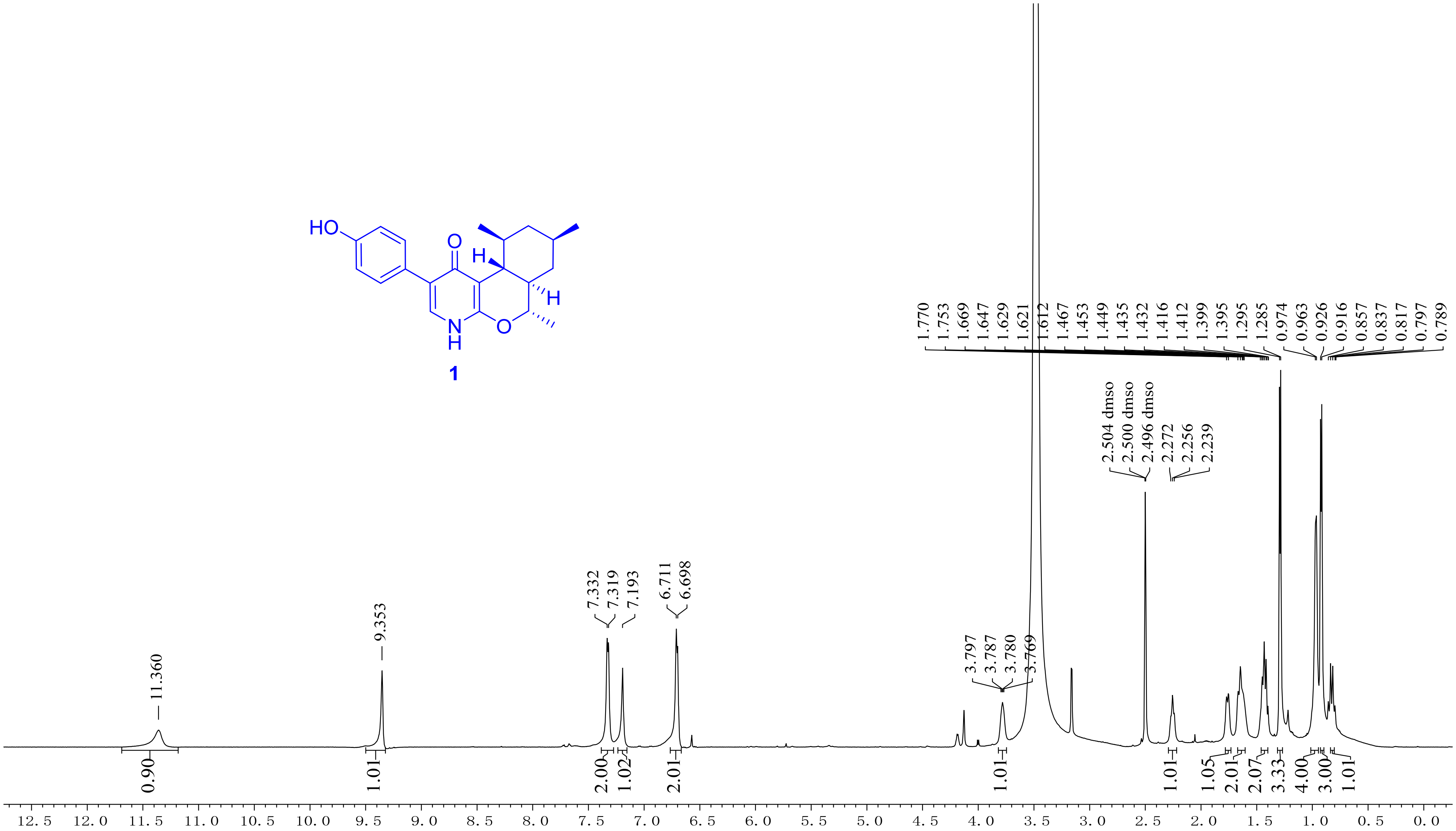

Figure S12. The ^{1}H NMR spectrum of compound **1** in DMSO-d_6 (600 MHz).

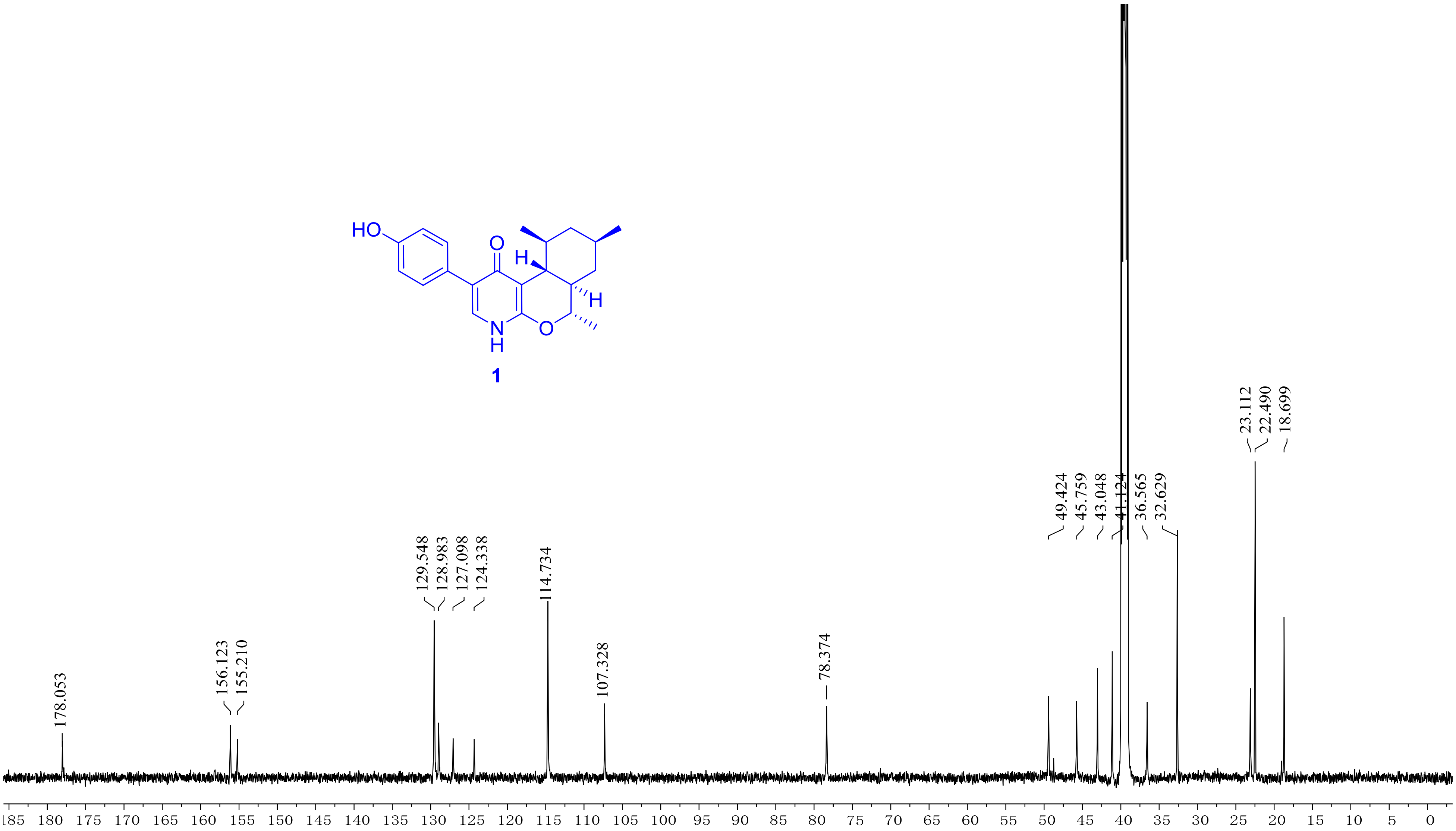

Figure S13. The ^{13}C NMR spectrum of compound **1** in DMSO-d_6 (150 MHz).

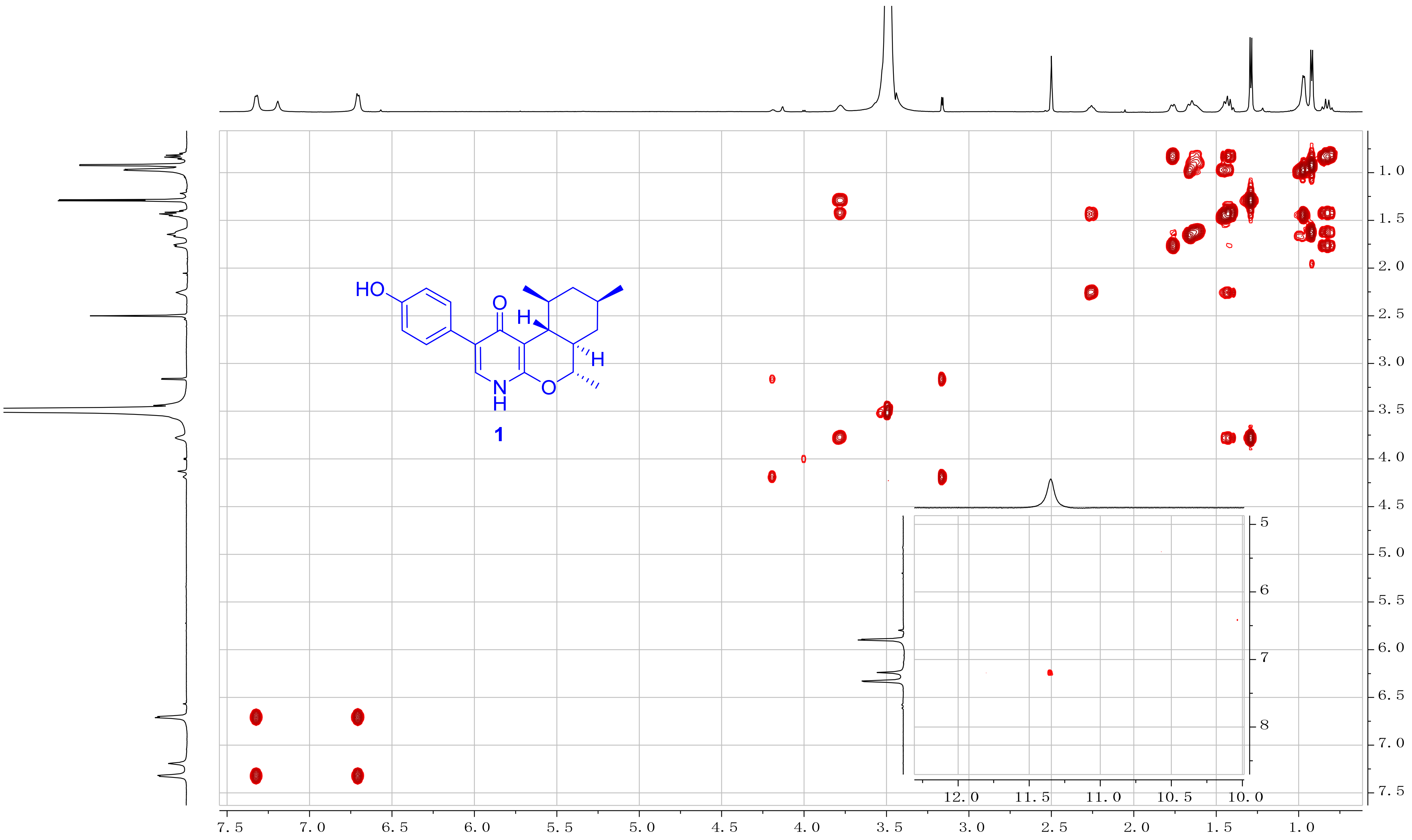

Figure S14. The ^{1}H-^{1}H COSY spectrum of compound **1** in DMSO-d_6 (600 MHz).

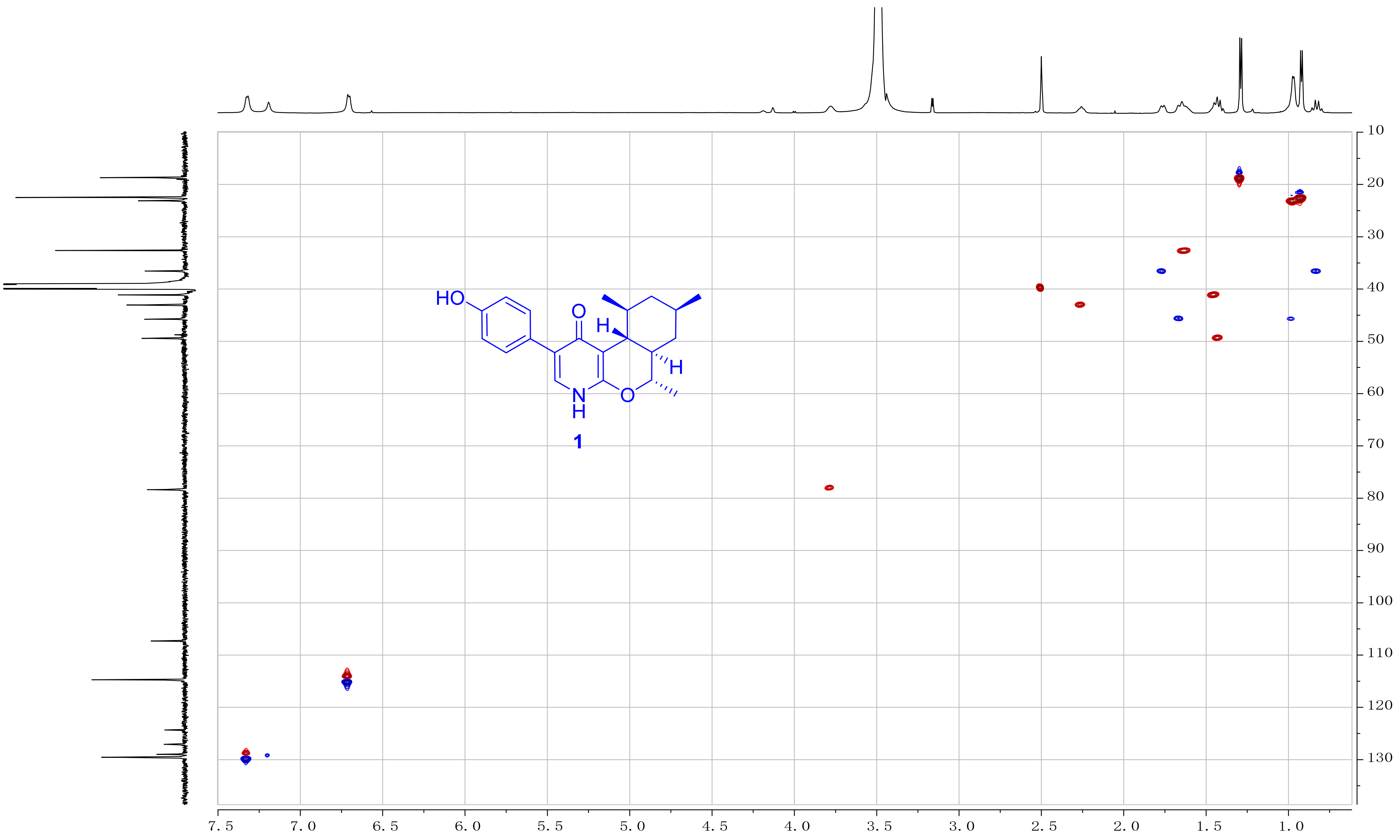

Figure S15. The HSQC spectrum of compound **1** in DMSO-d_6 (600 MHz).

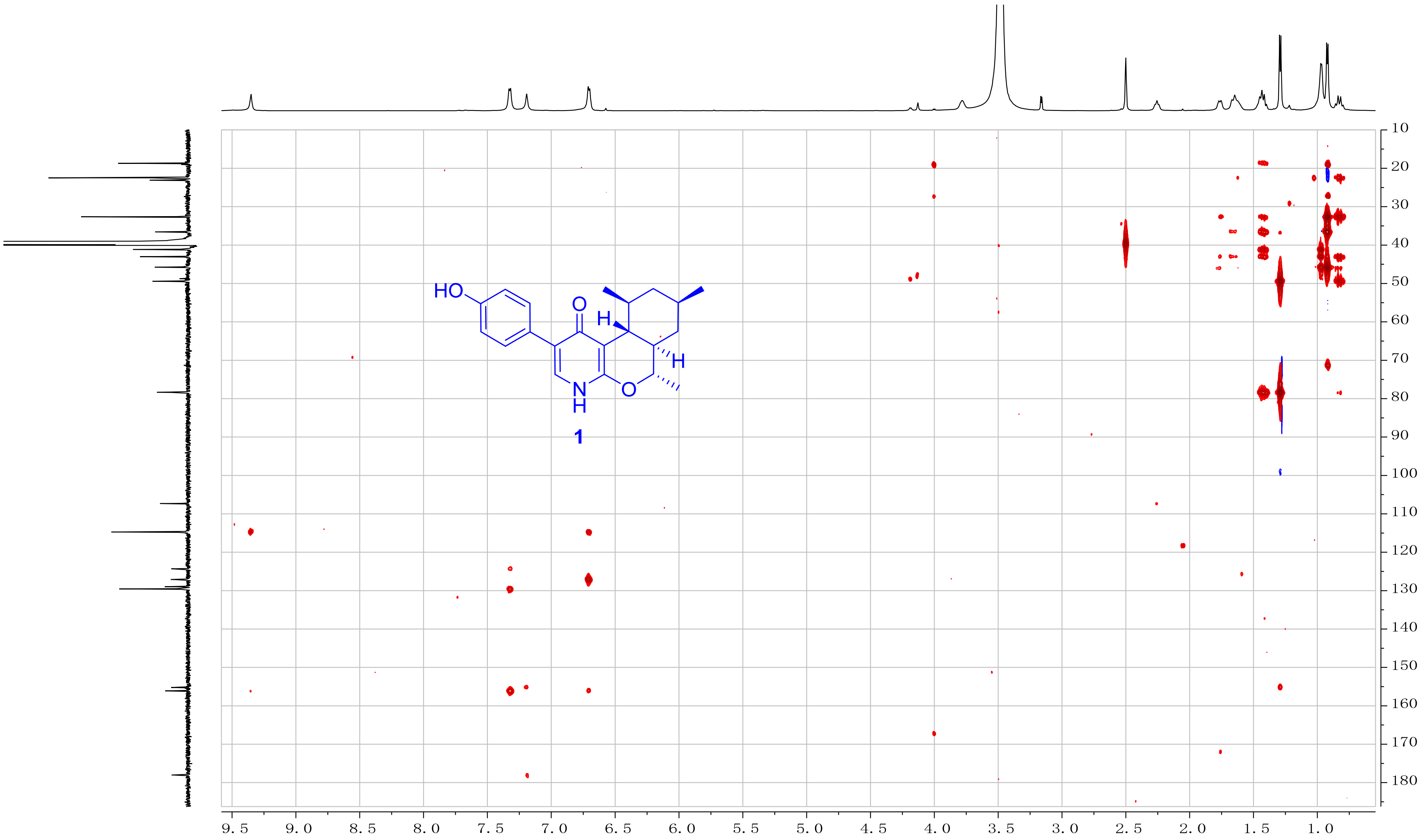

Figure S16. The HMBC spectrum of compound **1** in DMSO-d_6 (600 MHz).

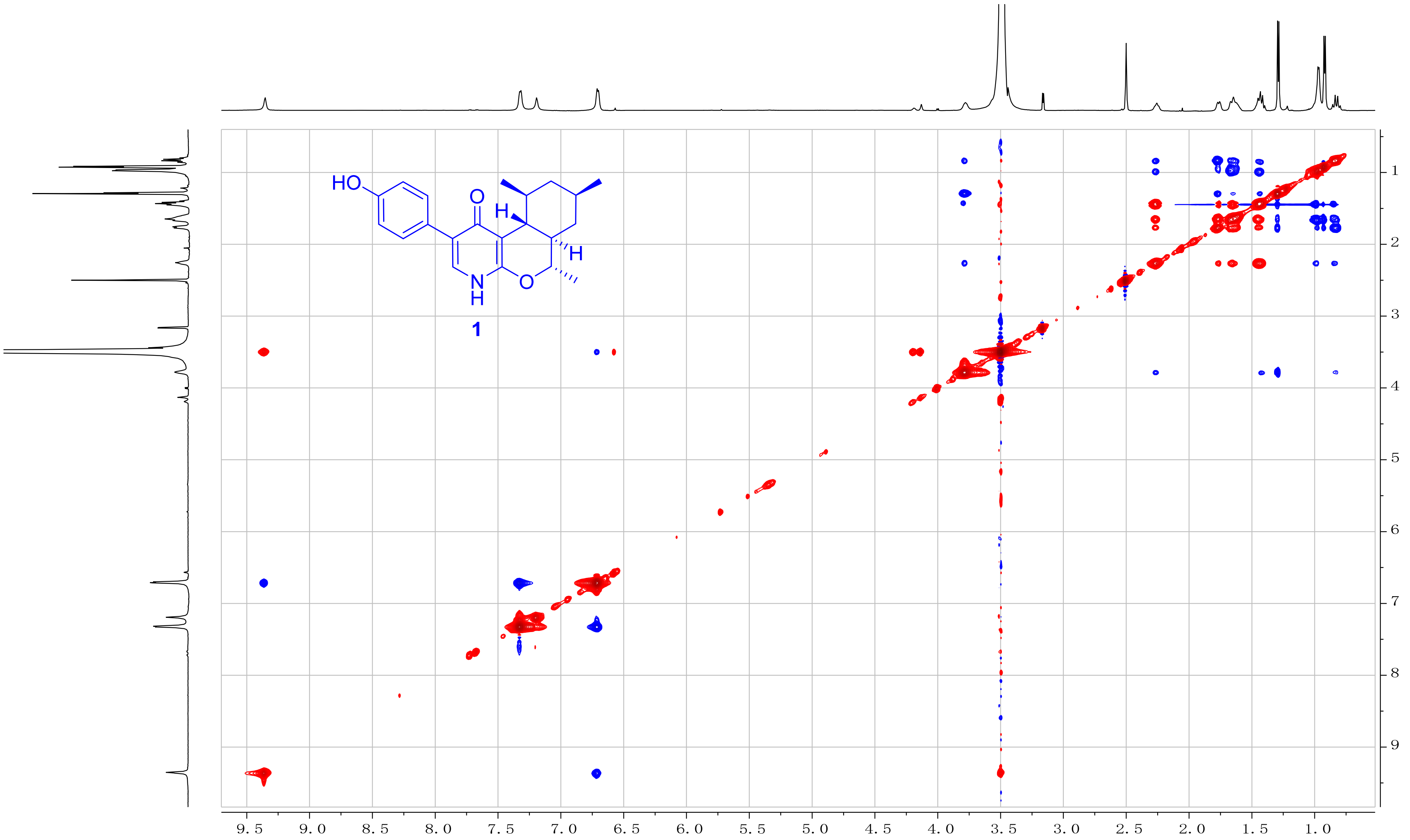

Figure S17. The ROESY spectrum of compound **1** in DMSO-d_6 (600 MHz).

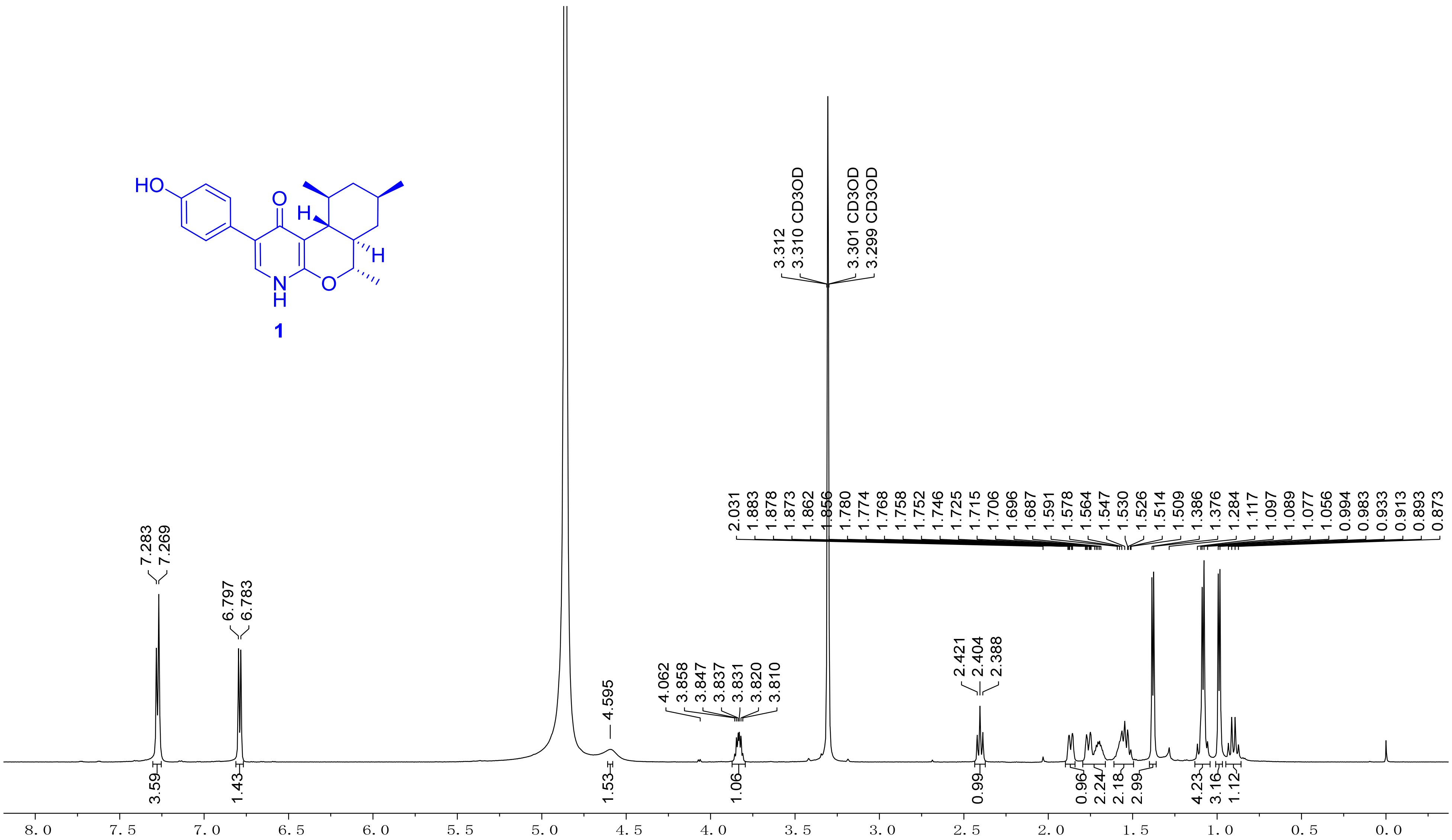

Figure S18. The ^{1}H NMR spectrum of compound **1** in CD_3OD (600 MHz).

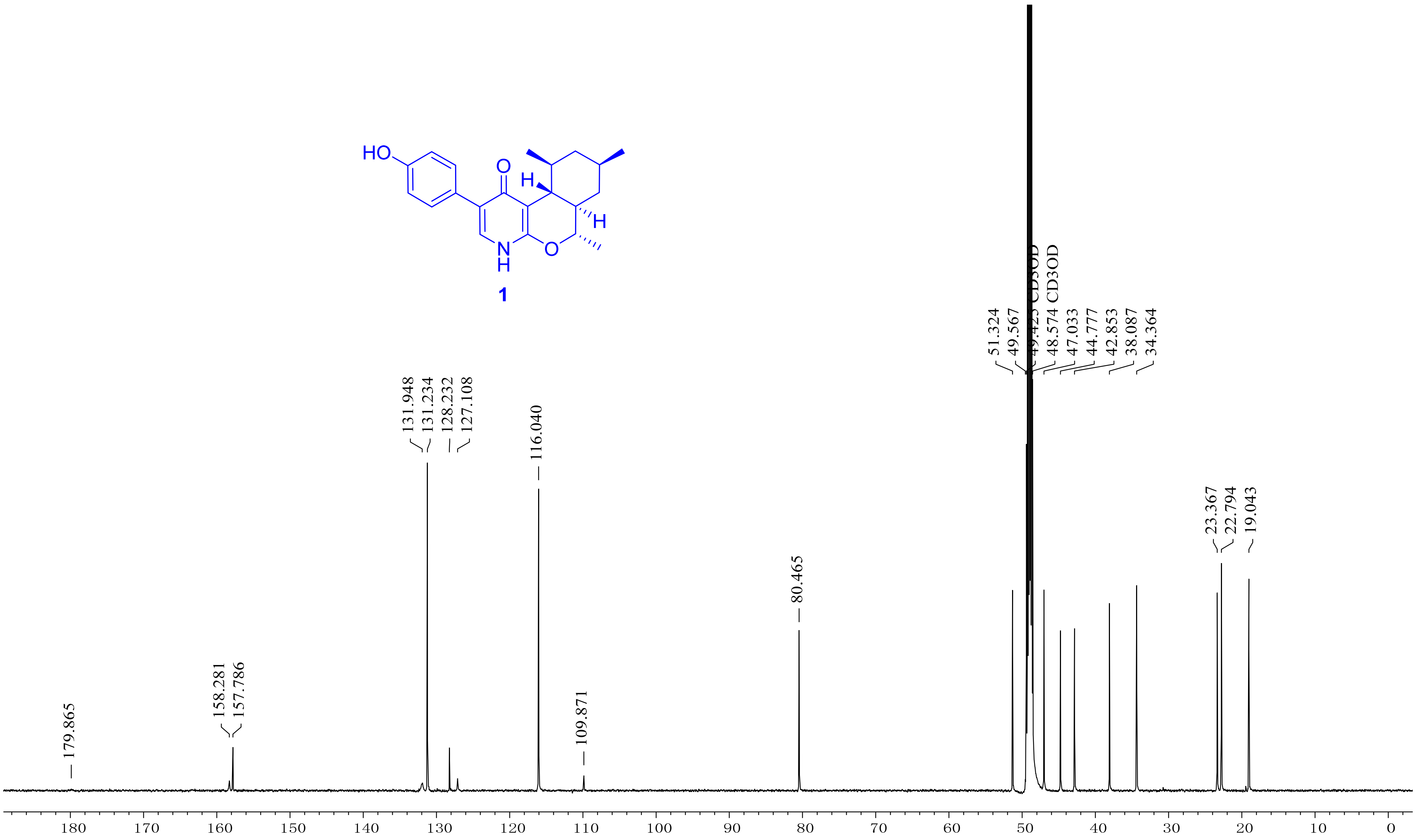

Figure S19. The ^{13}C NMR spectrum of compound **1** in CD_3OD (150 MHz).

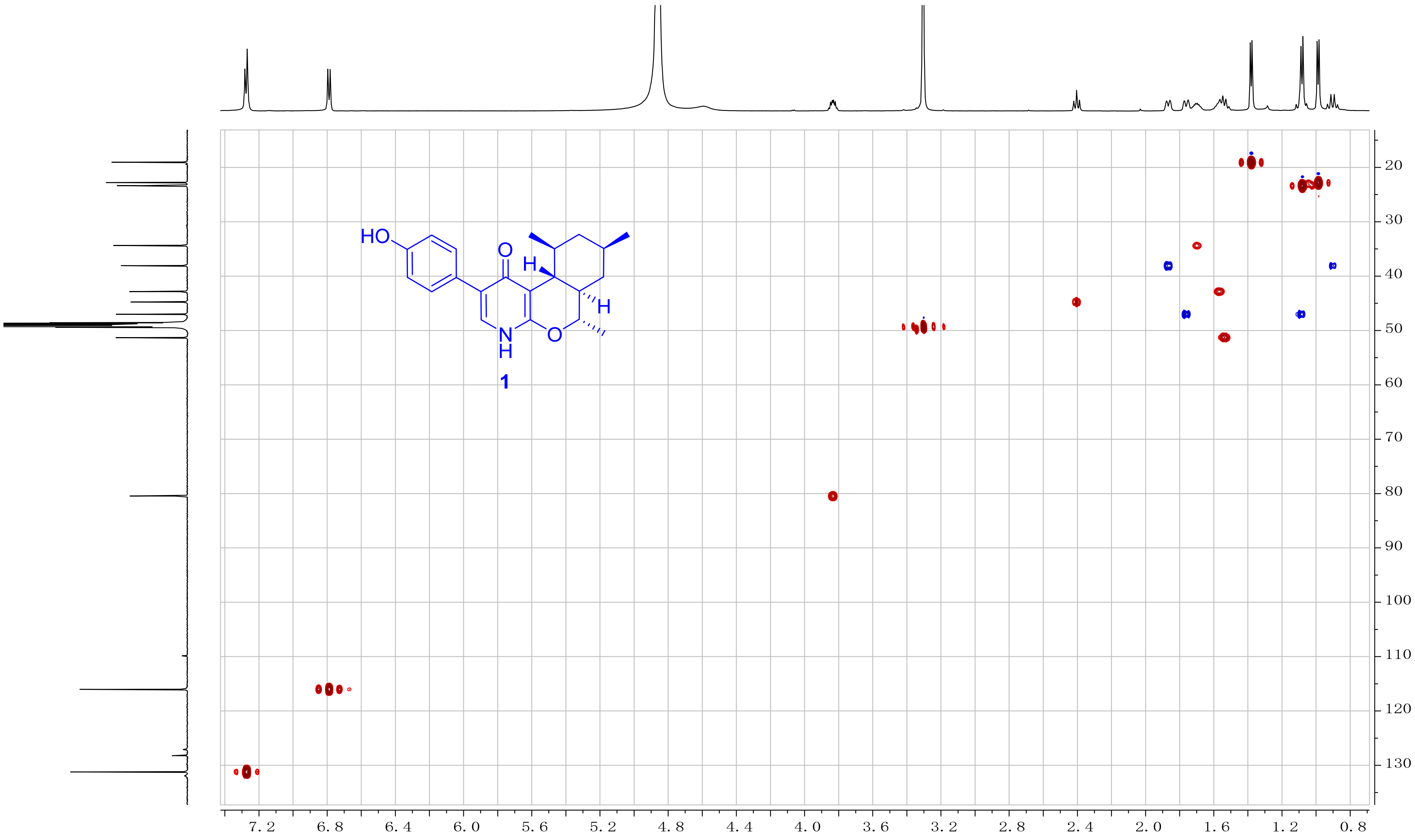

Figure S20. The HSQC spectrum of compound **1** in CD_3OD (600 MHz).

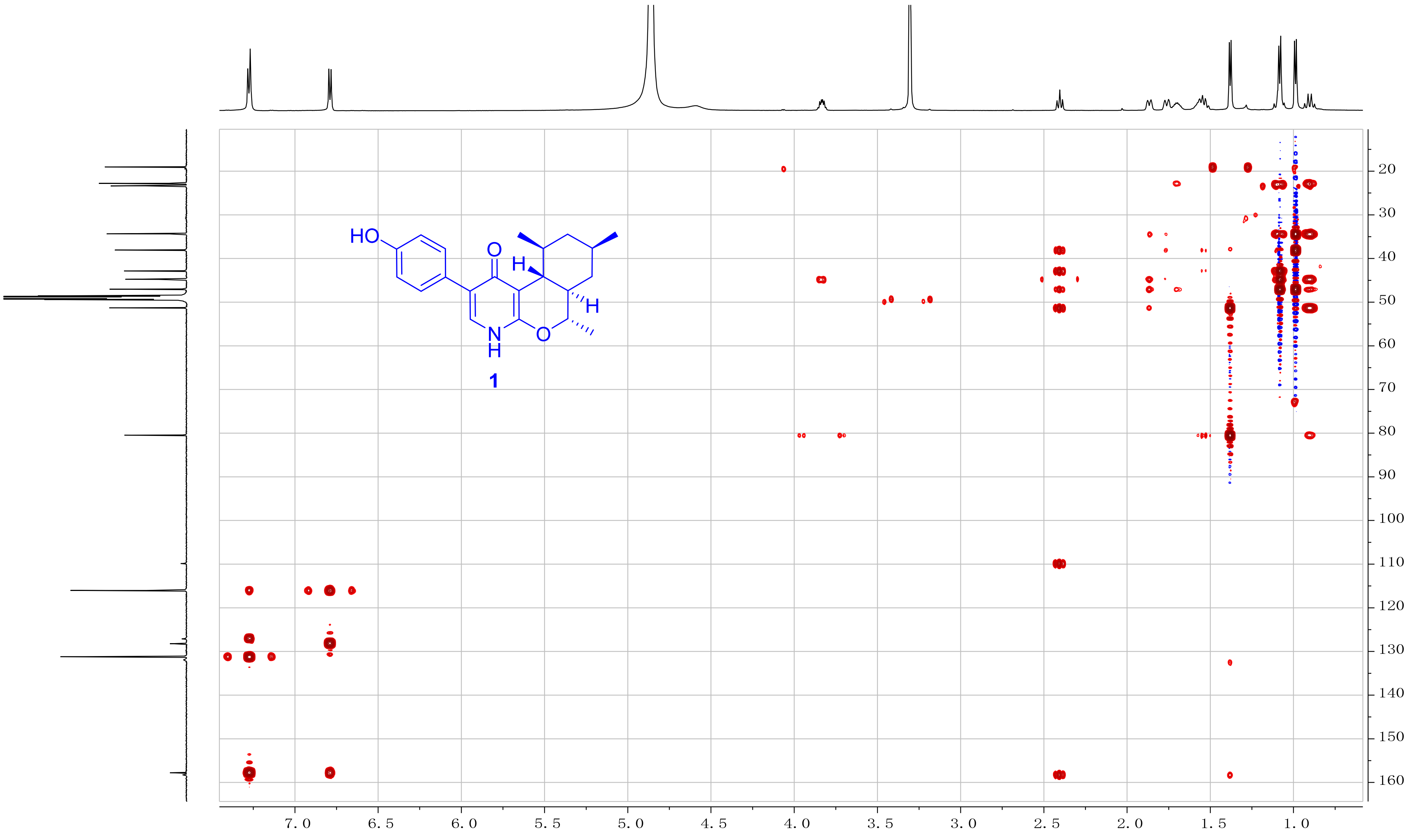

Figure S21. The HMBC spectrum of compound **1** in CD3OD (600 MHz).

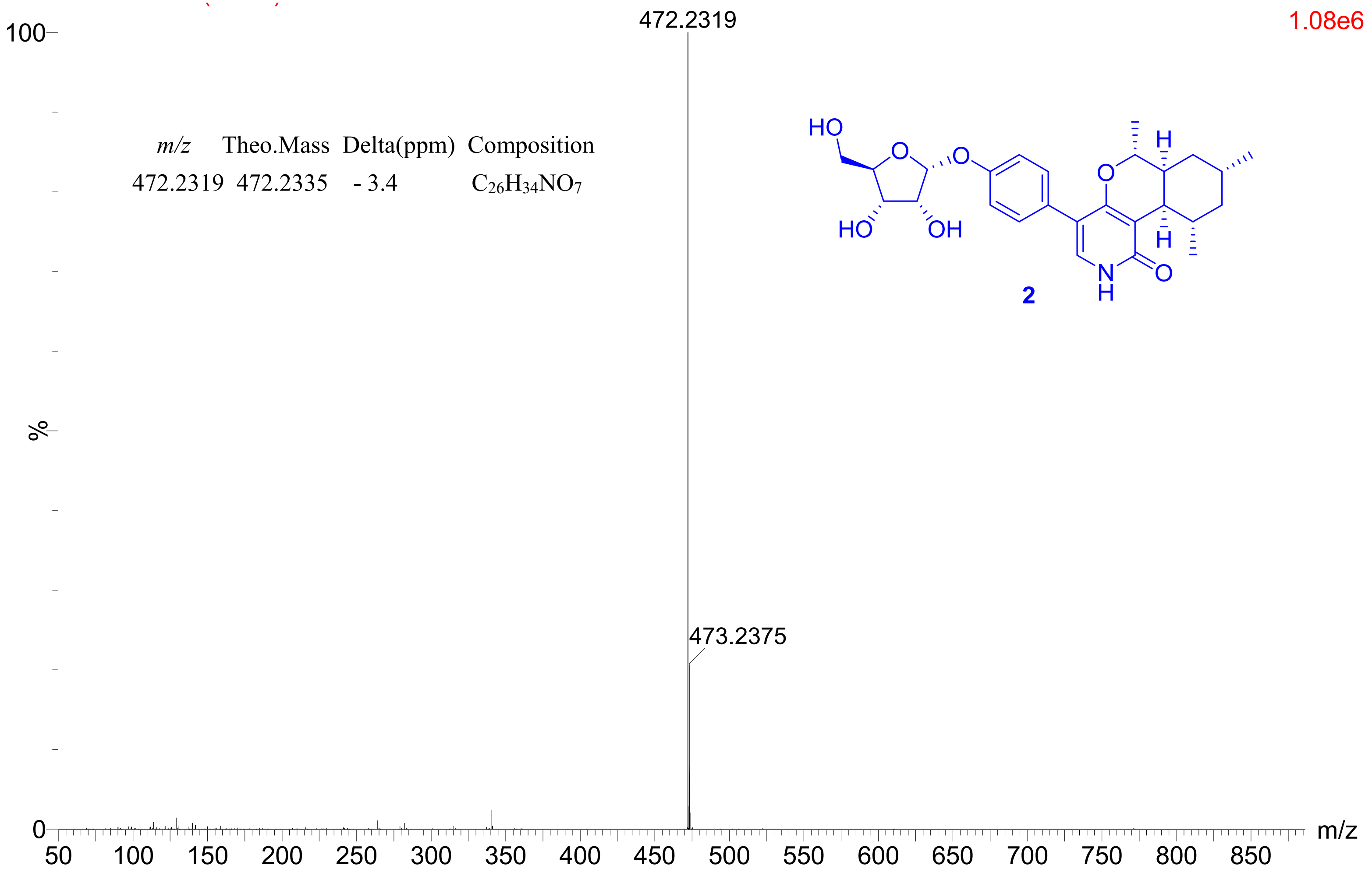

m/z	Theo.Mass	Delta(ppm)	Composition
472.2319	472.2335	- 3.4	$C_{26}H_{34}NO_7$

Figure S22. The (+)-HRESIMS spectrum of compound **2**.

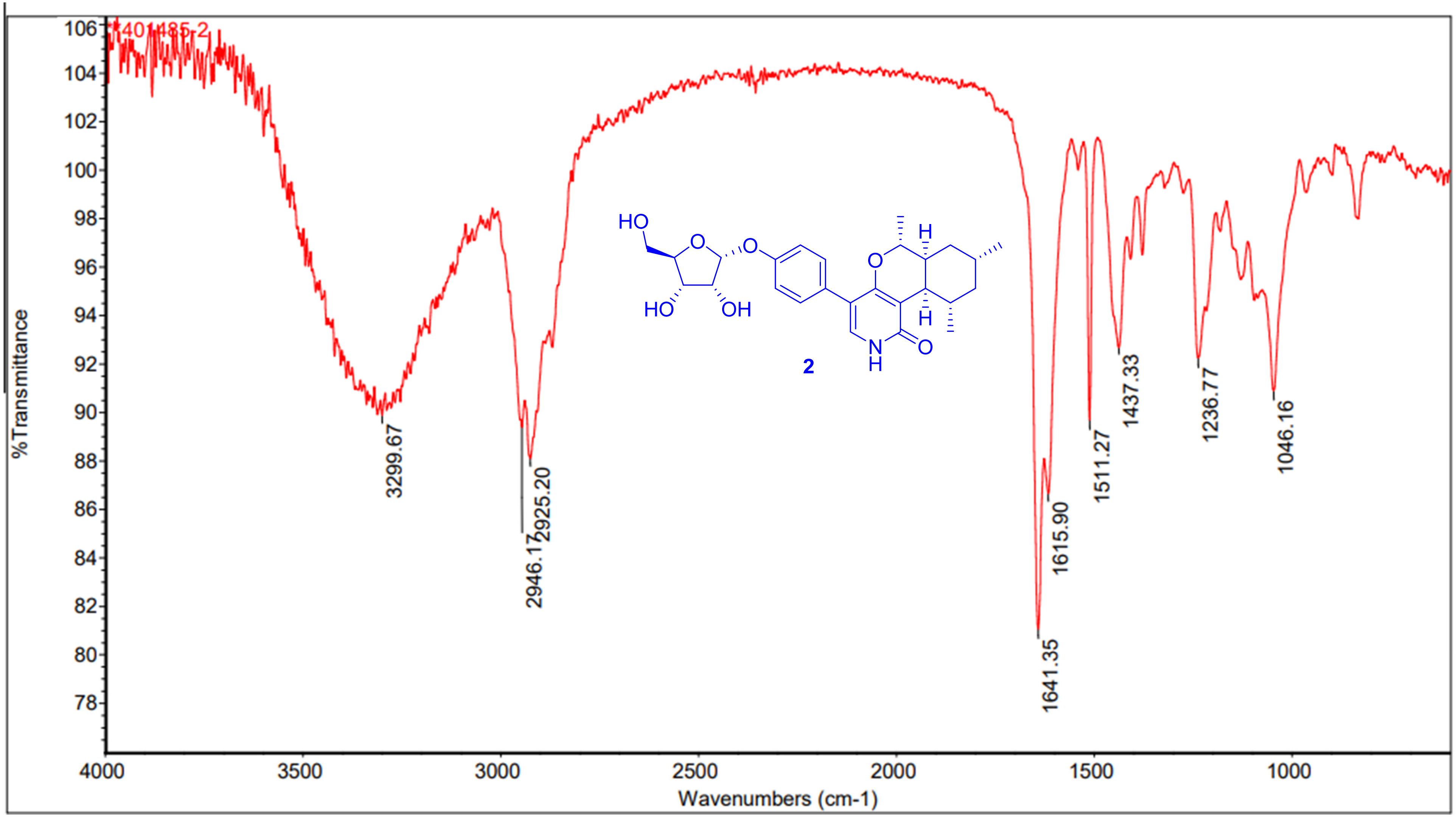

Figure S23. The IR spectrum of compound **2**.

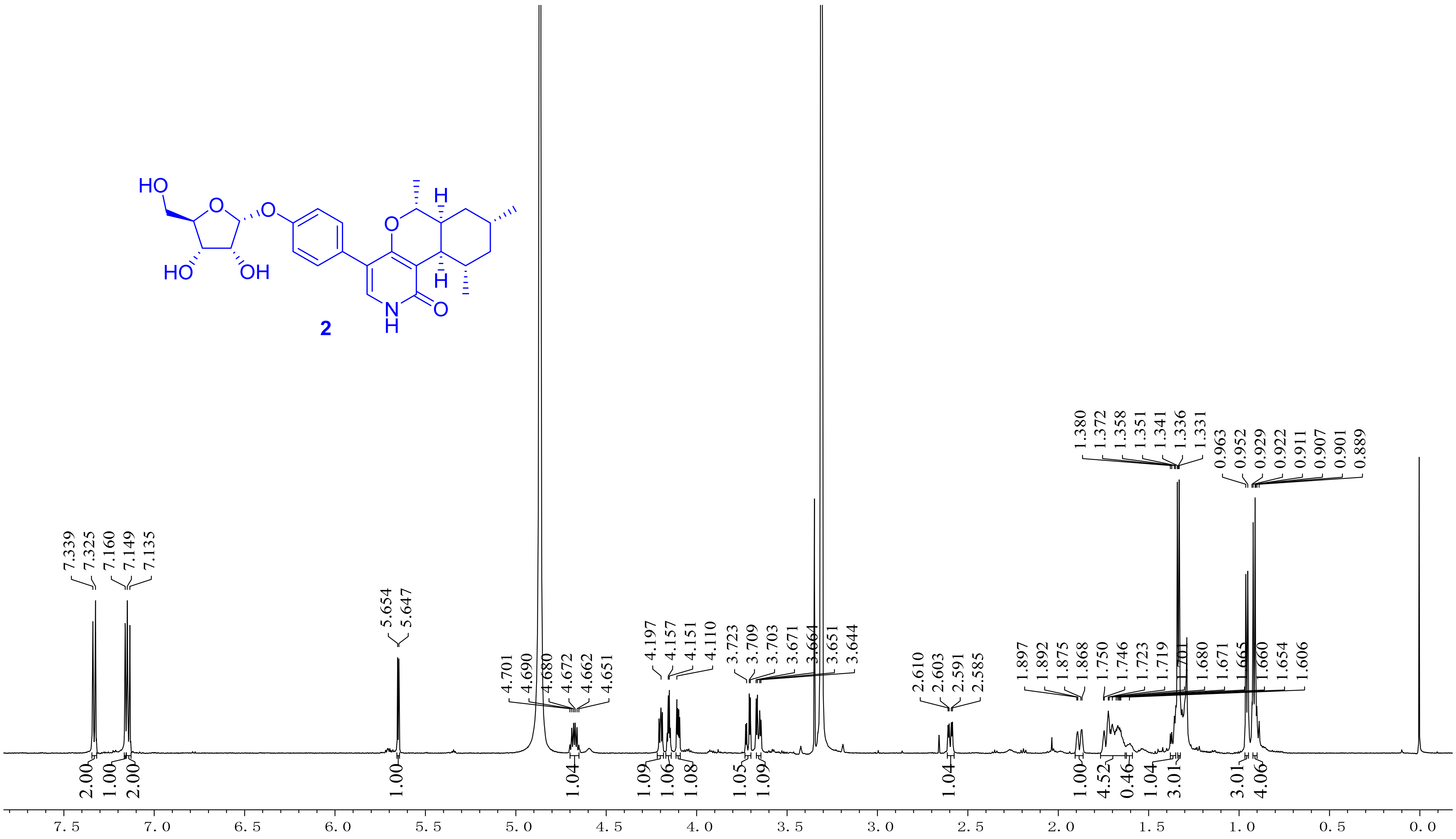

Figure S24. The ^{1}H NMR spectrum of compound **2** in CD3OD (600 MHz).

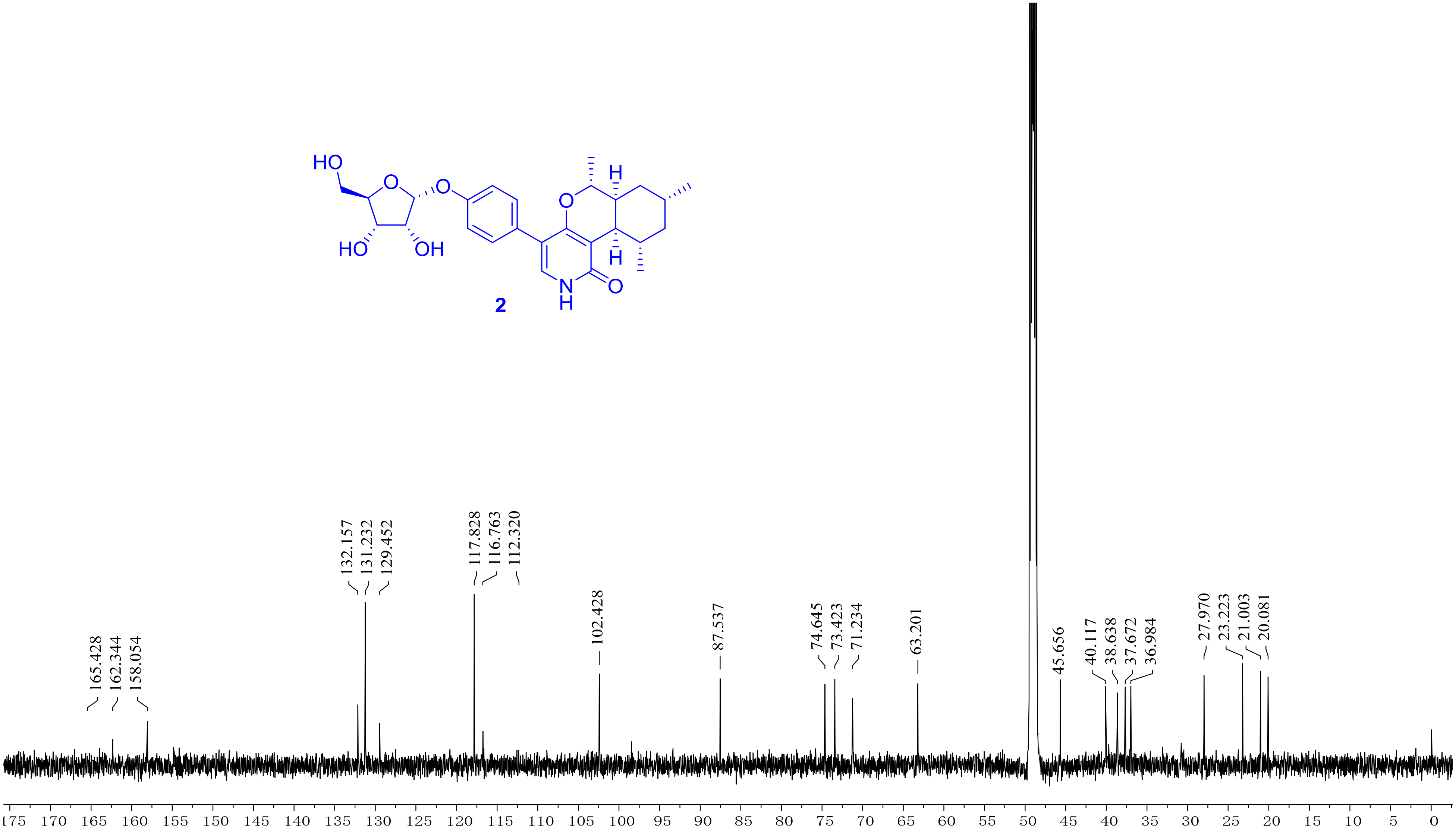

Figure S25. The ^{13}C NMR spectrum of compound **2** in CD_3OD (150 MHz).

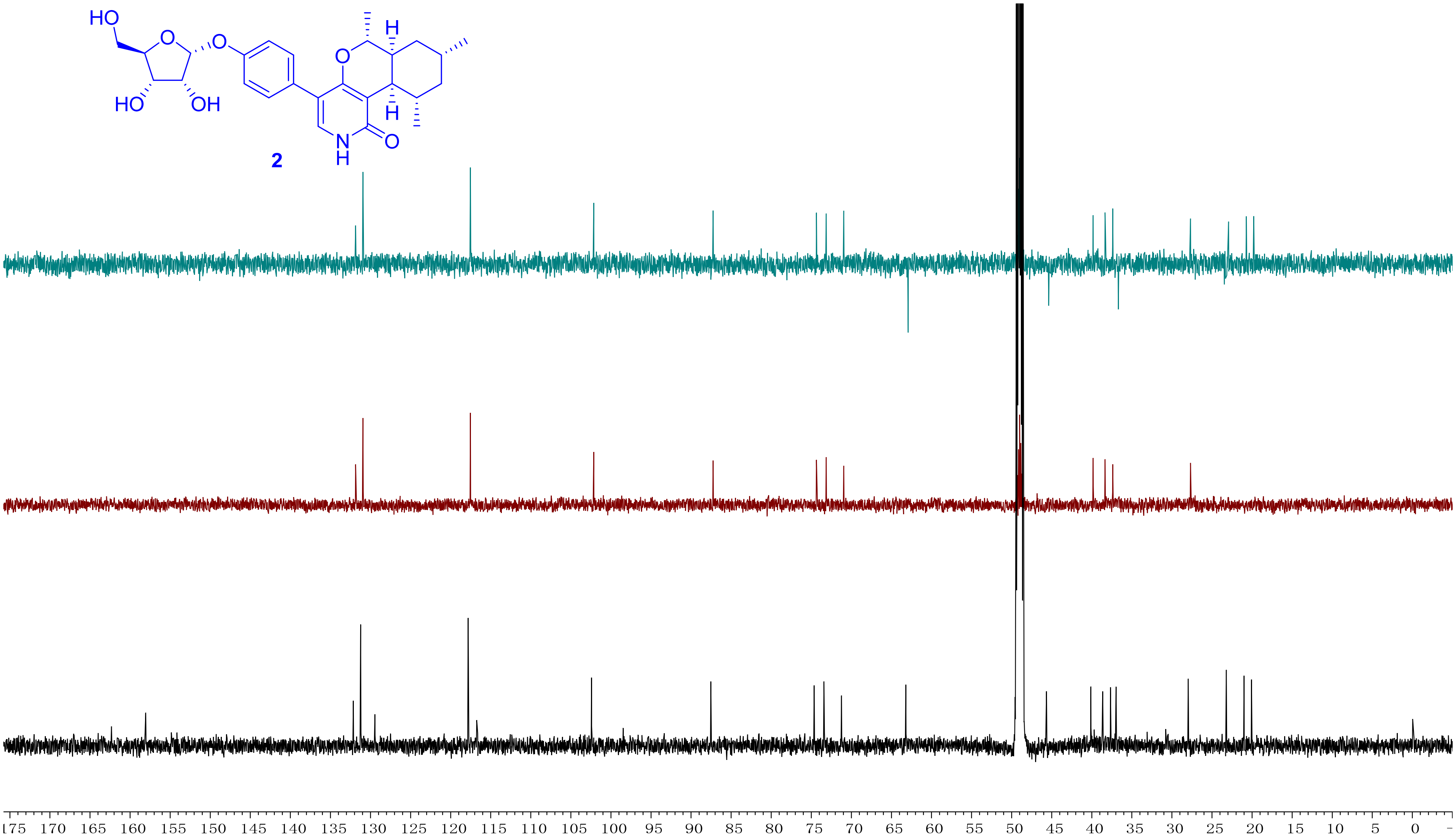

Figure S26. The DEPT spectrum of compound **2** in CD_3OD (150 MHz).

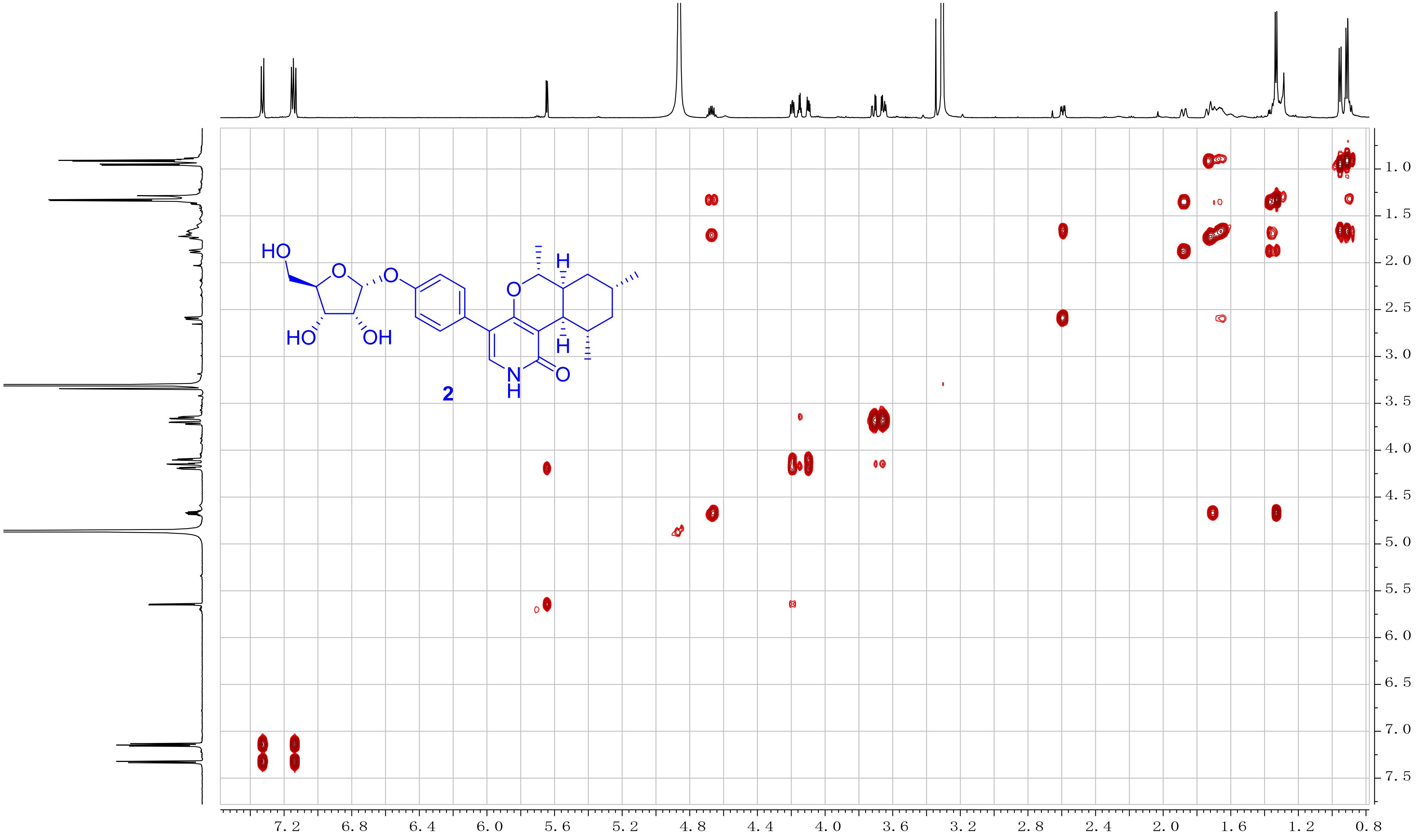

Figure S27. The ^{1}H-^{1}H COSY spectrum of compound **2** in CD3OD (600 MHz).

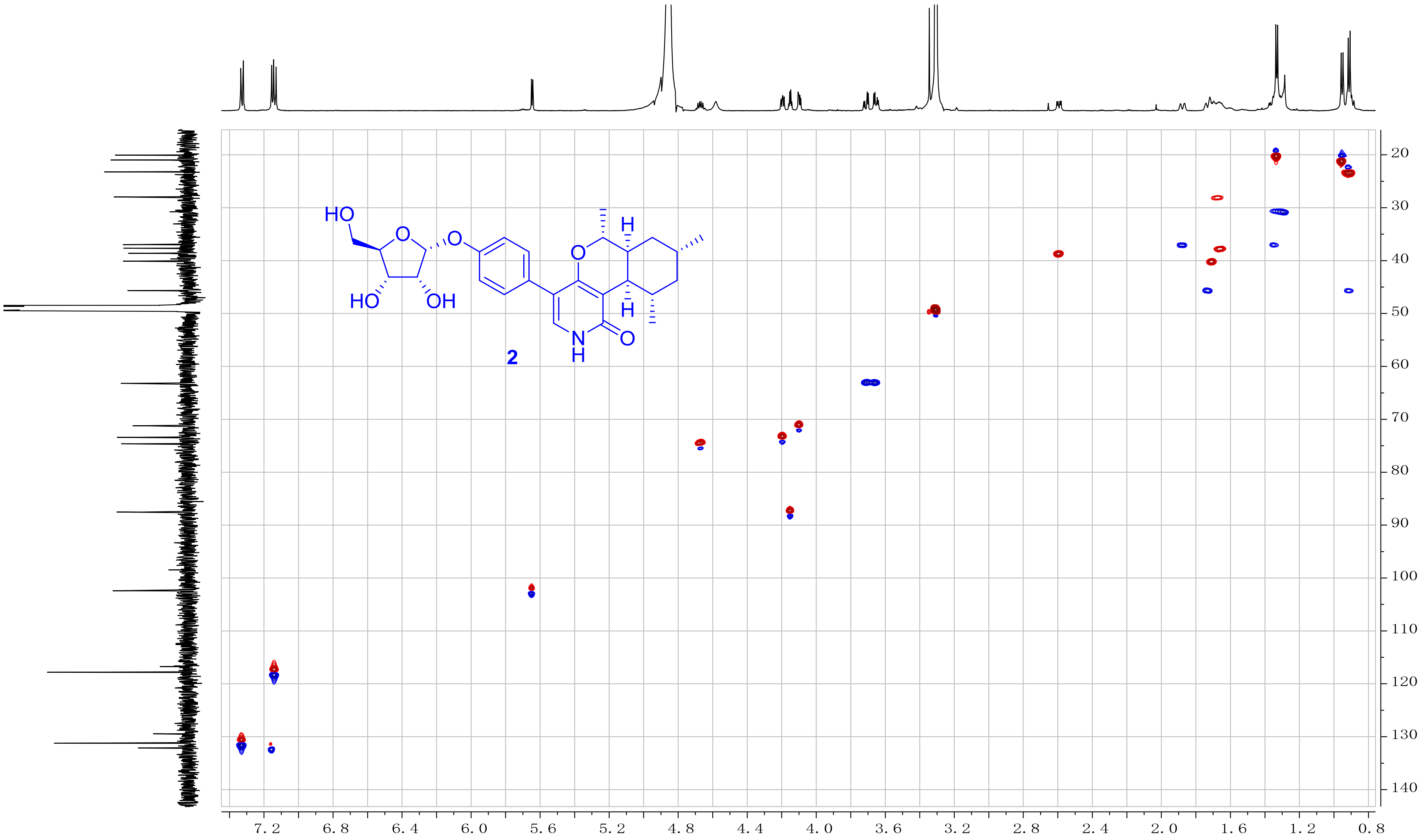

Figure S28. The HSQC spectrum of compound **2** in CD_3OD (600 MHz).

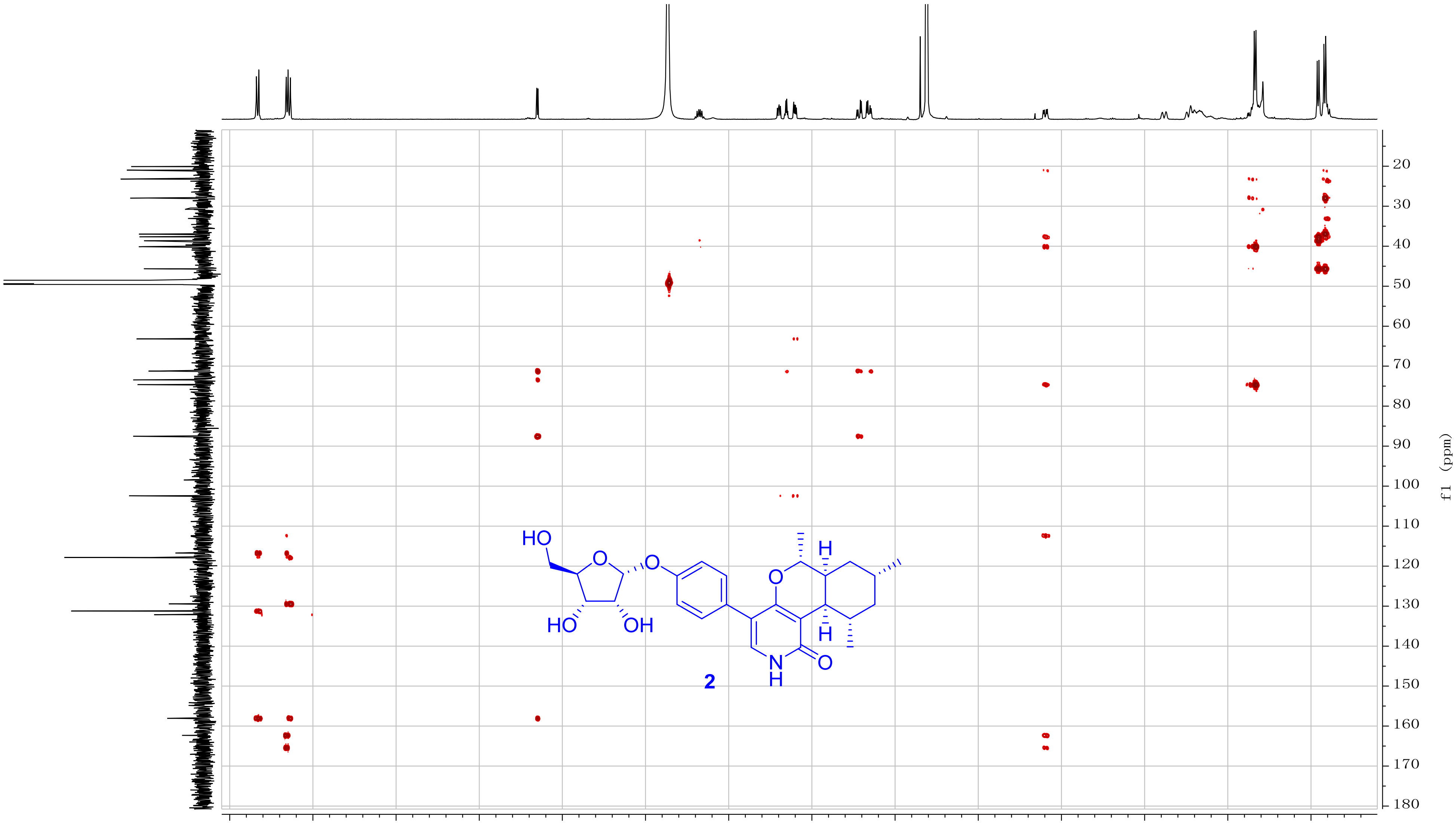

Figure S29. The HMBC spectrum of compound **2** in CD_3OD (600 MHz).

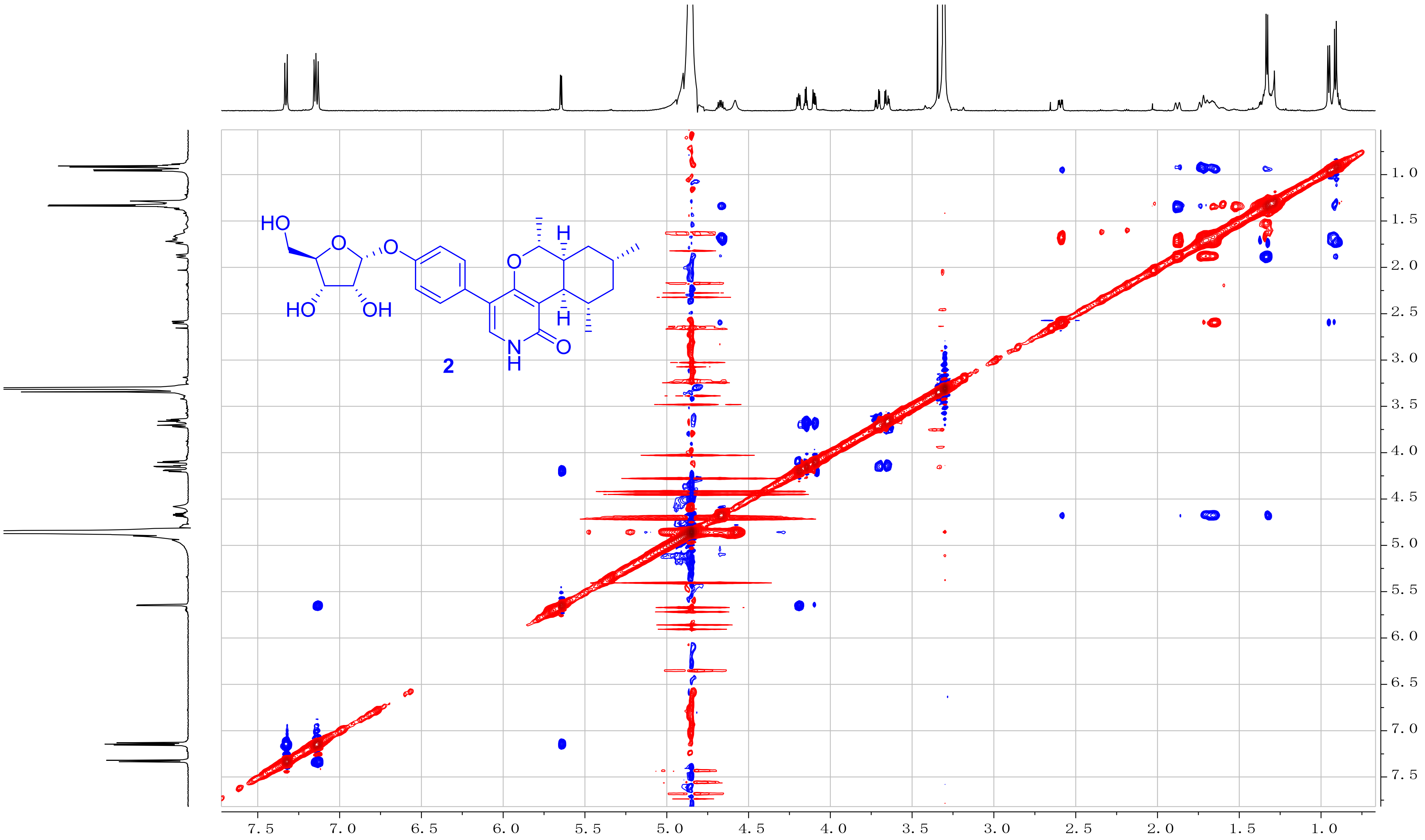

Figure S30. The ROESY spectrum of compound **2** in CD_3OD (600 MHz).

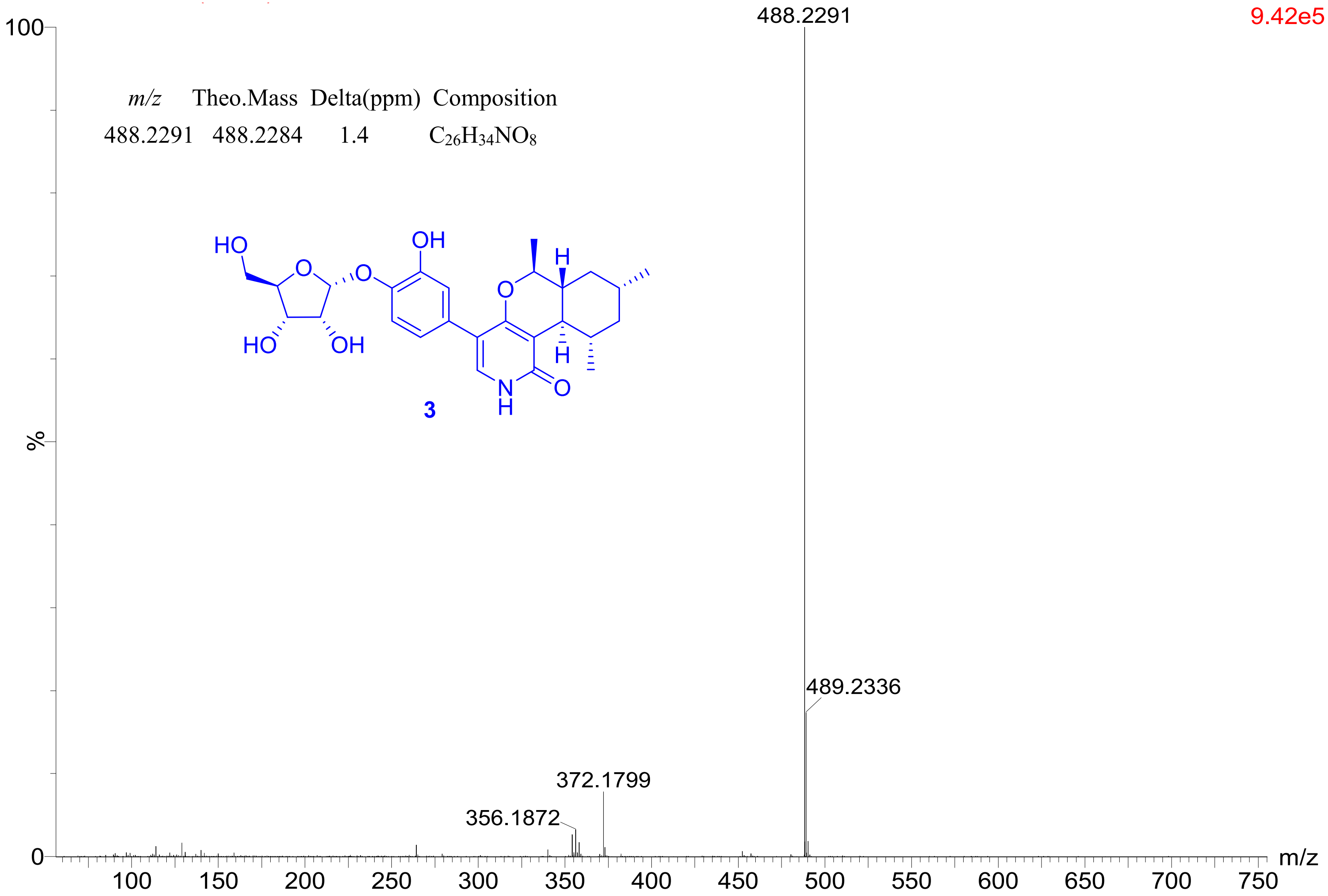

Figure S31. The (+)-HRESIMS spectrum of compound **3**.

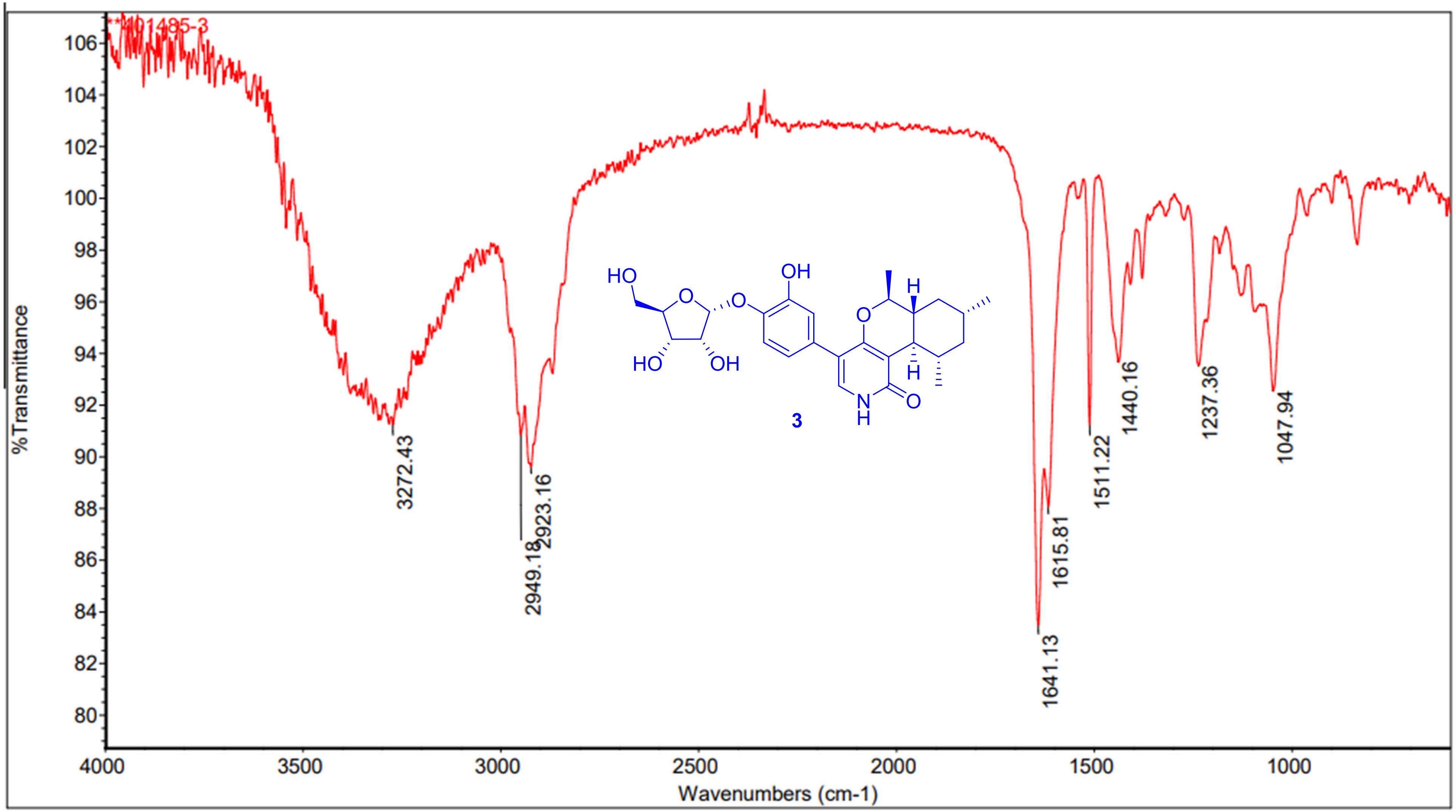

Figure S32. The IR spectrum of compound **3**.

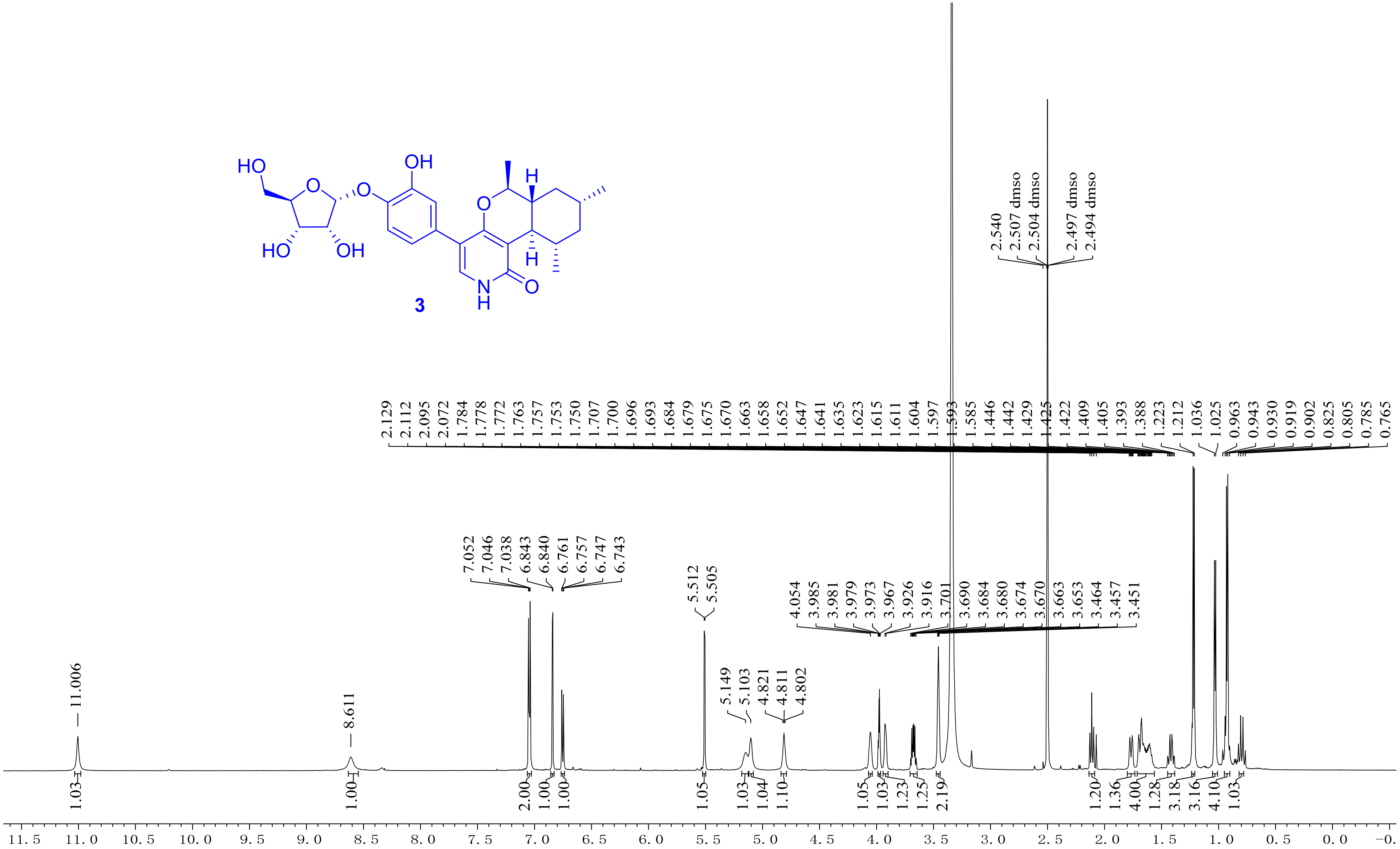

Figure S33. The ^{1}H NMR spectrum of compound **3** in DMSO-d_6 (600 MHz).

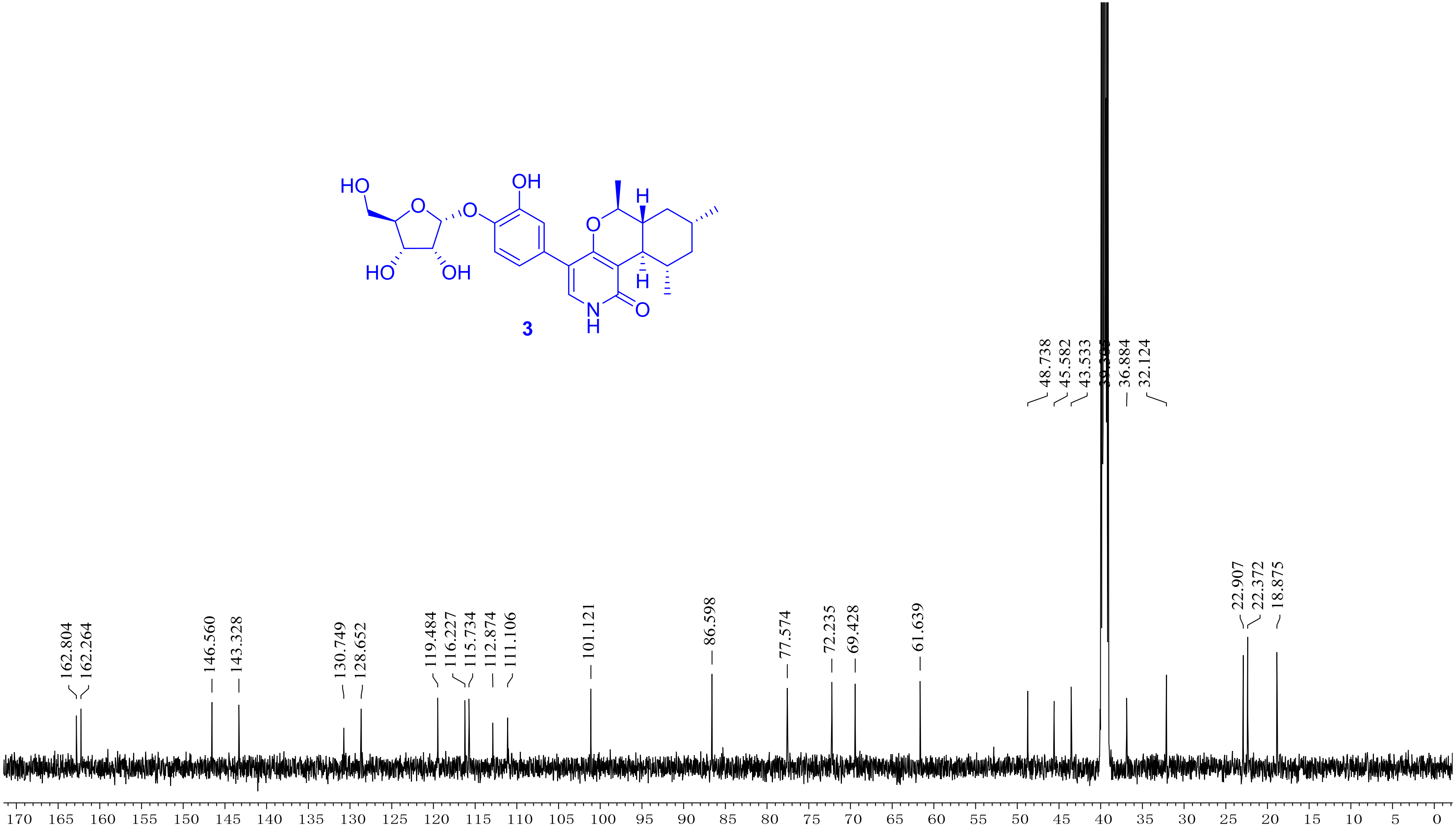

Figure S34. The ^{13}C NMR spectrum of compound **3** in DMSO-d_6 (150 MHz).

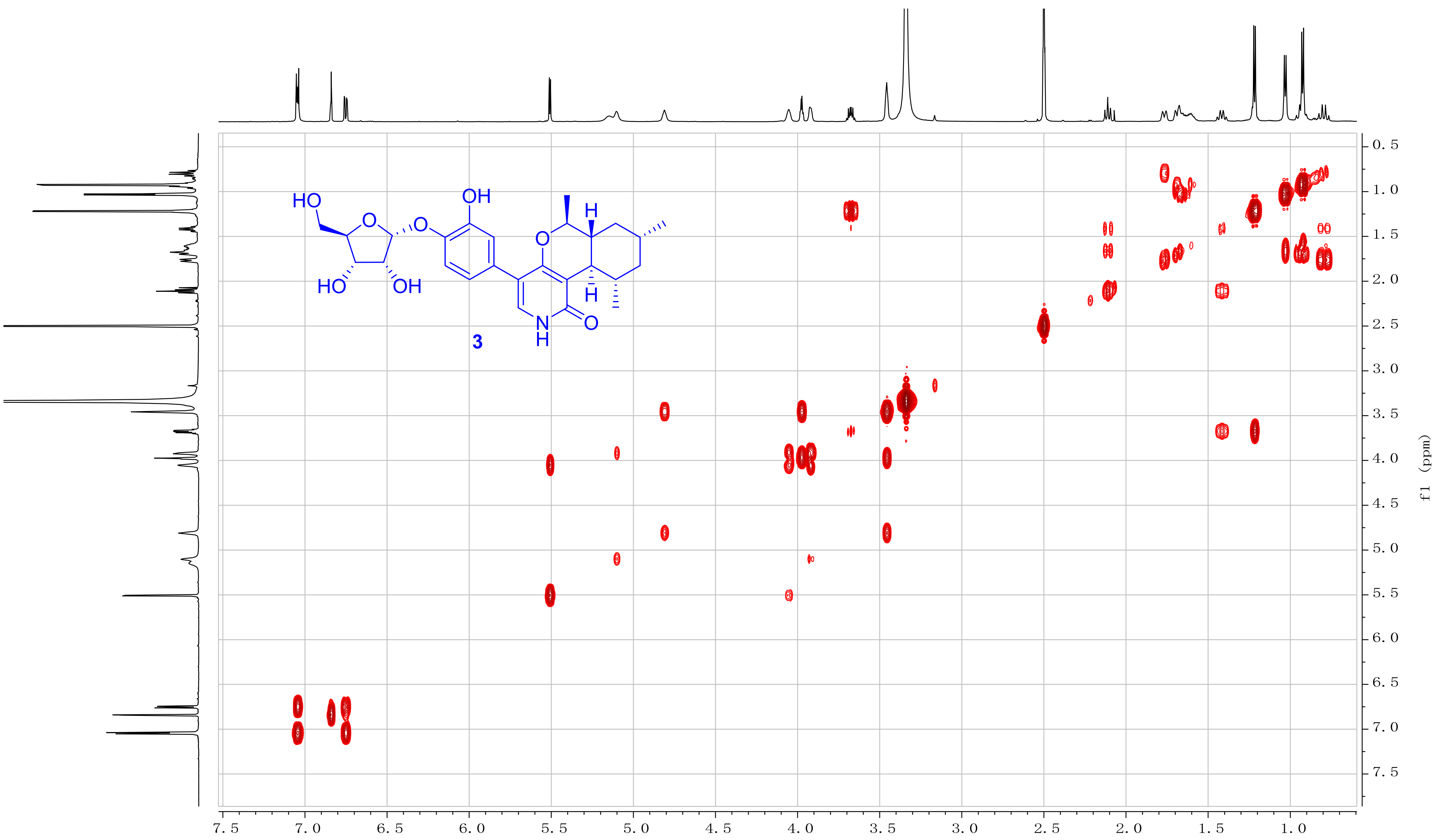

Figure S35. The ^{1}H-^{1}H COSY spectrum of compound **3** in DMSO-d_6 (600 MHz).

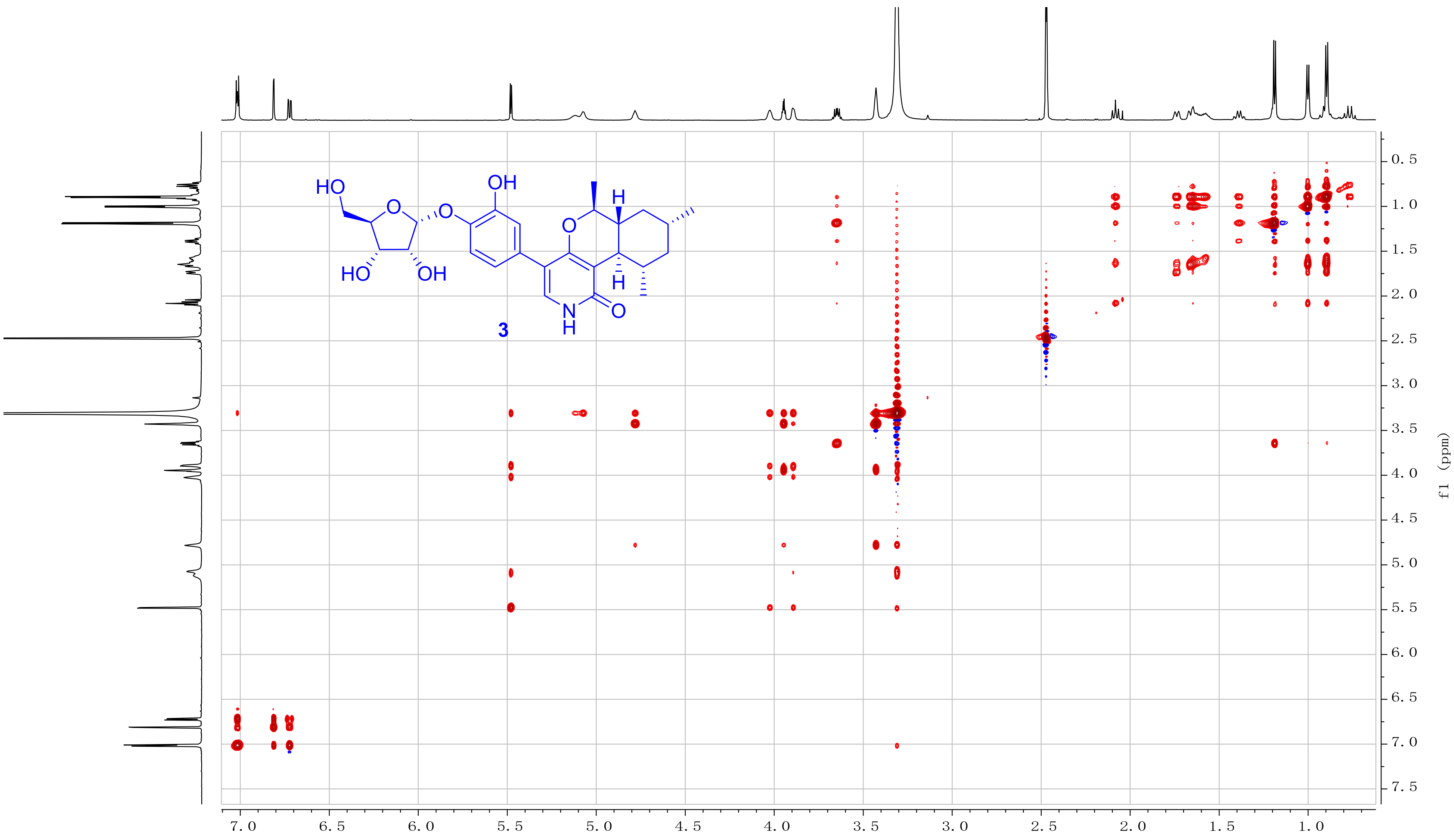

Figure S36. The TOCSY spectrum of compound **3** in DMSO-d_6 (600 MHz).

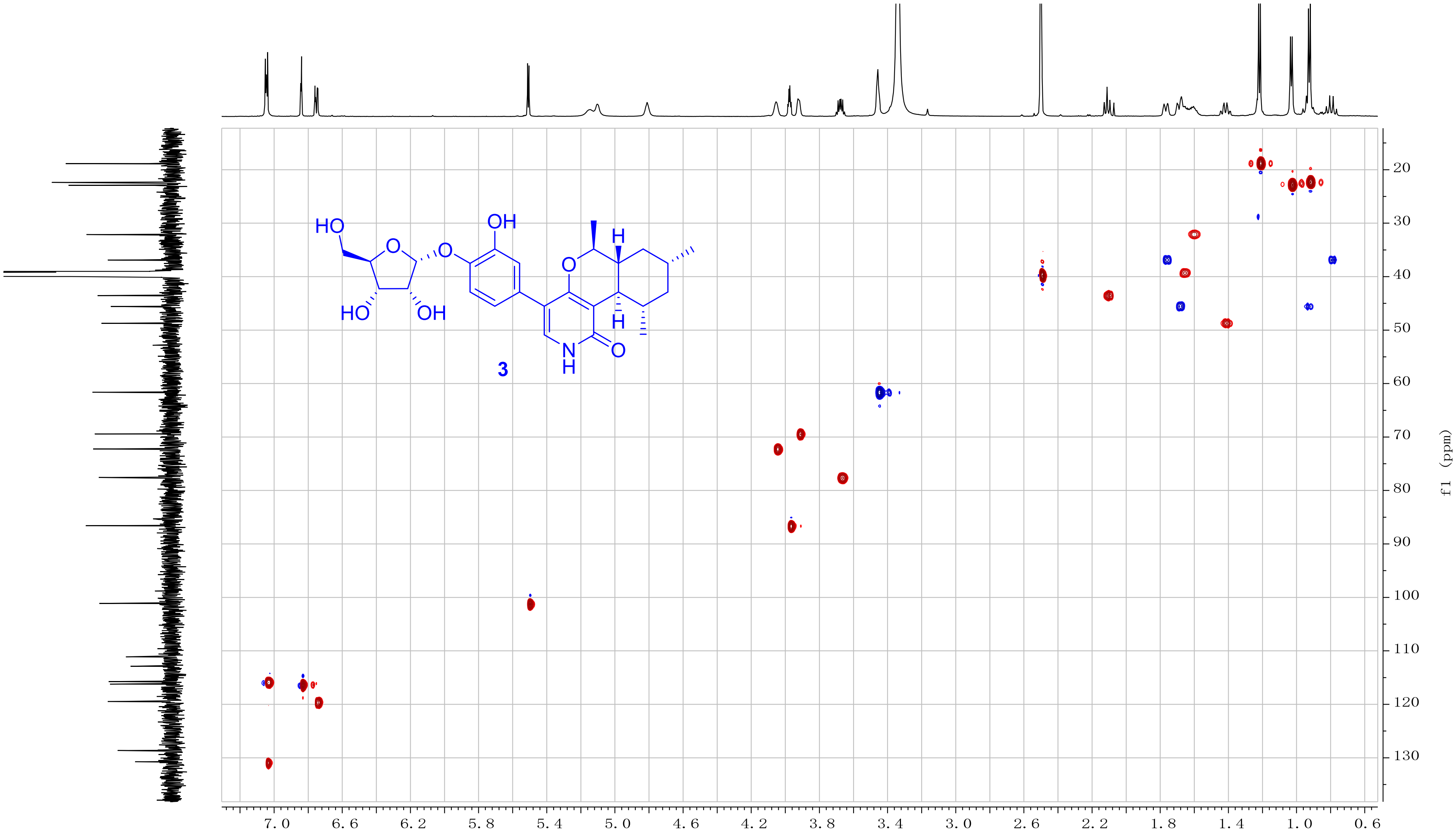

Figure S37. The HSQC spectrum of compound **3** in DMSO-d_6 (600 MHz).

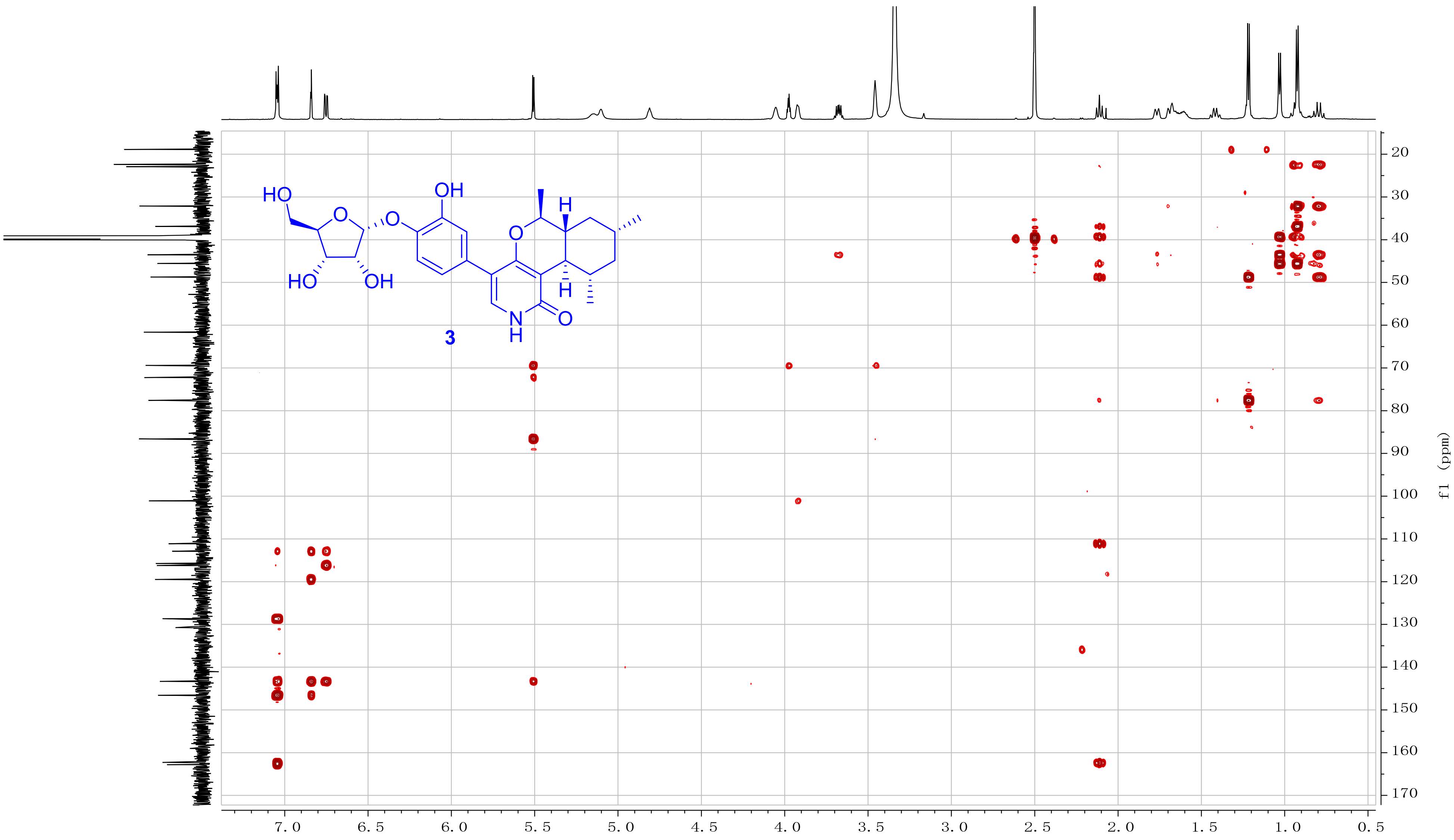

Figure S38. The HMBC spectrum of compound **3** in DMSO-d_6 (600 MHz).

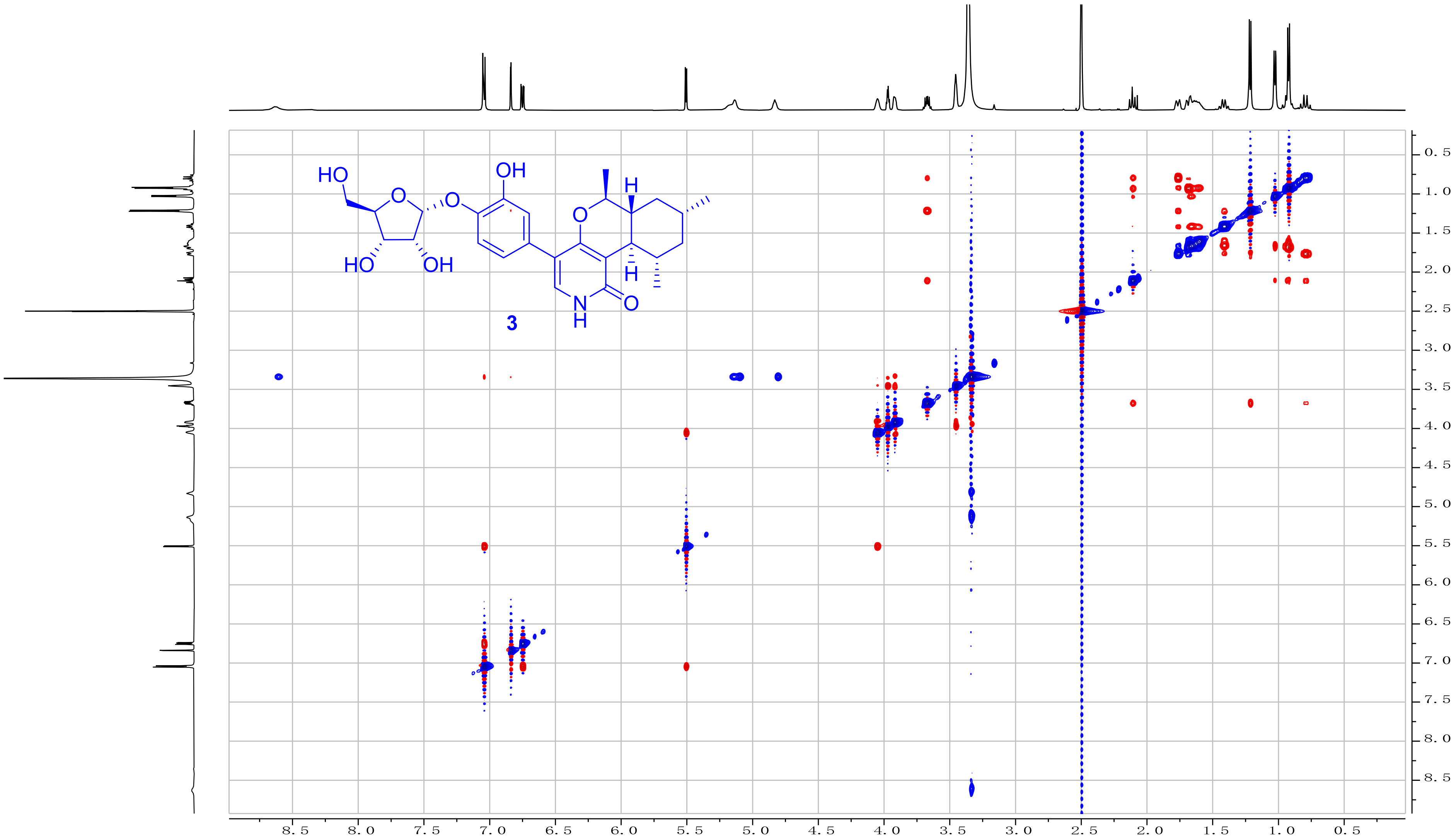

Figure S39. The ROESY spectrum of compound **3** in DMSO-d_6 (600 MHz).

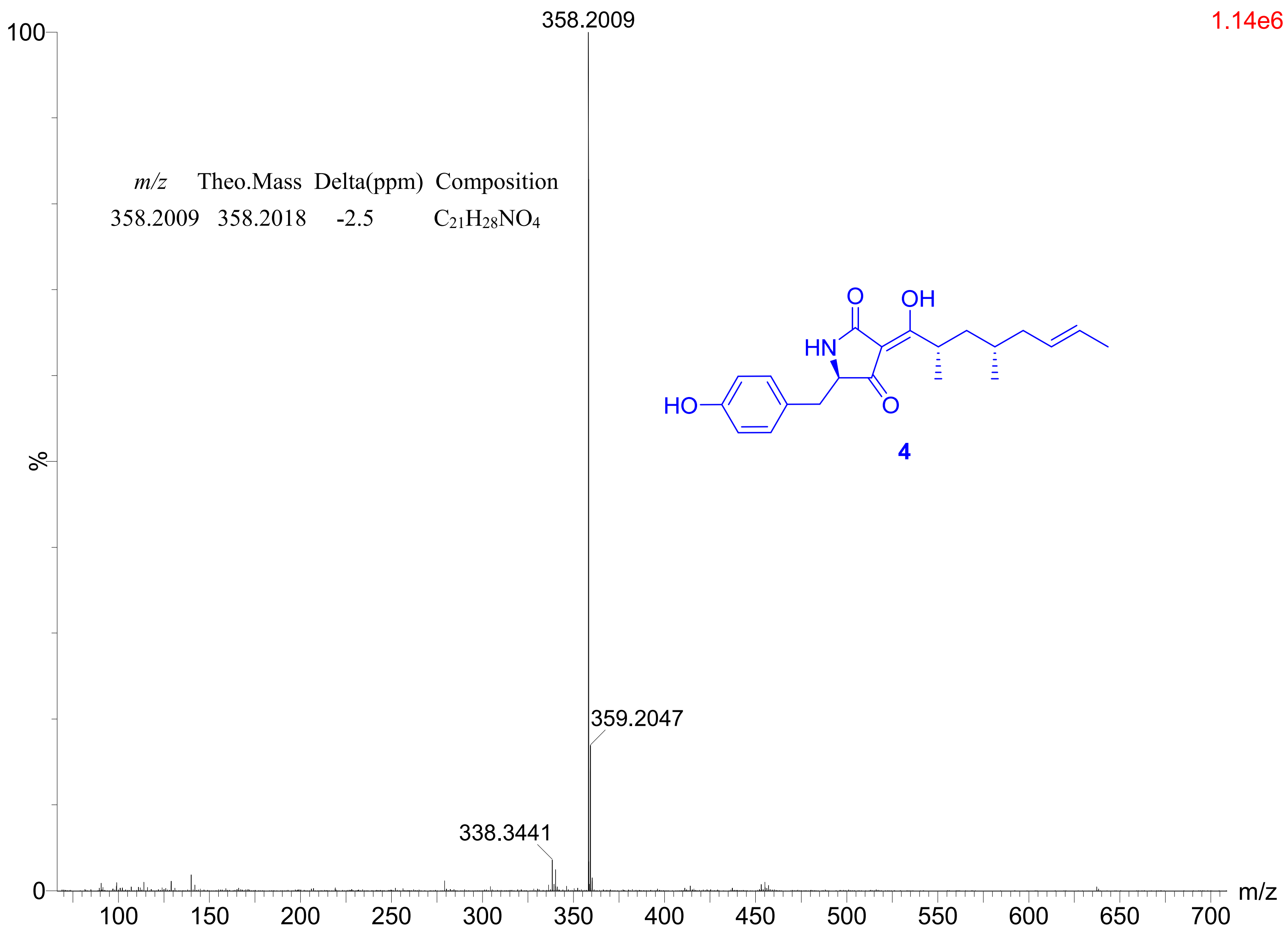

Figure S40. The (+)-HRESIMS spectrum of compound **4**.

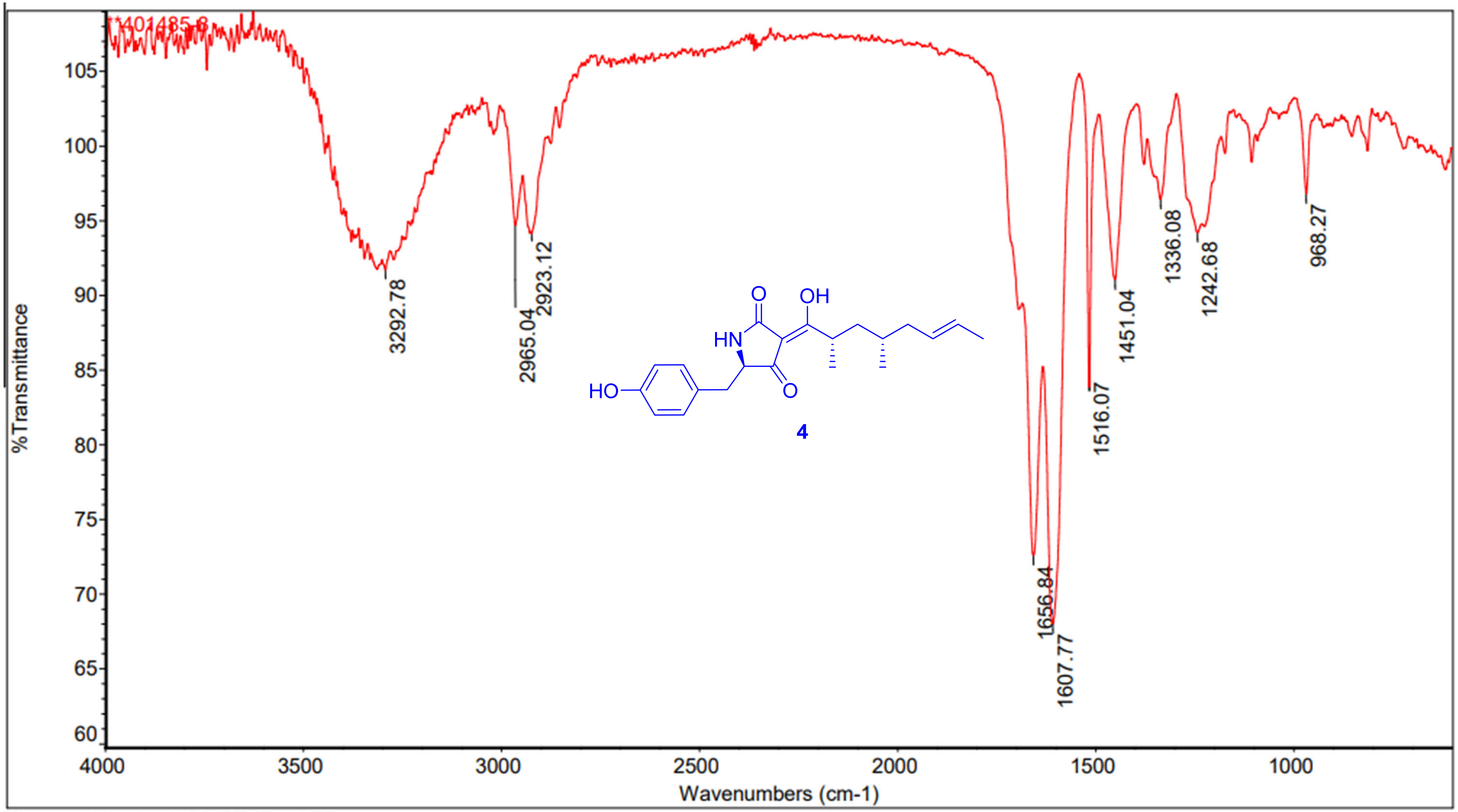

Figure S41. The IR spectrum of compound **4**.

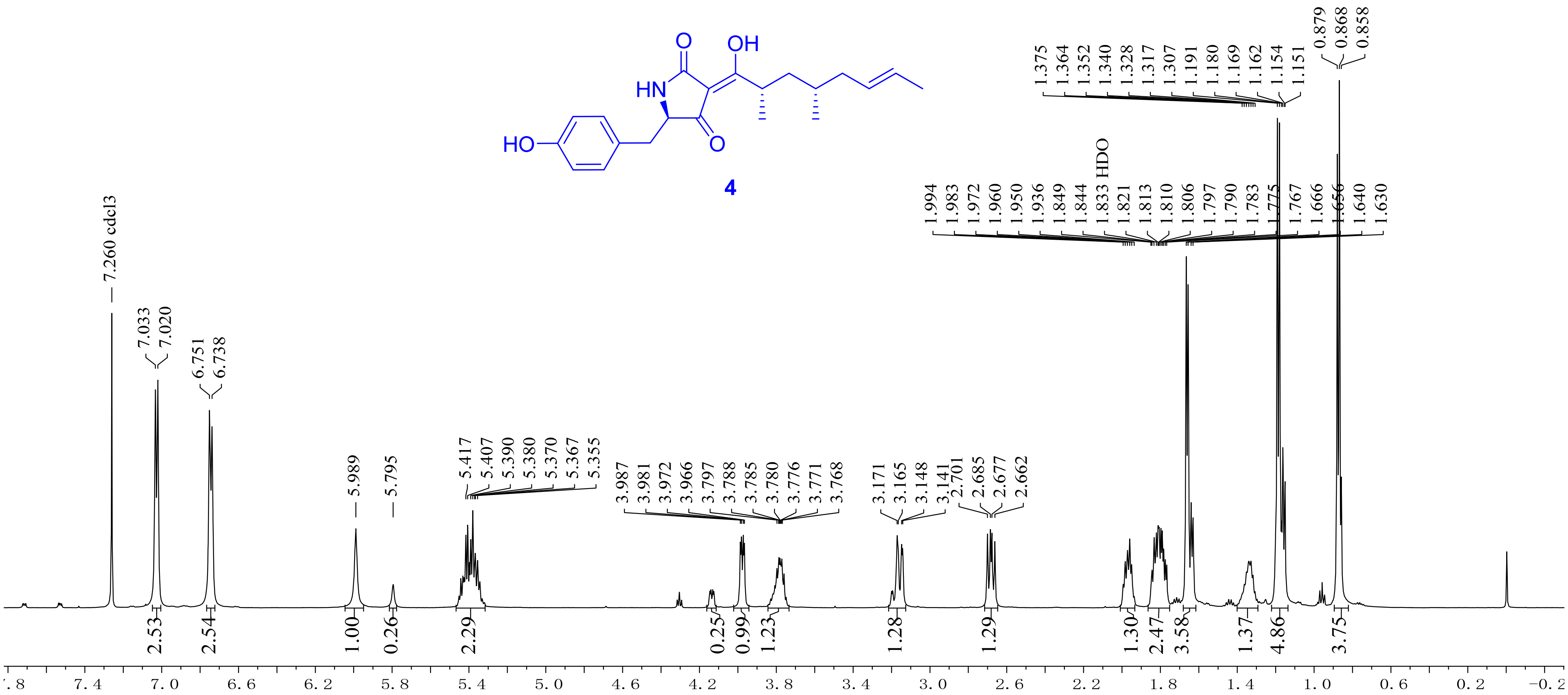

Figure S42. The ^{1}H NMR spectrum of compound **4** in $CDCl_3$ (600 MHz).

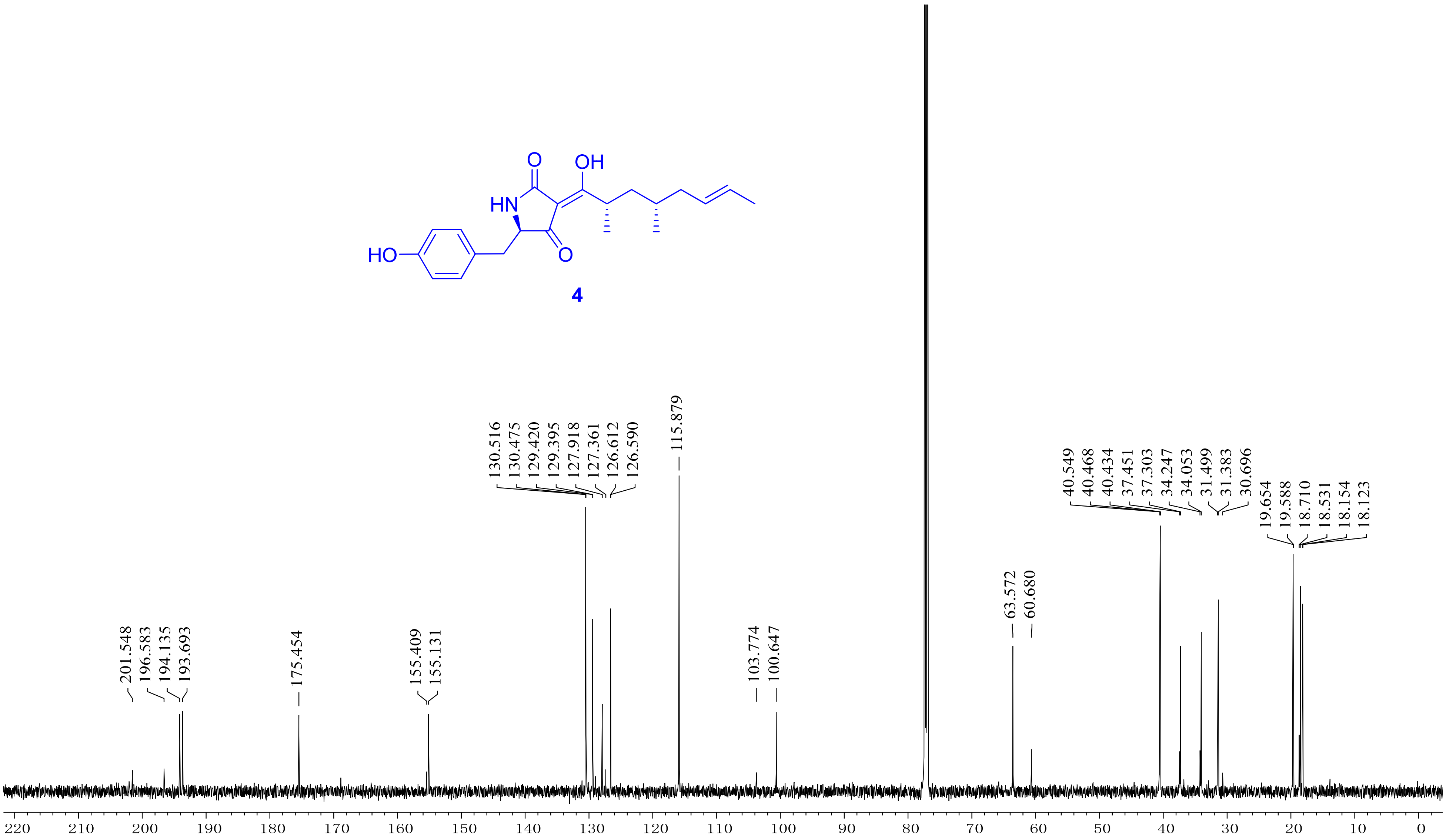

Figure S43. The ^{13}C NMR spectrum of compound **4** in $CDCl_3$ (150 MHz).

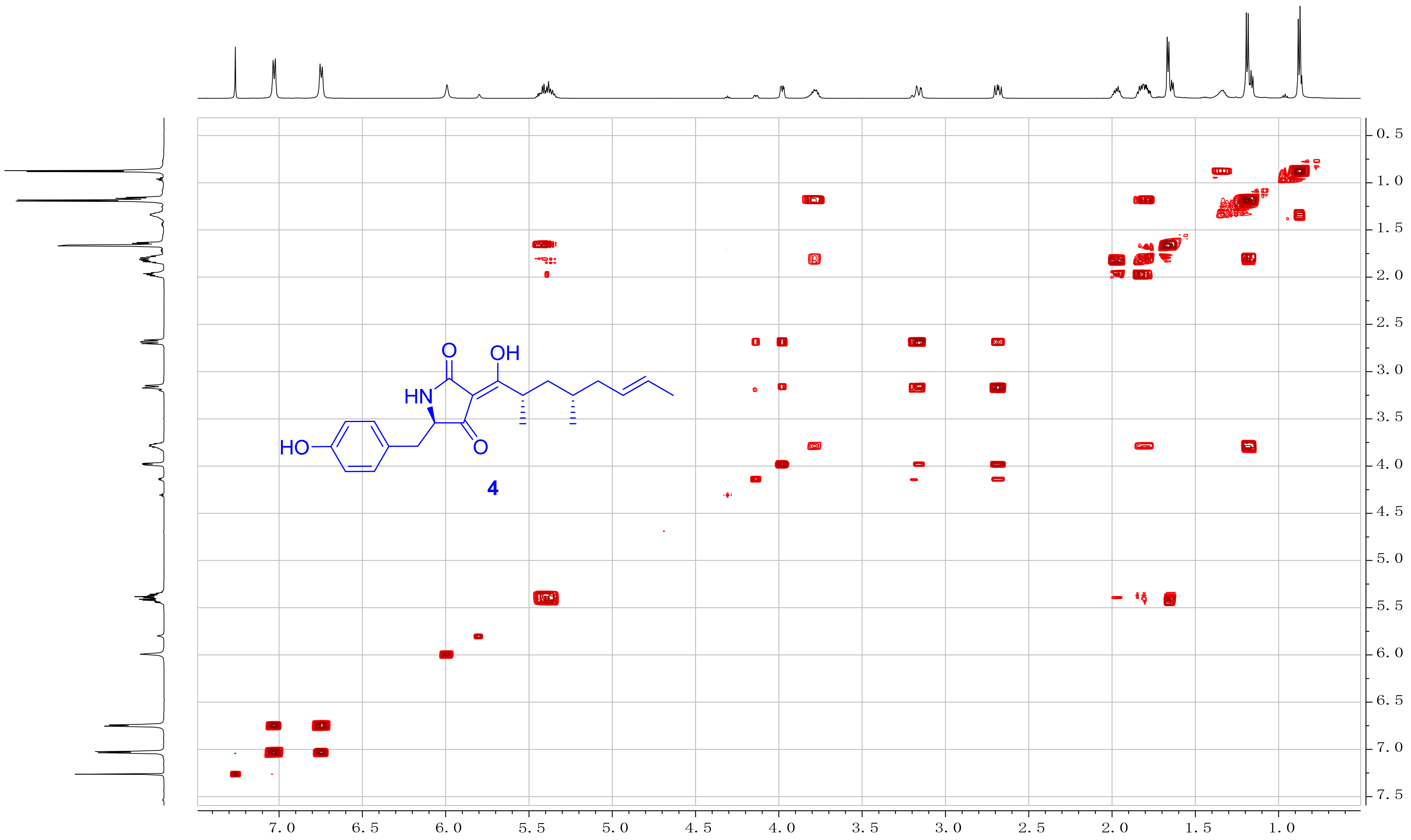

Figure S44. The ^{1}H-^{1}H COSY spectrum of compound **4** in $CDCl_3$ (600 MHz).

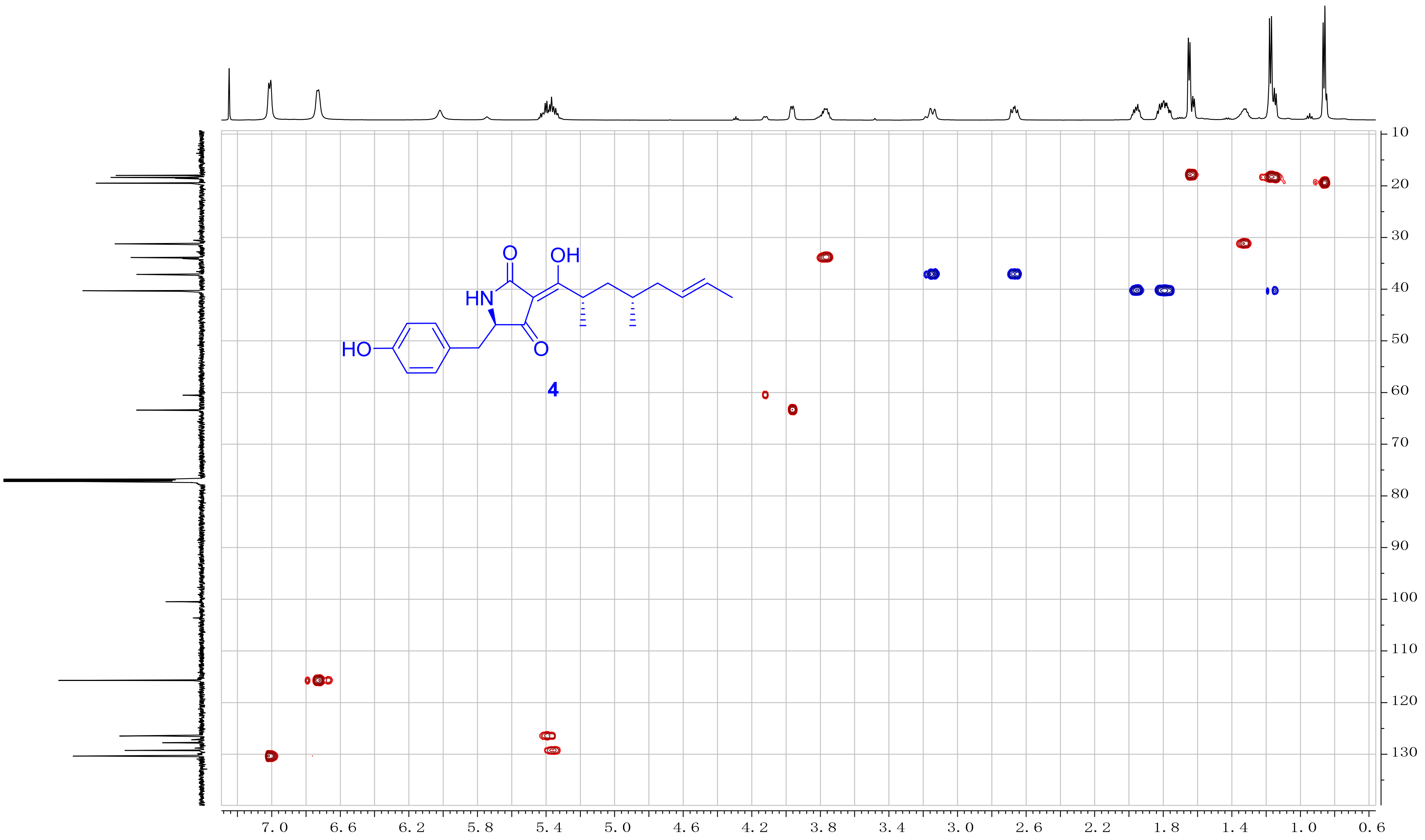

Figure S45. The HSQC spectrum of compound **4** in $CDCl_3$ (600 MHz).

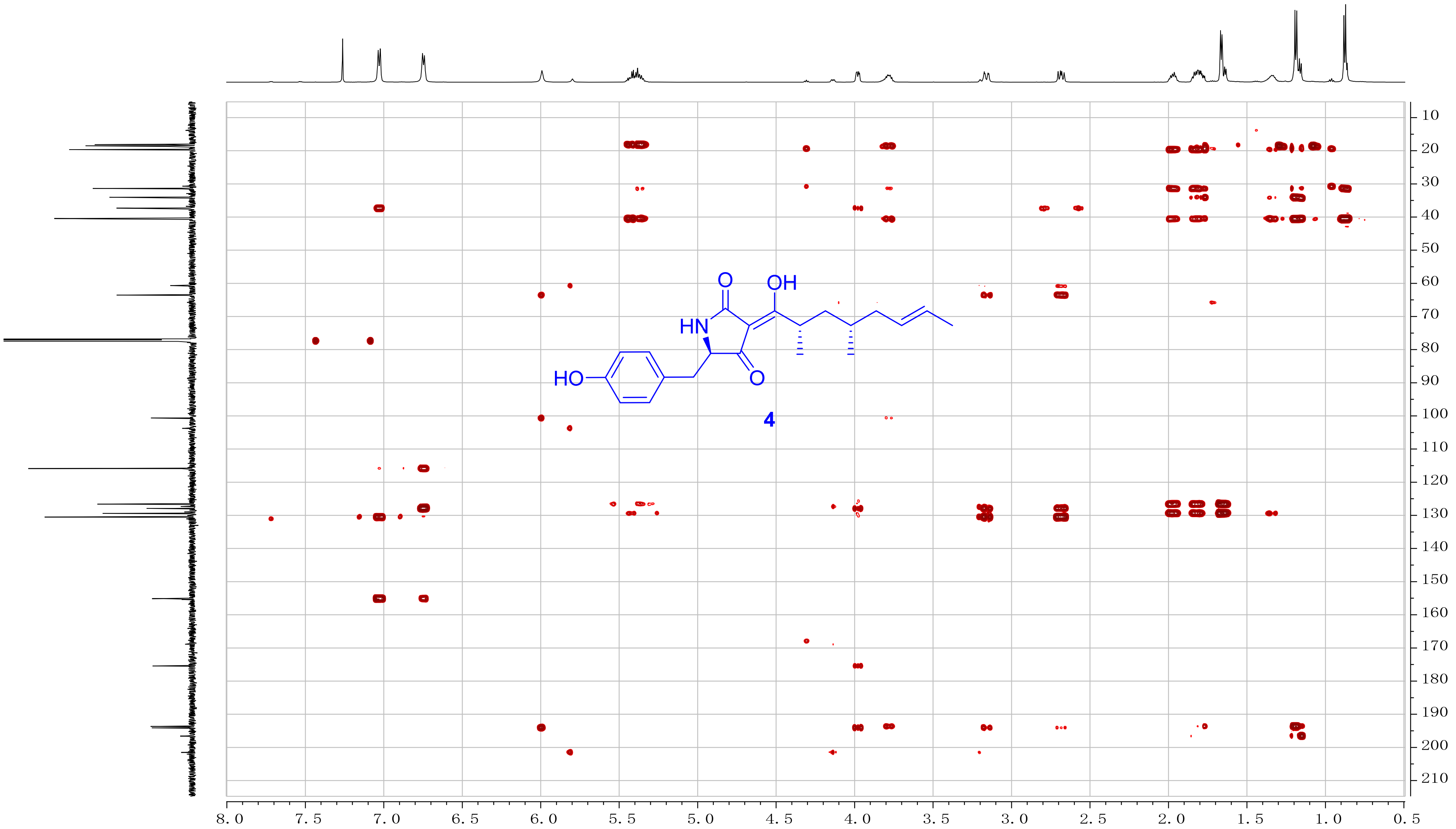

Figure S46. The HMBC spectrum of compound **4** in $CDCl_3$ (600 MHz).

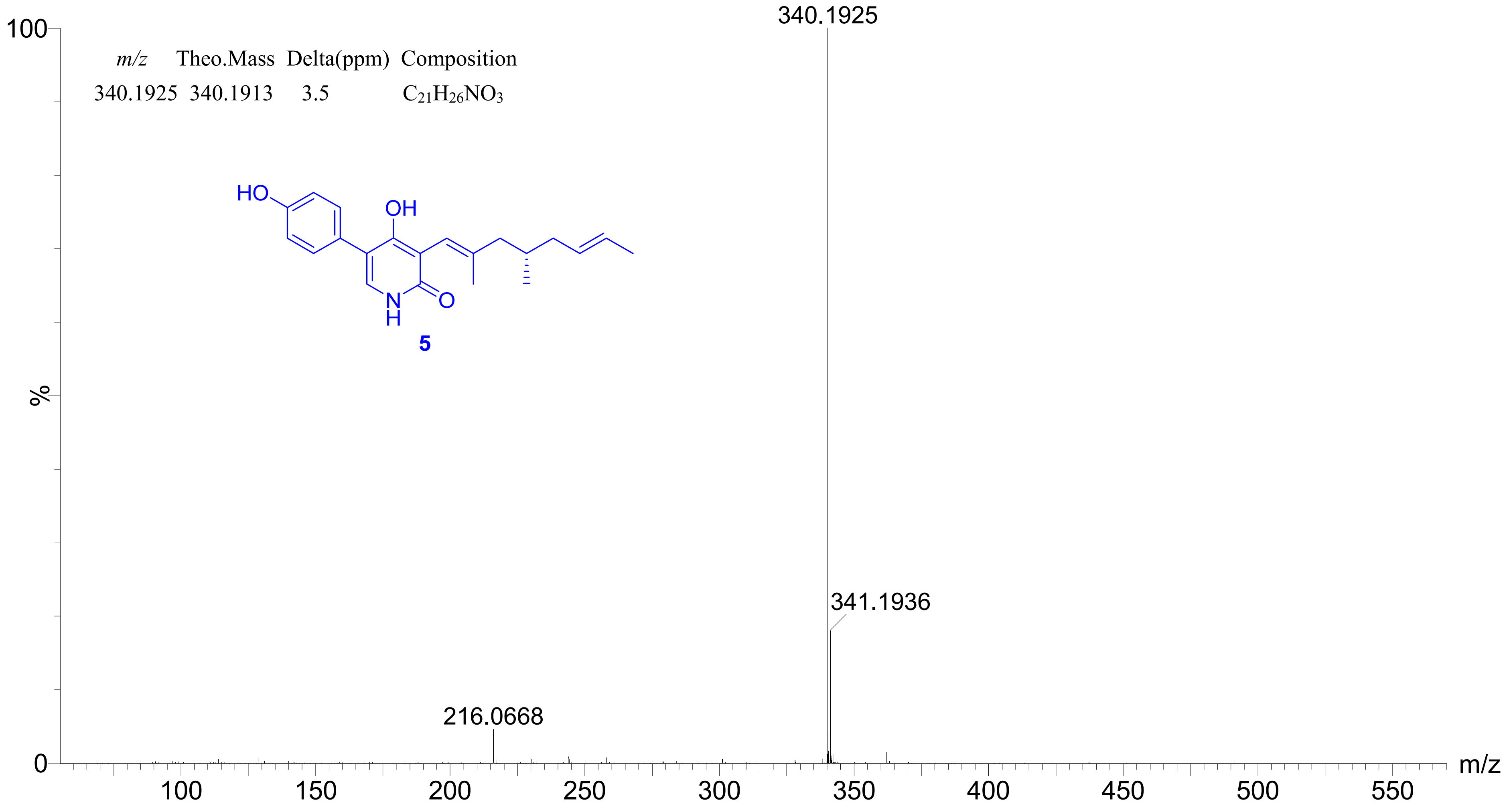

Figure S47. The (+)-HRESIMS spectrum of compound **5**.

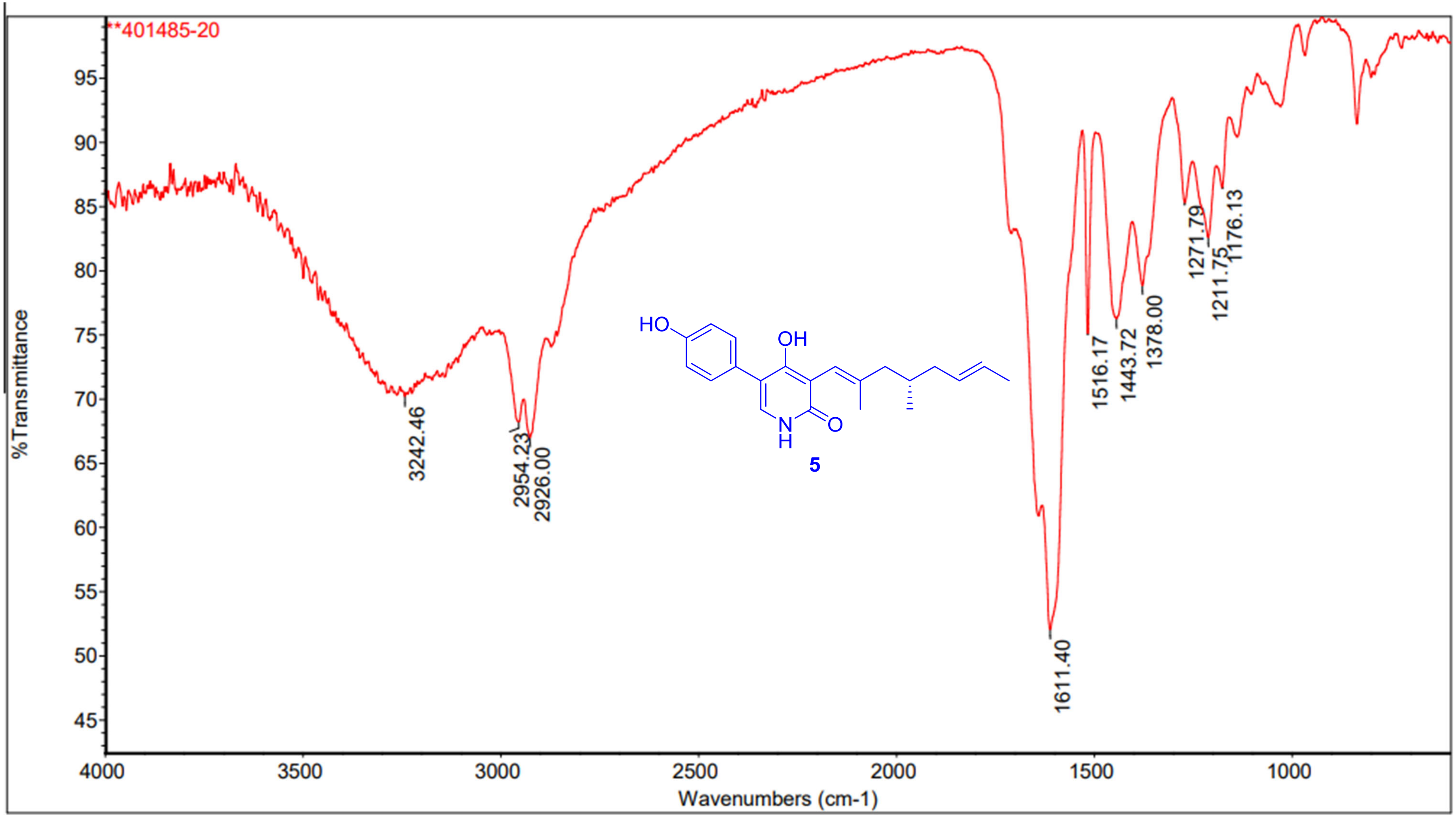

Figure S48. The IR spectrum of compound **5**.

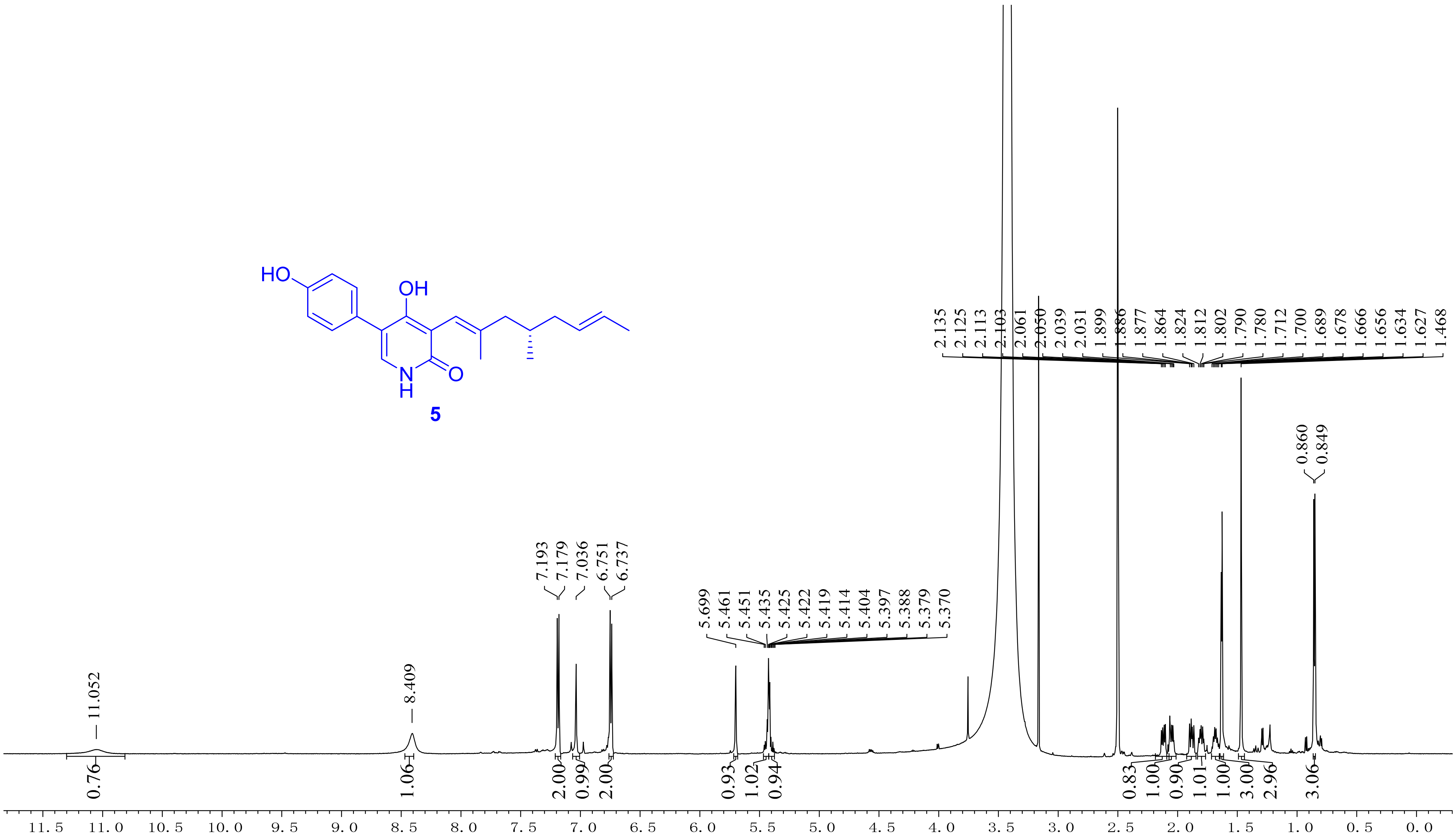

Figure S49. The ^{1}H NMR spectrum of compound **5** in DMSO-d_6 (600 MHz).

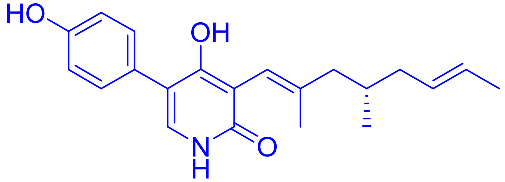

5

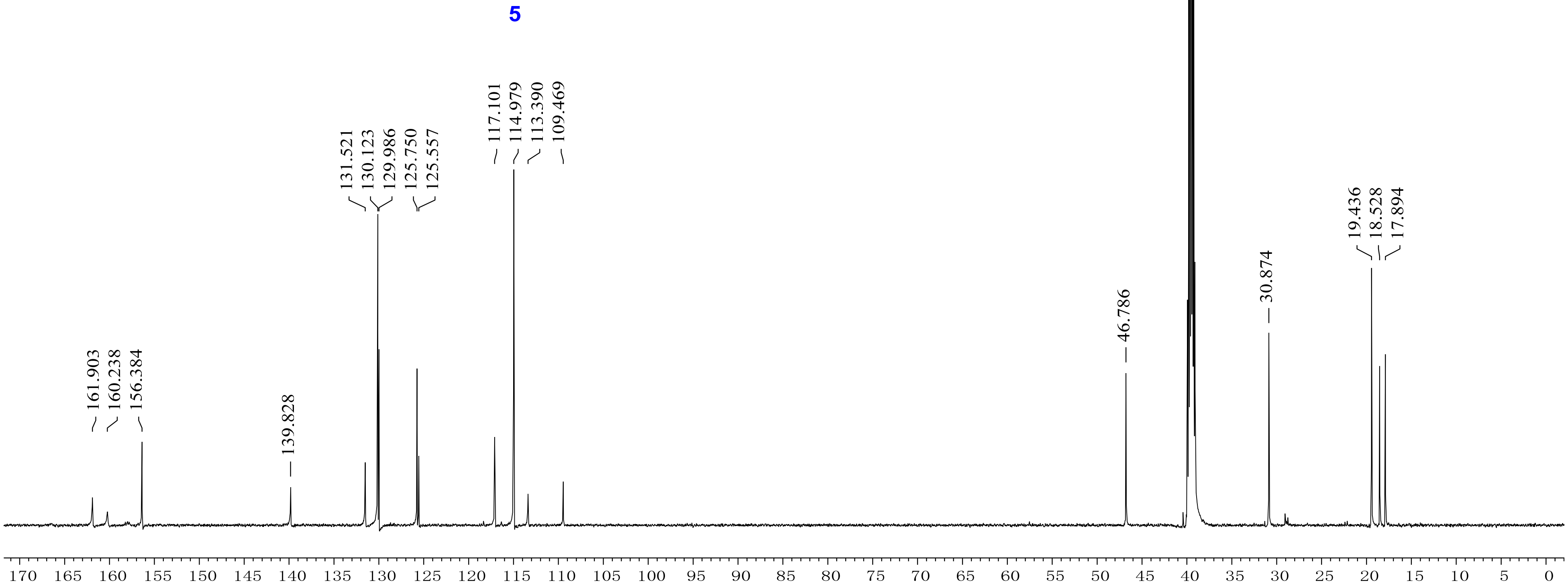

Figure S50. The ^{13}C NMR spectrum of compound **5** in DMSO-d_6 (150 MHz).

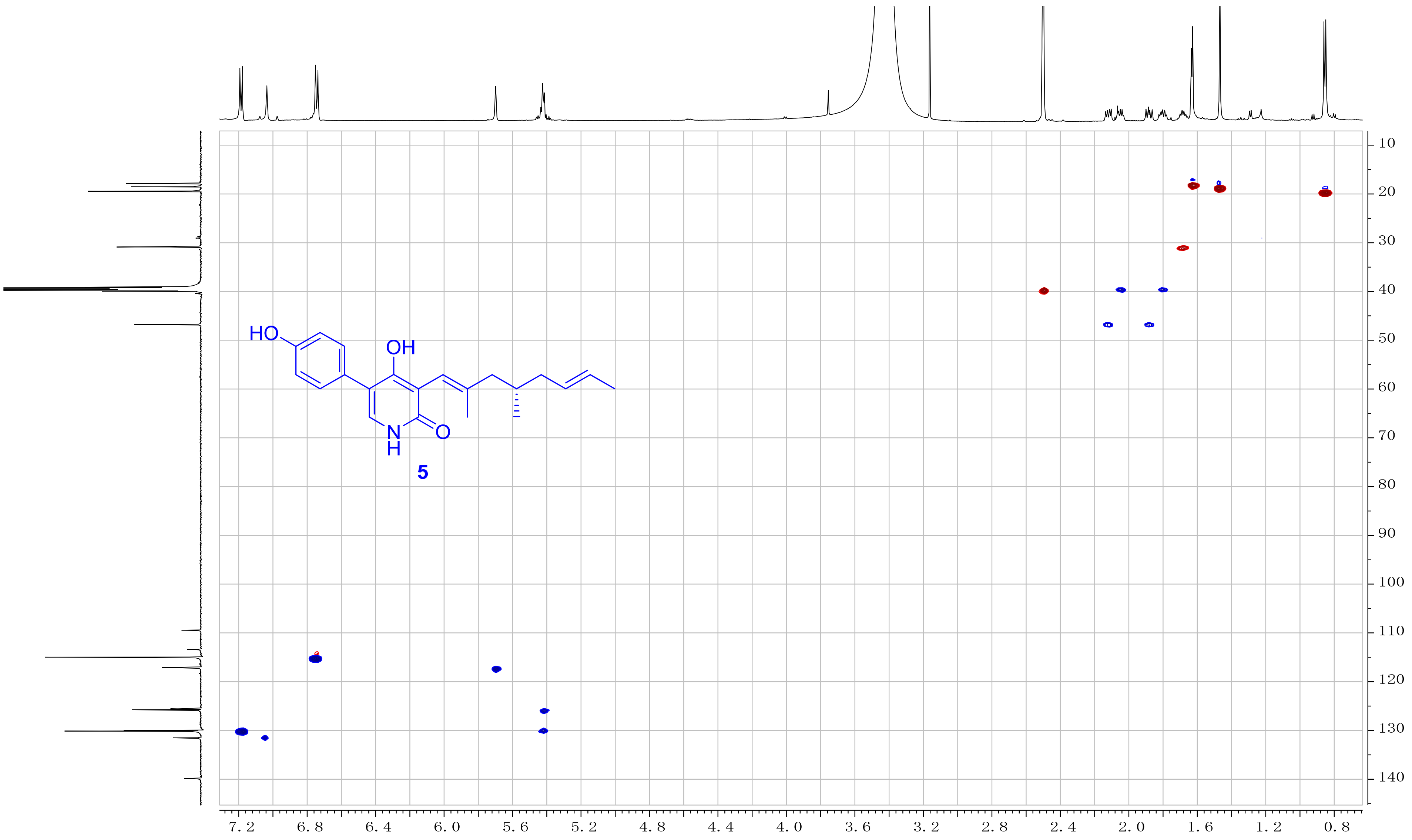

Figure S51. The HSQC spectrum of compound **5** in DMSO-d_6 (600 MHz).

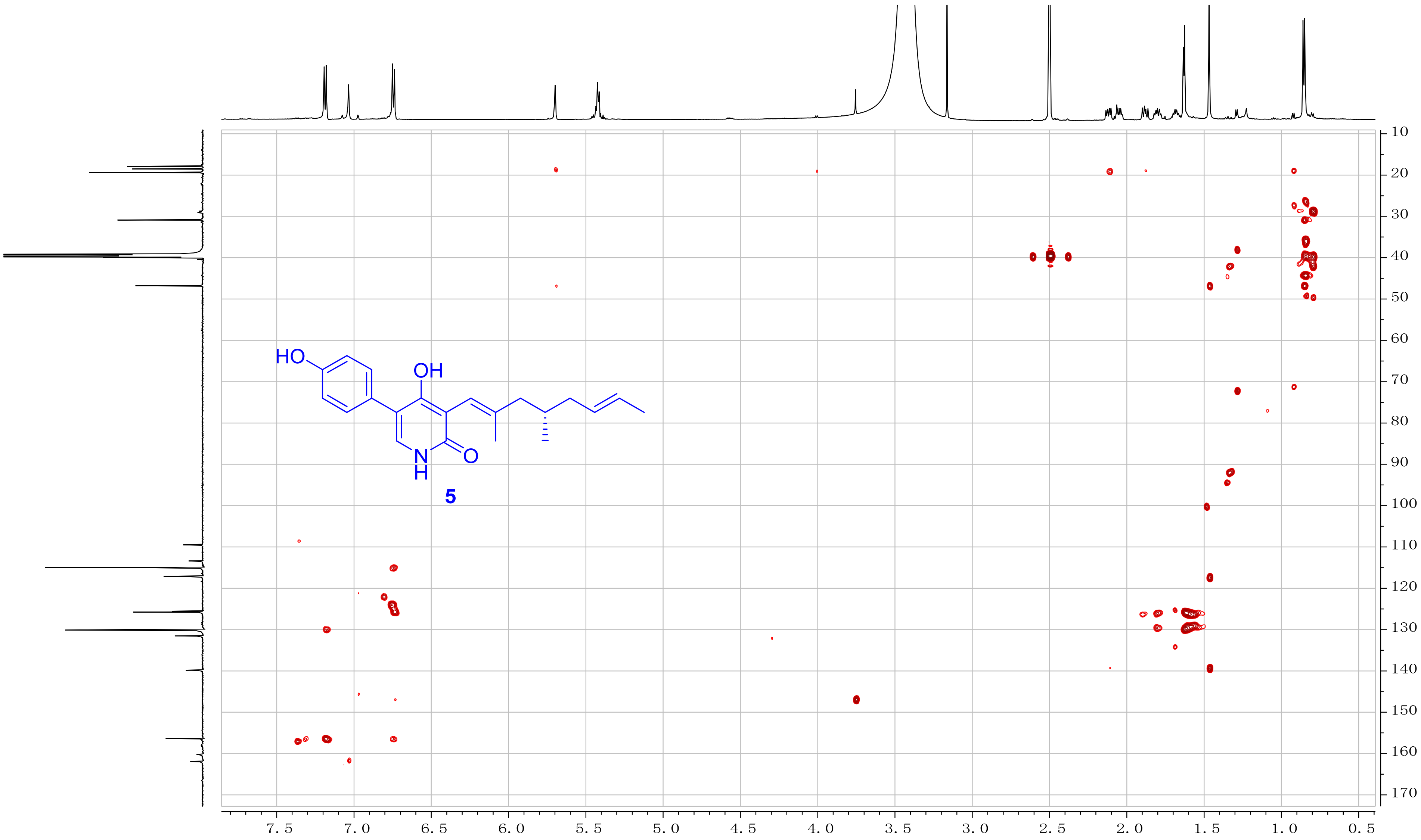

Figure S52. The HMBC spectrum of compound **5** in DMSO-d_6 (600 MHz).

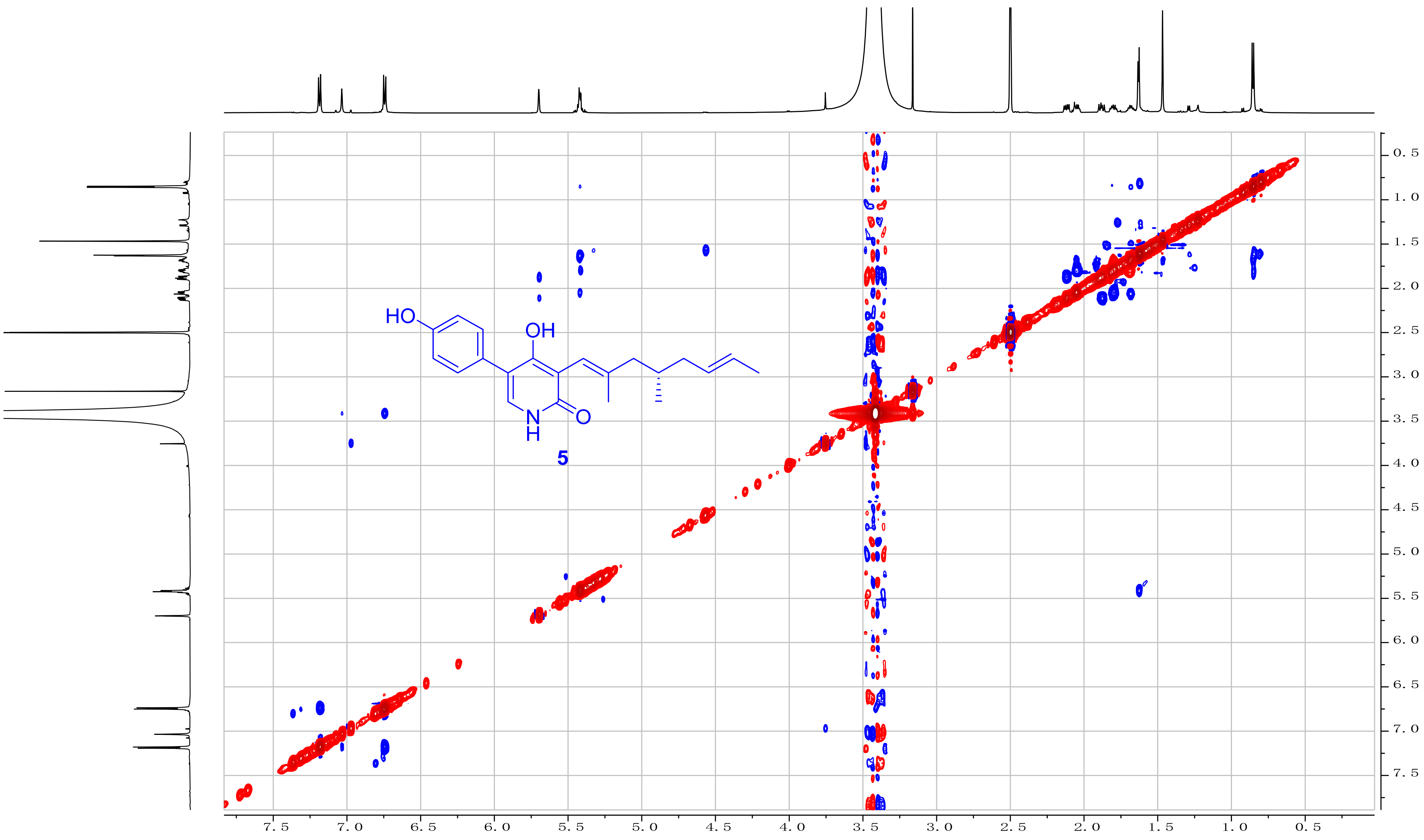

Figure S53. The ROESY spectrum of compound **5** in DMSO-d_6 (600 MHz).

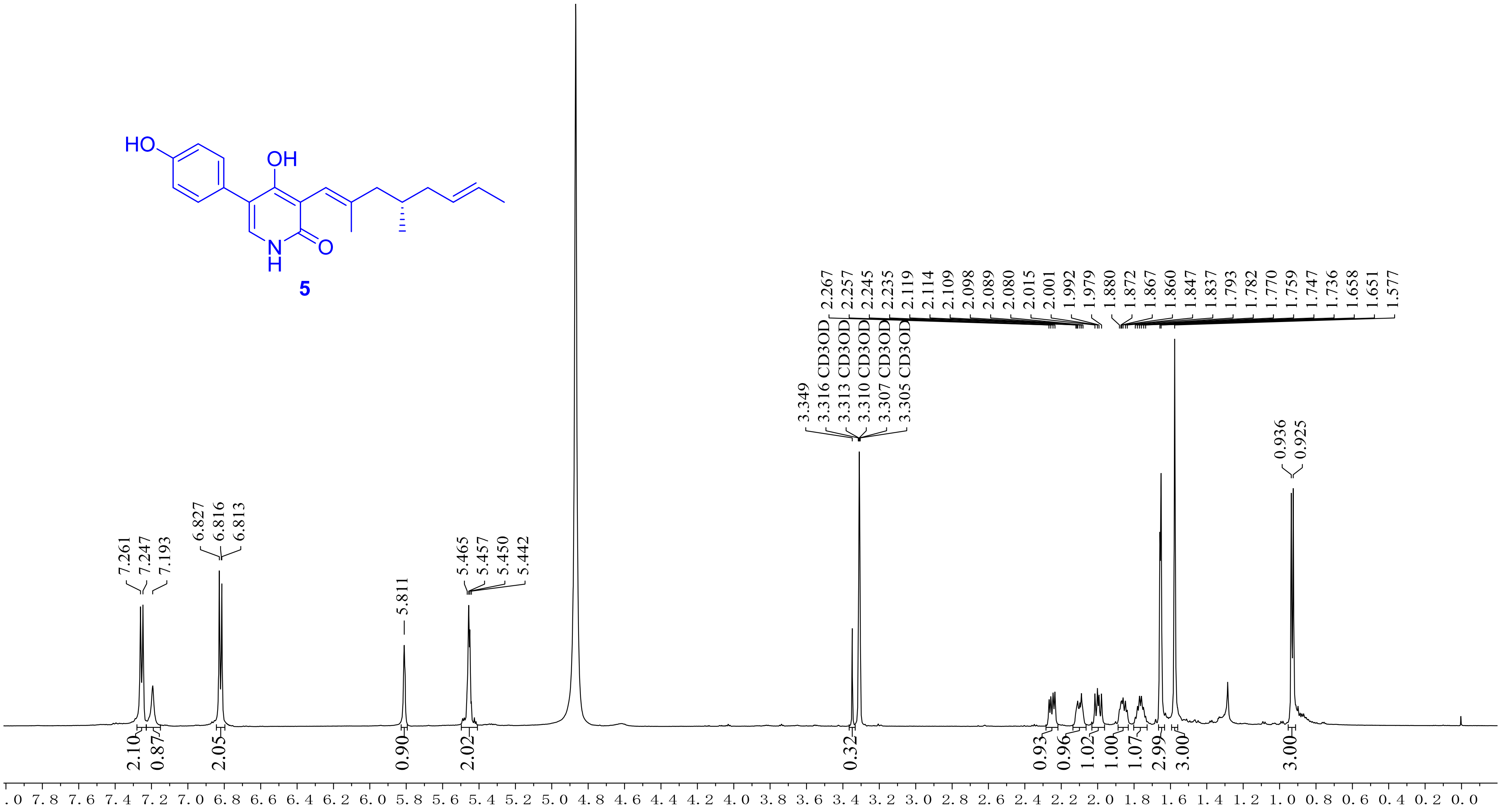

Figure S54. The ^{1}H NMR spectrum of compound **5** in CD_3OD (600 MHz).

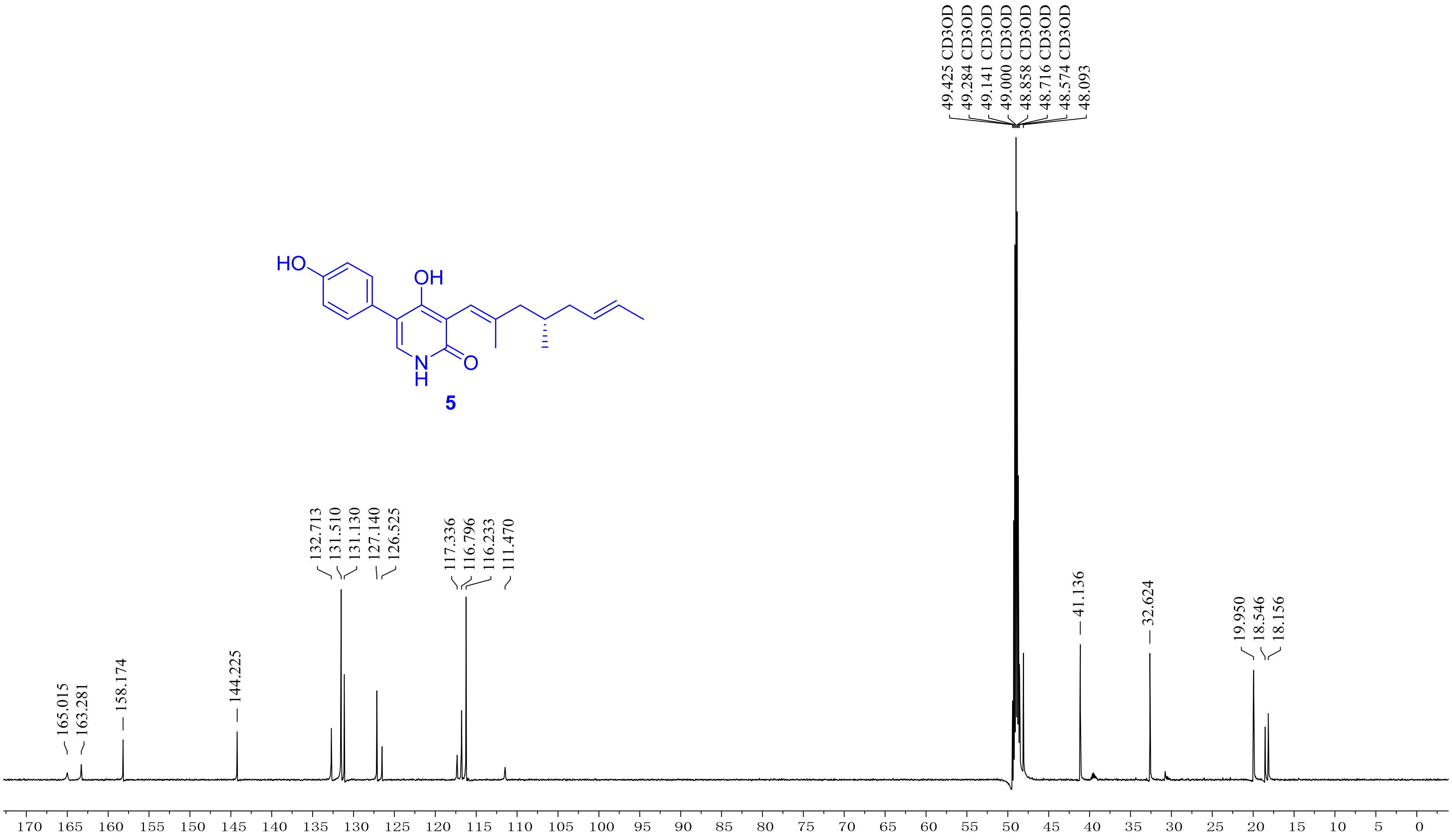

Figure S55. The ^{13}C NMR spectrum of compound **5** in CD_3OD (150 MHz).

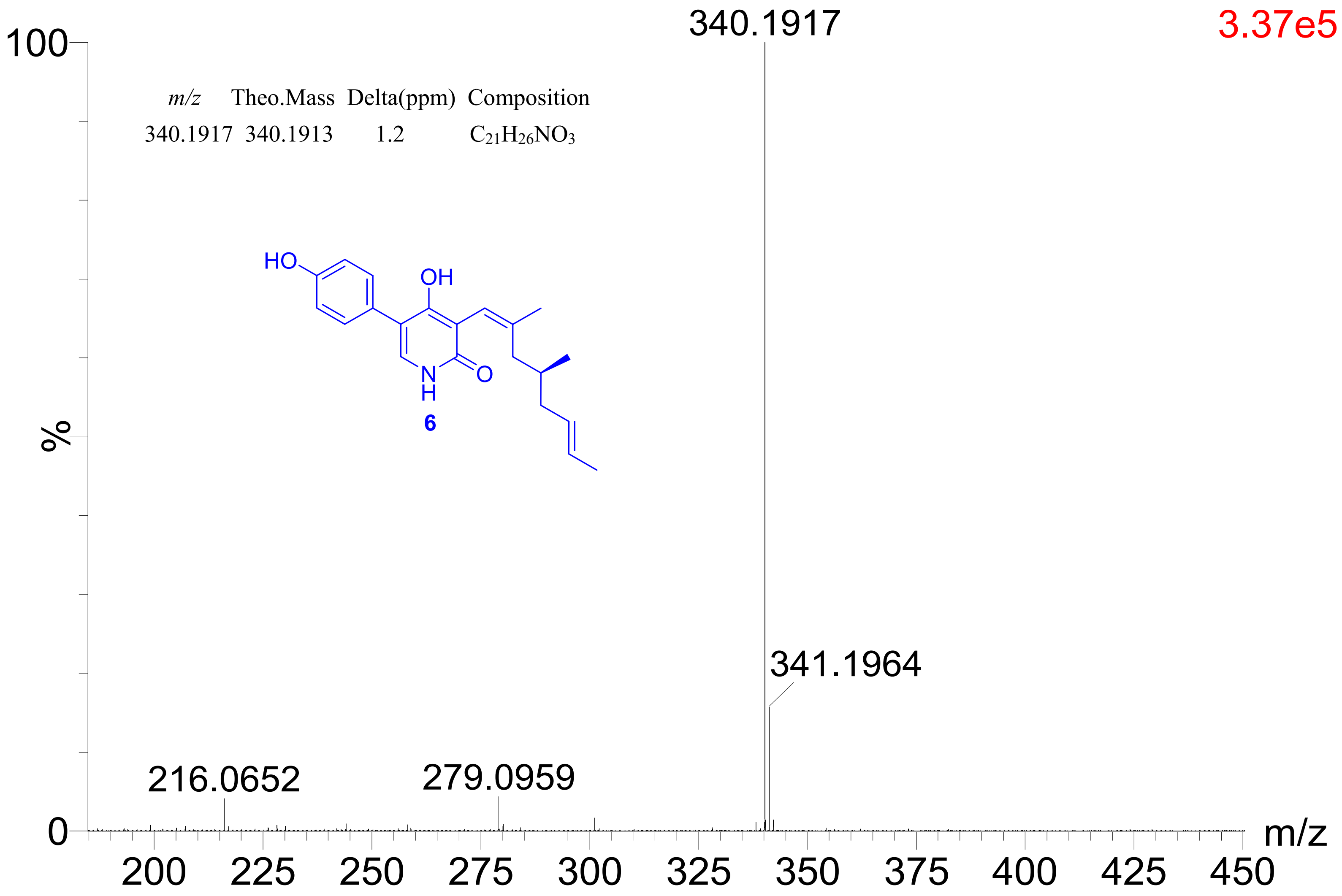

Figure S56. The (+)-HRESIMS spectrum of compound **6**.

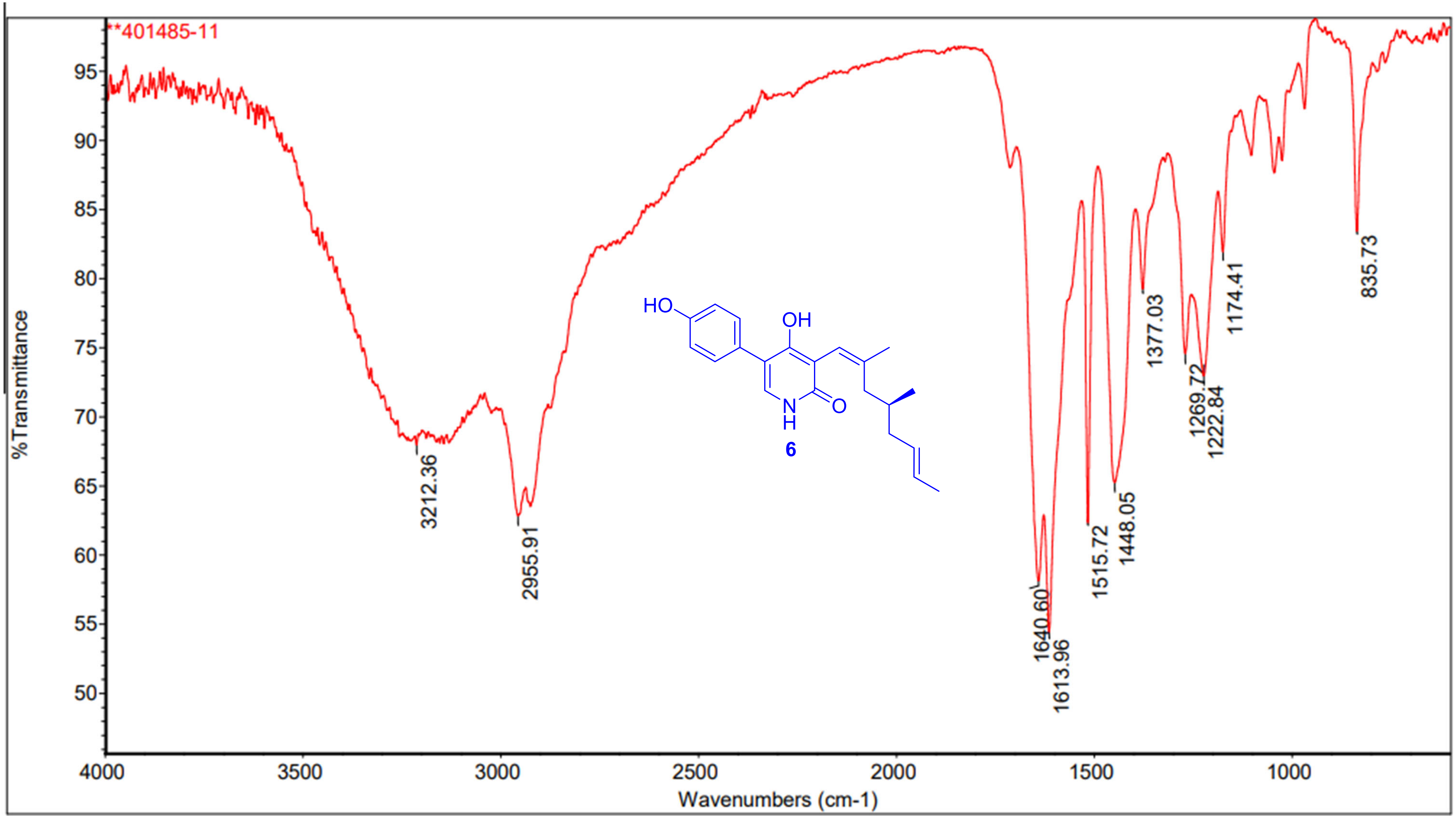

Figure S57. The IR spectrum of compound **6**.

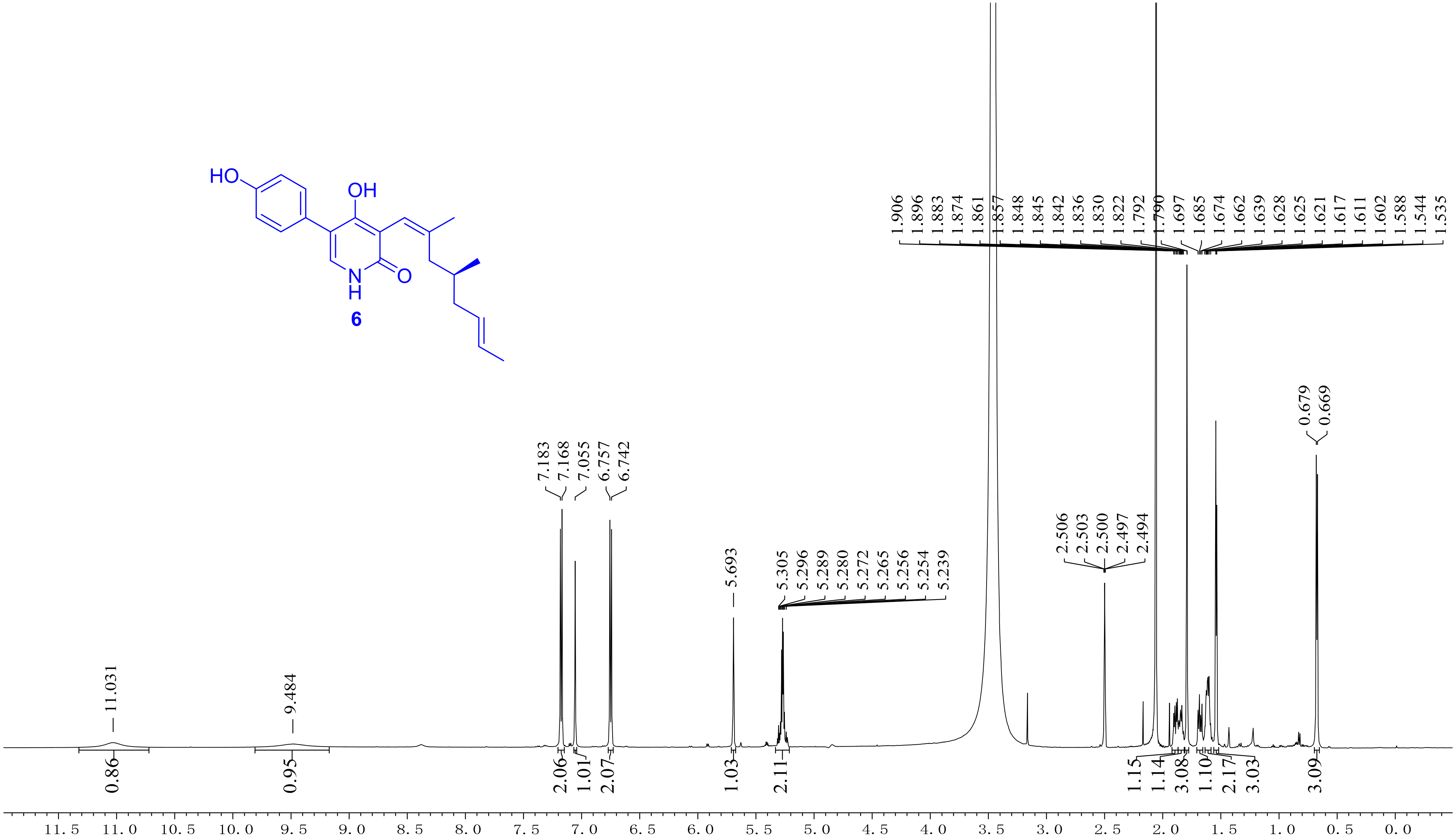

Figure S58. The ^{1}H NMR spectrum of compound **6** in DMSO-d_6 (600 MHz).

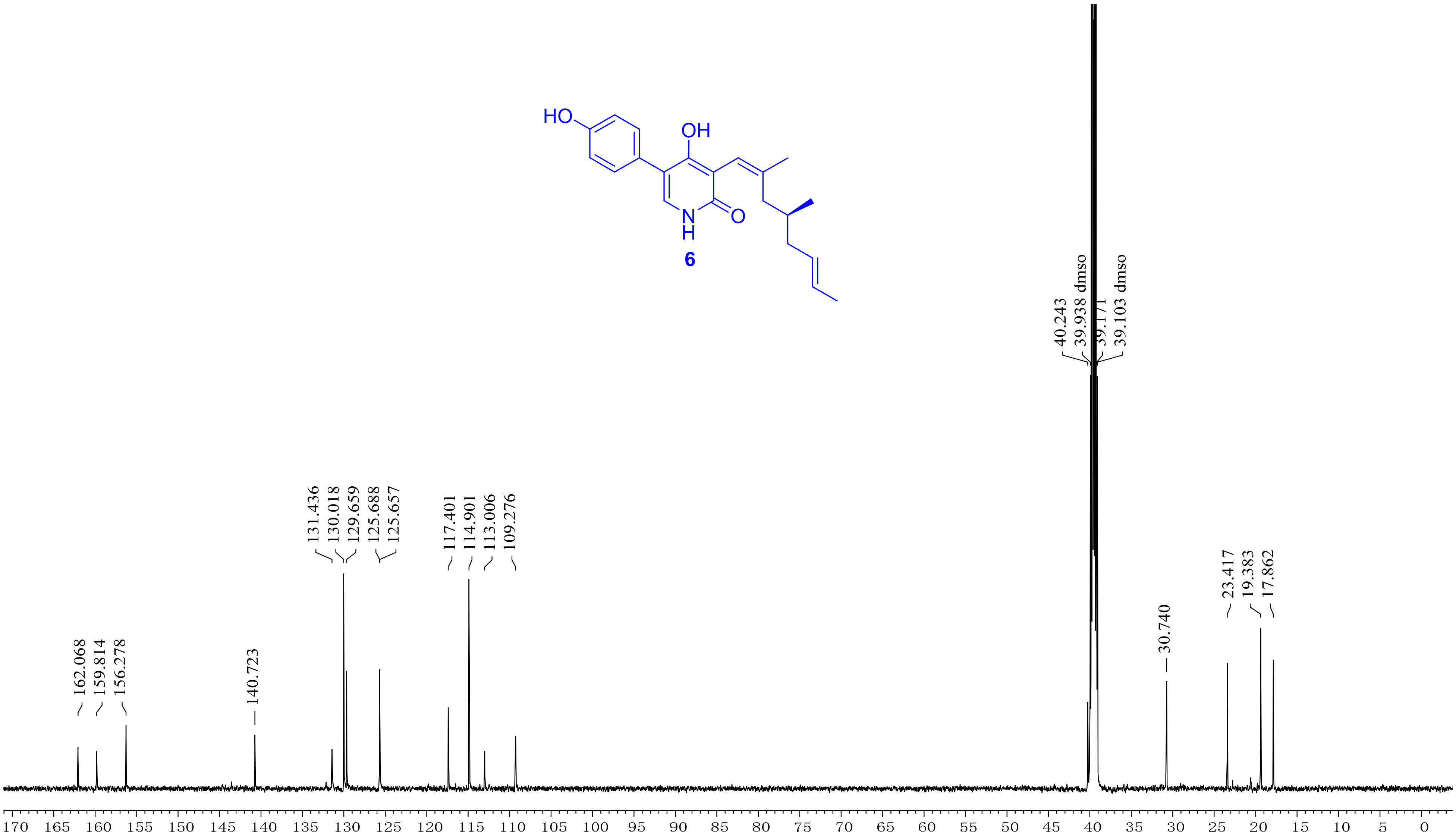

Figure S59. The ^{13}C NMR spectrum of compound **6** in DMSO-d_6 (150 MHz).

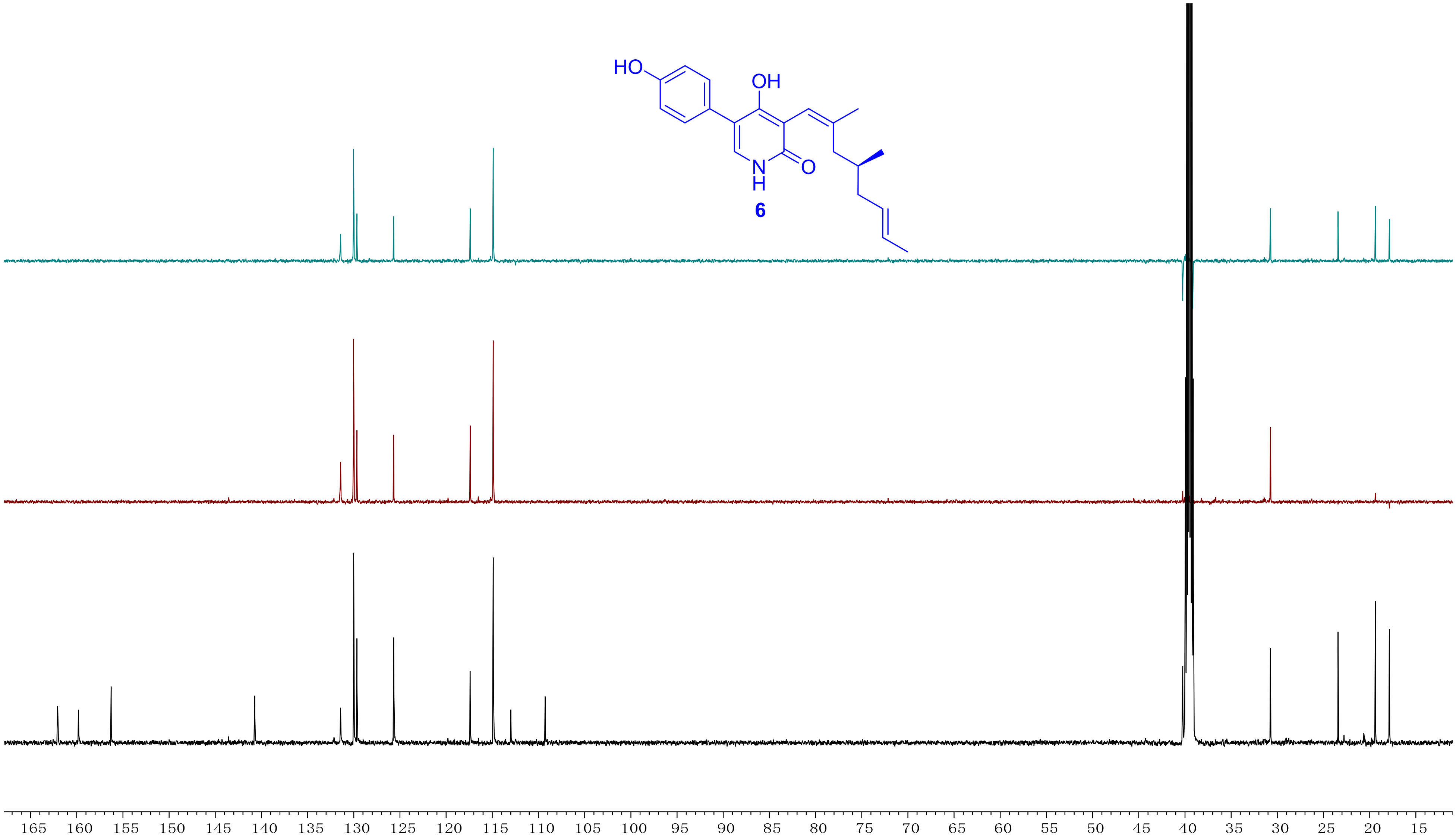

Figure S60. The DEPT spectrum of compound **6** in DMSO-d_6 (150 MHz).

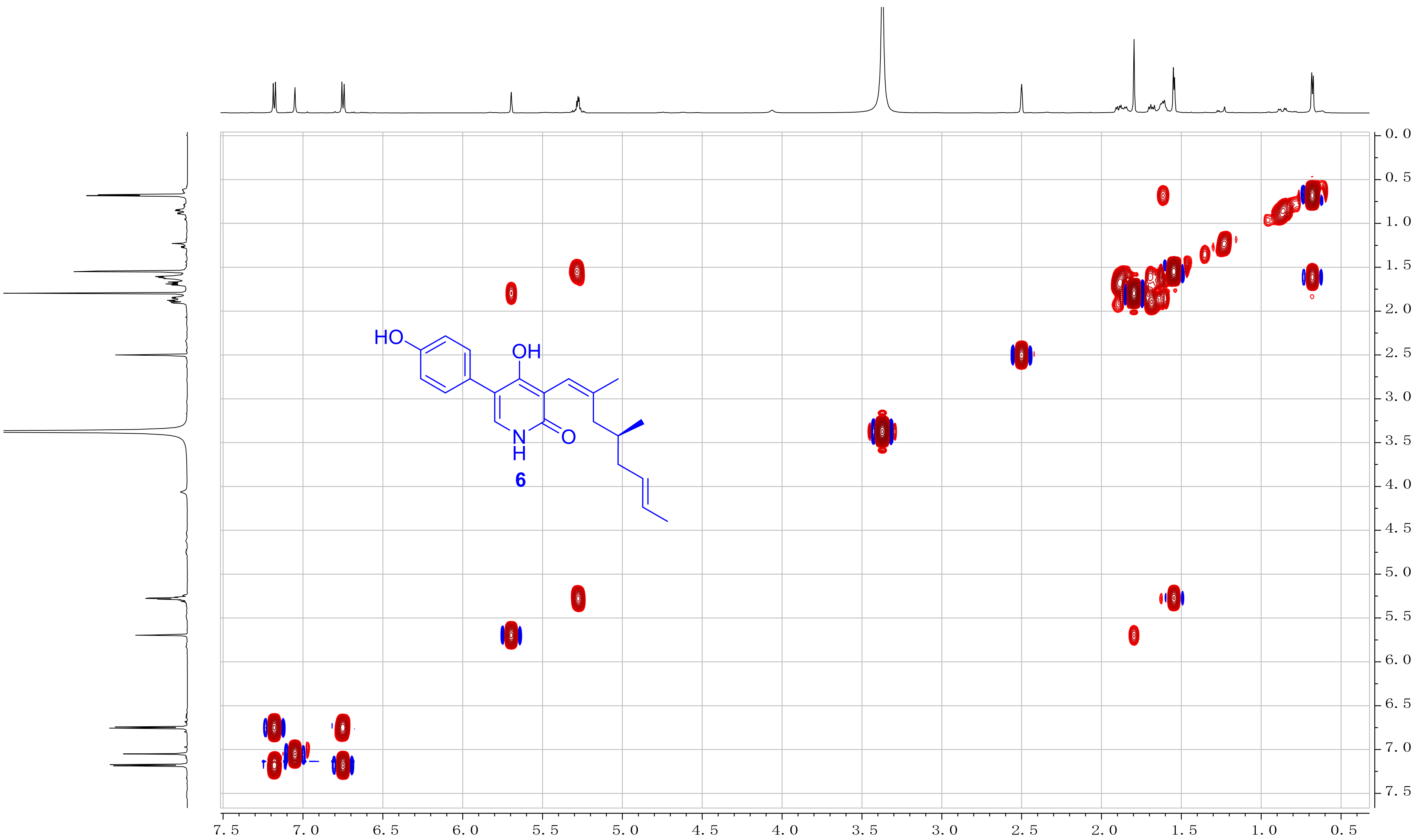

Figure S61. The ^{1}H-^{1}H COSY spectrum of compound **6** in DMSO-d_6 (600 MHz).

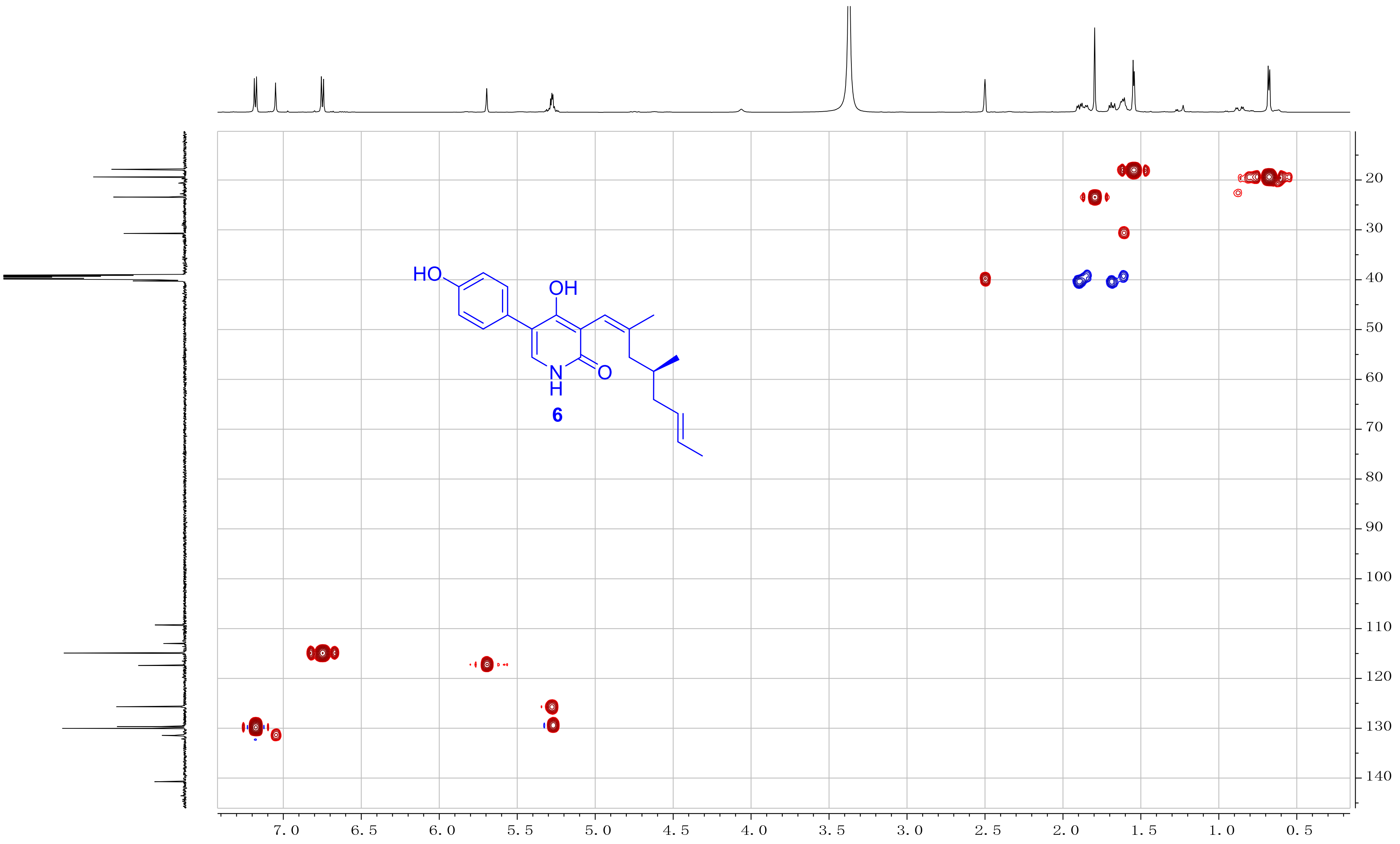

Figure S62. The HSQC spectrum of compound **6** in DMSO-d_6 (600 MHz).

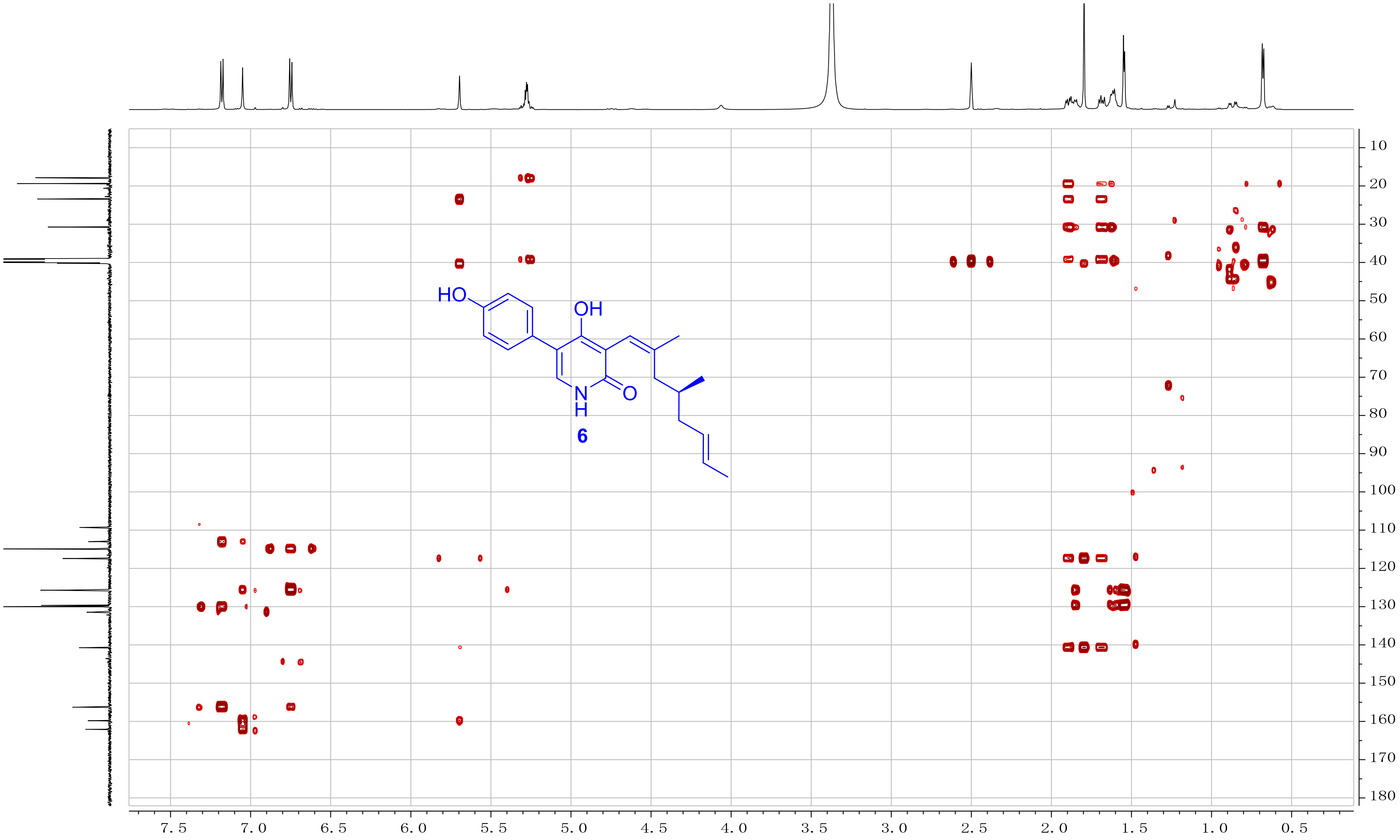

Figure S63. The HMBC spectrum of compound **6** in DMSO-d_6 (600 MHz).

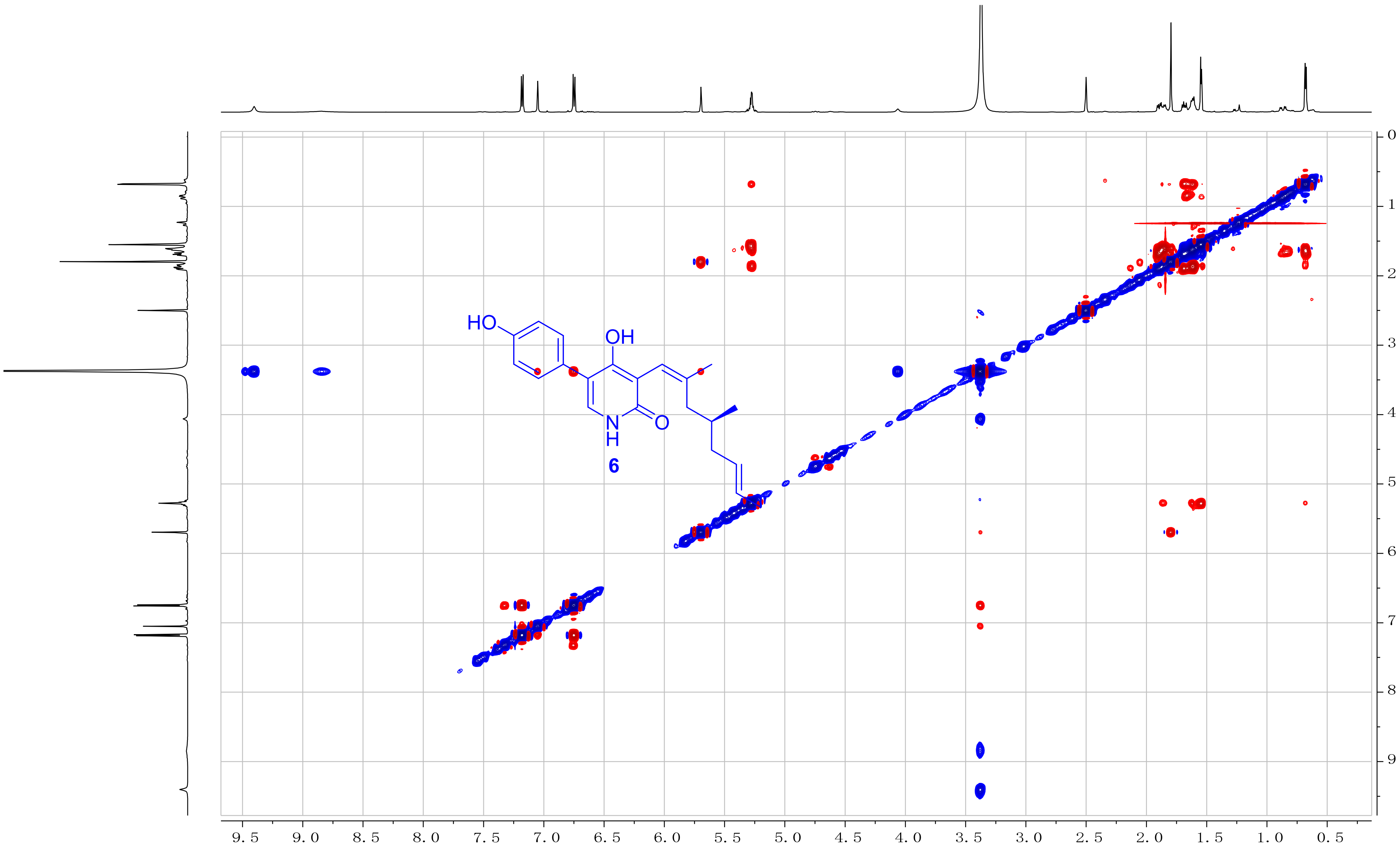

Figure S64. The ROESY spectrum of compound **6** in DMSO-d_6 (600 MHz).

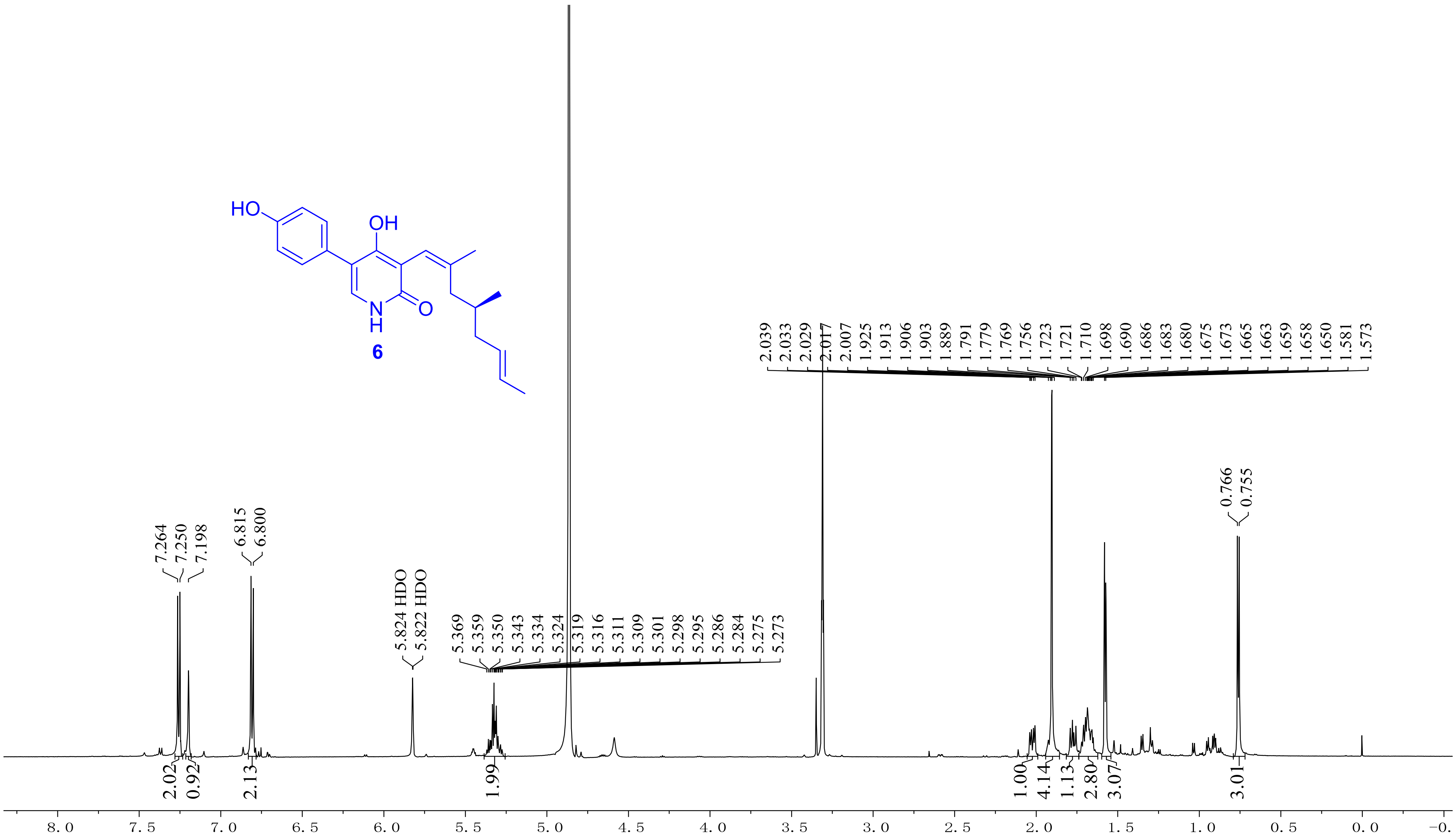

Figure S65. The ^{1}H NMR spectrum of compound **6** in CD_3OD (600 MHz).

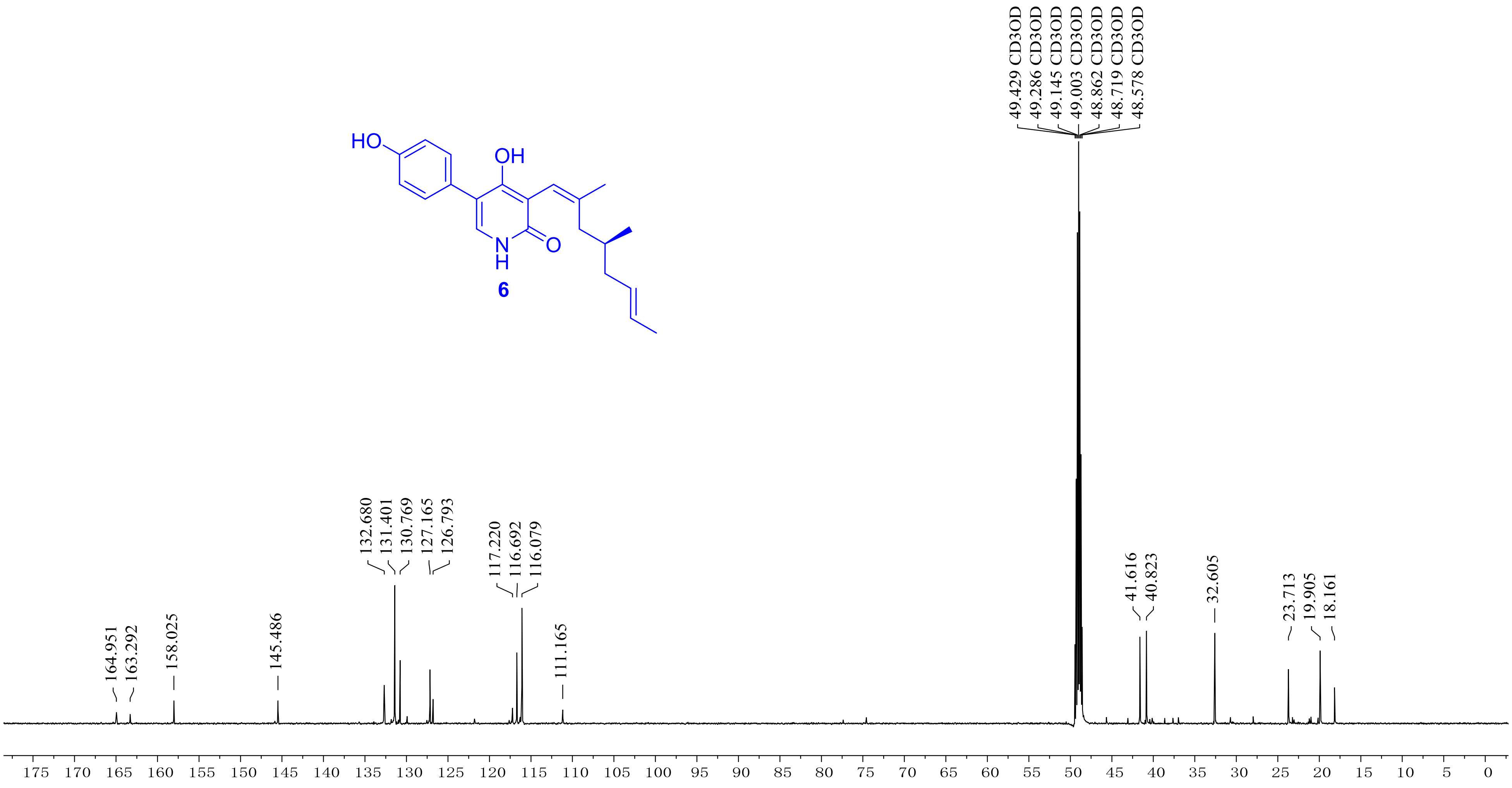

Figure S66. The ^{13}C NMR spectrum of compound **6** in CD_3OD (150 MHz)

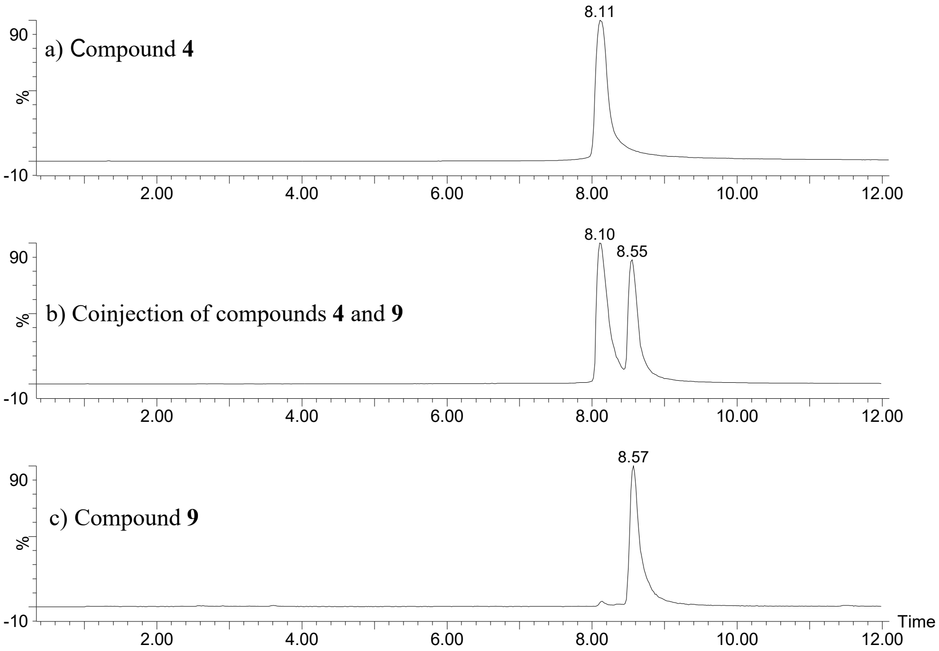

Figure S67. The LC-MS analysis of compounds **4** and **9**

References

1. *Molecular Operating Environment (MOE), 2019.01*; Chemical Computing Group Inc. 1010 Sherbooke St. West, Suite #910, Montreal, QC, Canada, H3A 2R7: 2019.

2.Gaussian 09, Revision A.1; Gaussian, Inc.: Wallingford CT, 2009.

3. Bruhn T, Schaumlöffel A, Hemberger Y, Bringmann G. SpecDis: quantifying the comparison of calculated and experimental electronic circular dichroism spectra. Chirality 2013;25:243-249.

4. Hao XM, Yu JQ, Wang YJ, Connolly JA, Liu YF, Zhang YQ, Yu LY, Cen S, Goss RJ M, Gan ML. Zelkovamycins B-E, cyclic octapeptides containing rare amino acid residues from an endophytic *Kitasatospora* sp. Org Lett. 2020;22:9346-9350.

5. Hao XM, Li SS, Ni J, Wang GY, Li F, Li Q, Chen SZ, Shu JC, Gan ML. Acremopeptaibols A–F, 16-residue peptaibols from the sponge-derived *Acremonium* sp. IMB18-086 cultivated with heat-killed *Pseudomonas aeruginosa*. J Nat Prod. 2021;84:2990-3000.

6.Li XB, Li L, Zhu RX, Li W, Chang WQ, Zhang LL, Wang XN, Zhao ZT, Lou HX. Tetramic Acids and Pyridone Alkaloids from the Endolichenic Fungus *Tolypocladium cylindrosporum.* J Nat Prod. 2015;78:2155-2160.

7. Wu B, Oesker V, Wiese J, Schmaljohann R, Imhoff J. F. Two new antibiotic pyridones produced by a marine fungus, *Trichoderma* sp. strain MF106. Mar Drugs. 2014;12:1208-1219.

8. Snider BB, Lu Q. Total Synthesis of (±)-Pyridoxatin. J Org Chem. 1994;59:8065-8070.

9. Zhang WY, Zhong Y, Yu Y, Shi DF, Huang HY, Tang XL, Wang YH, Chen GD, Zhang HP, Liu CL. 4-Hydroxy Pyridones from Heterologous Expression and Cultivation of the Native Host. J Nat Prod. 2020;83:3338-3346.

10. Wu XQ, Li J, Zhou X, Wang J, Tan YF, Mo JS, Liu S, Xu KP, Tan GS, Zhang W, Wang WX. Liver-cell protective pyridones from the fungi *Tolypocladium album* dws120. Phytochemistry. 2023;212:113730.

11. Jung Y, Kwon C, Kim T, Lee JW, Shin MK, Shim SH. Tetramic acid-motif natural products from a marine fungus *Tolypocladium cylindrosporum* FB06 and their anti-Parkinson activities. Mar Life Sci Technol. 2024;6:84-92.

12. Choi JW, Kwon C, Lee JW, Hur JS, Shin MK, Shim SH. Anti-Parkinsonian 4-hydroxy-2-pyridones from an endolichenic fungus, *Tolypocladium* sp. (strain CNC14). J Ind Microbiol Biotechnol. 2025;52:kuaf027.

13. Fukuda T, Igarashi Y, Sudoh Y, Tsuchiya Y, Okuda T, Matsuura N, Motojima A, Oikawa T. Tolypoalbin, a new tetramic acid from *Tolypocladium album* TAMA 479. J Antibiot. 2015;68:399-402.

14. Saitou N, Nei M. The neighbor-joining method: a new method for reconstructing phylogenetic trees. Mol Bio Evol. 1987;4:406-425.

15. Felsenstein J. Confidence limits on phylogenies: an approach using the Bootstrap. Evolution. 1985;39:783-791.

16. Kimura M. A simple method for estimating evolutionary rates of base substitutions through comparative studies of nucleotide sequences. J Mol Evol. 1980;16:111-120.

17. Kumar S, Stecher G, Tamura K. MEGA7: Molecular Evolutionary Genetics Analysis Version 7.0 for Bigger Datasets. Mol Bio Evol. 2016;33:1870-1874.
